# Ordinal pattern-based change point detection

**DOI:** 10.1007/s11749-025-00983-9

**Published:** 2025-08-15

**Authors:** Annika Betken, Giorgio Micali, Johannes Schmidt-Hieber

**Affiliations:** Department of Applied Mathematics, University of Twente, 7522 NB Enschede, The Netherlands

**Keywords:** Ordinal patterns, Turning rate, Change point detection

## Abstract

The ordinal patterns of a fixed number of consecutive values in a time series are the spatial ordering of these values. Counting how often a specific ordinal pattern occurs in a time series provides important insights into the properties of the time series. In this work, we prove the asymptotic normality of the relative frequency of ordinal patterns for time series with linear increments. Moreover, we apply ordinal patterns to detect changes in the distribution of a time series.

## Introduction

Ordinal patterns encode the spatial order of temporally ordered data points. Specifically, the ordinal pattern of order $r+1$ of time series data $\xi _0, \ldots , \xi _{r}$ refers to the permutation $(\pi _0,\ldots , \pi _r)$, where $\pi _j$ denotes the rank of $\xi _j$ within the values $\xi _0, \ldots \xi _r$ minus 1. For simplicity, we assume that the values of the data points are all different. Mathematically speaking, denoting with $\mathcal {S}_r$ the set of all $(r+1)!$ permutations of $\{0,\ldots , r\}$:

1 $$\begin{aligned} \Pi : \mathbb {R}^{r+1} \longrightarrow \mathcal {S}_r, \quad (\xi _0, \ldots , \xi _r ) \mapsto (\pi _0, \ldots , \pi _{r}), \end{aligned}$$

**Fig. 1:**
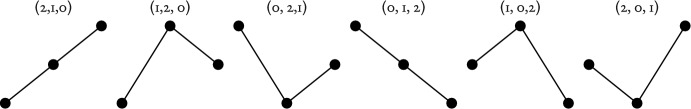
Six ordinal patterns of order $r+1=3$

We call $\Pi (\xi _0, \ldots , \xi _r)$ the ordinal pattern of $\xi _0, \ldots , \xi _r$. The six ordinal patterns of order $r+1=3$ are visualized in Fig. 1.

In modern time series literature, the concept of ordinal patterns was first introduced by Bandt and Pompe (2002) against the background of defining permutation entropy as a complexity measure of time series data. The latter is the Shannon entropy of the ordinal pattern distribution. As an estimate for ordinal pattern probabilities, we choose the relative frequencies of ordinal patterns in the underlying time series. The corresponding estimator has been studied by Sinn and Keller (2011) (for short-range dependent Gaussian time series) and by Betken et al. (2021) (for long-range dependent subordinated Gaussian time series). Moreover, Schnurr and Dehling (2017) measured nonlinear correlation of two time series, counting the number of coincident patterns in both time series, and as a by-product also studied this estimator for a class of short-range dependent time series.

One application of ordinal patterns is the classification of sleep stages based on EEG signals, crucial for medical purposes such as sleep quality assessment and sleep disorder diagnosis. Manual classification of sleep stages is tedious, subjective, time-consuming and error-prone. To address this, a plethora of machine learning-based techniques have emerged in recent years, often achieving high accuracy in classification; see, for example, Supratak et al. (2017), Chambon et al. (2018), Cheng et al. (2024). For a comprehensive review of automated sleep stage classification methods see Zhang et al. (2024). However, these methods typically lack interpretability and theoretical justification, may inherit biases present in training data and are often sensitive to small perturbations of the input; see Lipton (2018). To address these challenges, Bandt (2020) introduces an alternative approach to frequency classification in machine learning, based on the so-called turning rate. In a time series, the turning rate corresponds to the relative number of local maxima and minima in a fixed epoch of the series. More precisely, the turning rate corresponds to the frequency of observing one of the ordinal patterns (0, 2, 1), (1, 0, 2), (1, 2, 0), (2, 0, 1), each representing a local minimum or maximum, as illustrated in Fig. 1.

The aim of this work is to propose a hypothesis test for detecting changes in the distribution of a time series, based on variation of the corresponding turning rate series. For this, we derive limit theorems for estimators of ordinal patterns, assuming that the increments of a time series form a linear process, thereby allowing for different distributions and dependencies between observations than in previous works on ordinal patterns. (A more explicit comparison of our work to previous results will be given in Sect. 2.3.) As theoretical background, we establish empirical process limit theory for short- and long-range dependent multivariate linear time series. These general results, which are of independent interest, then serve as the basis for deriving the asymptotic properties of estimators for ordinal pattern probabilities.

The article is structured as follows: Sect. 2 establishes central limit theorems for empirical processes of short- and long-range dependent linear time series. In this section, ordinal patterns are formally introduced, and the derived limits are applied to obtain the limiting distribution for estimators of ordinal pattern probabilities. Sect. 3 extends these results to define the turning rate and proposes a statistical test for detecting structural changes in the distribution of a time series. Sect. 4 presents numerical experiments, including an application to EEG time series data. All proofs are provided in appendix.

### Notation

A generic time series is denoted as $(\xi _t)_{t\ge 0}$ and $\varvec{\xi }_t = (\xi _t, \xi _{t+1}, \ldots , \xi _{t+r})^\top$ represents the random vector of $r+1 \ge 2$ consecutive time series values. The time series generated by the increments of $(\xi _t)_{t\ge 0}$ is denoted by $(X_t)_{t\ge 1}$, where $X_t := \xi _t - \xi _{t-1}$ and ${\textbf {X}}_t = (X_t, \ldots , X_{t+r-1})^\top$. The set of invertible matrices of size $r$ is denoted by $\text {GL}(\mathbb {R}, r)$. The symbol $\bar{X}_n$ denotes the average $\bar{X}_n := \frac{1}{n}\sum _{j=1}^n X_j$. The space $\ell ^m$ denotes the set of real sequences $(a_j)_{j\in \mathbb {N}}$ satisfying $\sum _{j\in \mathbb {N}}|a_j|^m < \infty$, $L^\infty (\mathbb {R})$ is the set of all real-valued functions defined on $\mathbb {R}$ that are almost surely bounded, and ${\mathcal {C}}^1(\mathbb {R})$ consists of continuously differentiable functions on $\mathbb {R}$. The gradient is $\nabla f ({\textbf {x}}) := \left( \partial _1 f({\textbf {x}}), \ldots , \partial _p f({\textbf {x}}) \right) .$ Throughout the article, $\Vert \cdot \Vert$ denotes the Euclidean norm for vectors and the spectral norm for matrices. An i.i.d. sequence of centered $\mathbb {R}^r$-valued random vectors $({\textbf {Z}}_j)_{j\in \mathbb {Z}}$ is called multivariate zero-mean white noise process with $r\times r$ covariance matrix $\varvec{\Sigma }=\mathop {\operatorname {Cov}}({\textbf {Z}}_1)$. Lastly, the indicator function of an event *A* is denoted by $\mathbbm {1}(A).$

## Main theoretical results

Let $X_t=\sum _{j=0}^\infty a_j Z_{t-j},$ $t \in \mathbb {Z}$, be a linear process with deterministic real coefficients $a_j$ and i.i.d. random variables $(Z_j)_{j \in \mathbb {Z}}$. We also assume that $\mathbb {E}[Z_j]=0$ and $\mathop {\operatorname {Var}}(Z_j)=\sigma _Z^2$ for all *j*,  implying that $X_t$ is centered. By Kolmogorov’s three-series theorem, $X_t$ exists almost surely if $\sum _{j=0}^\infty a_j^2< \infty$; see Wu (2002). Since the innovations $Z_j$ are not assumed to be Gaussian, the process $X_t$ can be non-Gaussian. The monograph of Rosenblatt (2000) highlights that allowing for non-Gaussianity makes the class of processes much richer.

It follows from the definition that the linear process $(X_t)_{t\ge 1}$ is moreover (strictly) stationary. As such, it admits an autocovariance function $\gamma _X(k)$ (for $k \in \mathbb {Z}$) and a corresponding spectral density $f_X(\lambda ) = (2\pi )^{-1} \sum _{k=-\infty }^{\infty } \gamma _X(k) \exp (-ik\lambda )$ with $\lambda \in [-\pi ,\pi ]$. There are various definitions for short- and long-range dependence; see for instance (Samorodnitsky 2007). While sometimes more specific growth conditions on the spectral density are imposed, they all agree that the process $(X_t)_{t\ge 1}$ can be categorized as exhibiting long-range dependence, short-range dependence or antipersistence, if, as $|\lambda | \rightarrow 0$, the spectral density converges to infinity, a finite positive constant or zero, respectively. Since $2\pi f_X(0) = \sum \gamma _X(k)$, these conditions can also be equivalently stated in terms of the sum of autocovariances $\gamma (k)$. For a linear process, $\gamma _X(k)=\mathbb {E}[X_tX_{t+k}]=\sum _{j, i\ge 0} a_j a_i \mathop {\operatorname {Cov}}(Z_{t-j},Z_{t+k-i})=\sum _{j\ge 0} a_j a_{|k|+j}$ and $\sum _{k=-\infty }^\infty \gamma _X(k)=(\sum _{j\ge 0} a_j)^2.$ This shows that long-range dependence happens if $\sum _{j\ge 0} a_j=\infty ,$ short-range dependence occurs if $\sum _{j\ge 0} a_j$ is a finite constant, and antipersistence corresponds to $\sum _{j\ge 0} a_j=0.$ Standard textbooks restrict these classes further by adding decay conditions; see, e.g., Section 2.1.1.3 in Beran et al. (2013). Moreover, for a linear process, different types of memory may be defined via the spectral density or by requiring the summability of $(\gamma _X(k))_{k\in \mathbb {Z}}$. For instance, Beran et al. (2013) show that the conditions $\sum _{j=0}^\infty |a_j| < \infty \quad \text {and} \quad \sum _{j=0}^\infty a_j \ne 0$ imply $\sum _{k \in \mathbb {Z}} |\gamma (k)| < \infty$ and $\sum _{k \in \mathbb {Z}} \gamma (k) > 0$ (see their Lemma 4.14). The multivariate time series ${\textbf {X}}_t = \sum _{j\in \mathbb {Z}} {\textbf {A}}_{j} {\textbf {Z}}_{t-j},$ where $({\textbf {Z}}_j)_{j \in \mathbb {Z}}$ is an i.i.d. $\mathbb {R}^r$-valued multivariate white noise process with matrix coefficients ${\textbf {A}}_j$ of size $r\times r$, is said to have short memory if, for any matrix norm $\Vert \cdot \Vert$, $\sum _{j \in \mathbb {Z}}\Vert {\textbf {A}}_{j}\Vert <\infty .$ In this work, we adopt the notion of long-range dependence proposed by Kechagias and Pipiras (2015) (Definitions 2.1 and 2.2). Proposition 3.1 in Kechagias and Pipiras (2015) shows that if the sequence of coefficient matrices ${\textbf {A}}_{j}$ satisfies

2 $$\begin{aligned} \textbf{A}_j\sim j^{d-1}\textbf{A}_ \infty \quad \text {as} \quad j\rightarrow \infty , \end{aligned}$$

for some matrix ${\textbf {A}}_{\infty }$ and a value $d\in \left( 0, \frac{1}{2}\right)$, then the linear process $({\textbf {X}}_t)_{t\ge 1}$ exhibits long-range dependence. Here, for $r\times r$ matrices ${\textbf {U}}_{j}$ and ${\textbf {V}}_j$, we write ${\textbf {U}}_{j} \overset{j \rightarrow \infty }{\sim }\ {\textbf {V}}_j$, if $u_{ps,j}/v_{ps,j} \rightarrow 1$ as $j \rightarrow \infty$ for all entries $(p,s)$ of ${\textbf {U}}_{j}$ and ${\textbf {V}}_j$.

Our aim is to establish central limit theorems for the relative frequencies of linear processes and apply these to estimators of ordinal pattern probabilities. In our work, we focus on processes $(\xi _t)_{t\ge 0}$ whose increments $X_t=\xi _t-\xi _{t-1}$ form a stationary process. This assumption results from the observation that the ordinal pattern of $(\xi _t, \xi _{t+1}, \ldots , \xi _{t+r-1})$ only depends on the increments $X_s$, $s=t+1, \ldots , t+r-1$. Stationarity of the increments is needed for guaranteeing that the probability of each pattern remains the same throughout the time series. While stationarity of the entire time series implies stationarity of the increments, the assumption of stationary increments is a more general condition. Moreover, Lemma 2 shows that the increment process allows us to interpret the ordinal patterns $(\pi _0, \ldots , \pi _r)$ of $(\xi _t)_{t \ge 0}$ as the ordinal patterns of a 1linear transformation of the increments $(X_t)_{t\ge 1}$. Assuming that the increments form a linear process implies that the vectors ${\textbf {X}}_t = (X_{t+1}, \ldots , X_{t+r})^\top$ form a multivariate linear process, and for an $s \times r$ matrix ${\textbf {V}}$, $({\textbf {V}} {\textbf {X}}_t)_t$ forms a multivariate linear process as well. Based on this setup, we establish limiting theorems for multivariate linear processes $({\textbf {X}}_t)_{t \ge 1}$ (see Sect. 2.1), using a martingale decomposition approach.

### Central limit theorems for relative frequencies in linear processes

Let $r$ be a positive integer, and assume that for $n \ge r - 1$, we observe a linear process $(X_t)_{t \ge 1}$ for $t = 1, \ldots , n + r - 1$. A common approach to estimate the probability $p(u_0, \ldots , u_{r-1}) := \mathbb {P}(X_{t} \le u_0, X_{t+1} \le u_1, \ldots , X_{t+r-1} \le u_{r-1})$ is by using the relative frequency of this event in the sample, expressed as:

3 $$\begin{aligned} \widehat{p}_n(u_0, \ldots , u_{r-1}):= \frac{1}{n} \sum _{t=1}^{n} \mathbbm {1}\big (X_{t} \le u_0, \ldots , X_{t+r-1} \le u_{r-1}\big ). \end{aligned}$$

By taking the expectation, we conclude that this is an unbiased estimator for the probability $p(u_0, \ldots , u_{r-1})$.

We now proceed to derive (functional) central limit theorems for the relative frequencies (3). To account for the $r$ inequalities, it is convenient to first reformulate the univariate linear time series as an $r$-dimensional multivariate linear process.

#### Lemma 1

Given a linear process defined by $X_t=\sum _{j= 0 }^\infty a_j Z_{t-j}$, the multivariate process ${\textbf {X}}_t:=\left( X_t, X_{t+1}, \ldots , X_{t+r-1}\right) ^{\top }$ is linear and satisfies

4 $$\begin{aligned} {\textbf {X}}_t= \sum _{j= 0 }^\infty {\textbf {A}}_{j} {\textbf {Z}}_{t-j}\; \end{aligned}$$

with diagonal coefficient matrices

$$\begin{aligned} {\textbf {A}}_{j} = \left( \begin{array}{ccccc} a_{j-r+1} & 0 & \cdots & 0 \\ 0& a_{j-r+2} & \ddots & \vdots \\ \\ \vdots & \ddots & \ddots & 0 \\ 0 & \cdots & 0 & a_j\\ \end{array}\right) \end{aligned}$$

(setting $a_i:=0$ whenever $i <0$) and i.i.d. innovations ${\textbf {Z}}_{t-j}= Z_{t-j+r-1}\left( 1, \ldots , 1\right) ^{\top }$ with variance ${\textbf {E}}$, where ${\textbf {E}}$ denotes the $r\times r$ matrix with all entries equal to 1.

#### Proof

Changing *j* to $j-r+s+1,$ we find for any $s=0,\ldots , r-1,$ $X_{t+s}=\sum _{j= 0 }^\infty a_j Z_{t+s-j}=\sum _{j=0}^\infty a_{j-r+s+1}Z_{t-j+r-1}$ with $a_i=0$ whenever $i<0.$ $\square$

We can now rewrite the relative frequency estimator $\widehat{p}_n(u_1,\ldots ,u_r)$ defined in (3) as

5 $$\begin{aligned} \widehat{p}_n({\textbf {u}}) :=\frac{1}{n} \sum _{t=1}^{n} \mathbbm {1}\big ({\textbf {X}}_t\le {\textbf {u}}\big ), \end{aligned}$$

with ${\textbf {u}}:=(u_1,\ldots ,u_r)^\top$ and $\le$ understood component-wise. This is an estimator for the probability $p({\textbf {u}}) :=P\big ({\textbf {X}}_t\le {\textbf {u}}\big ).$

In a next step, we prove a functional central limit theorem for multivariate linear processes with general covariance matrix for ${\textbf {Z}}_{j}$ and general coefficient matrix ${\textbf {A}}_{j}$ satisfying the following assumption:

#### Assumption 1

There exists a $J\in \mathbb {N}$ and an invertible $r\times r$ matrix ${\textbf {D}}$ such that ${\textbf {D}}\sum _{j=0}^J {\textbf {A}}_{j} {\textbf {Z}}_{t-j}$ is a vector of independent random variables with bounded Lebesgue density. Furthermore, ${\textbf {A}}_{j} \ne {\textbf {0}}_{r\times r}$ for $j=0,\ldots , J.$

If $A_0$ is an invertible matrix and ${\textbf {Z}}_{t-j}$ consists of independent random variables with bounded Lebesgue density, the condition is satisfied with $J=0$ and $D=A_0^{-1}.$ If the multivariate process has been generated by a univariate linear process as in the setting of Lemma 1, $a_0\ne 0$, and the innovations admit a bounded Lebesgue density, then the condition holds with $J=r-1.$ Indeed, note that

6 $$\begin{aligned} \sum _{j=0}^{r-1} {\textbf {A}}_{j} {\textbf {Z}}_{t-j}&= \sum _{j=0}^{r-1} Z_{t-j+r-1} {\textbf {A}}_{j} \left( \begin{array}{c} 1 \\ 1 \\ \vdots \\ 1 \end{array}\right) \nonumber \\&=\left( \begin{array}{ccccc} a_0 & 0 & \cdots & 0 \\ a_1& a_0 & \ddots & \vdots \\ \vdots & & \ddots & 0 \\ a_{r-1} & \cdots & a_1 & a_0\\ \end{array}\right) \left( \begin{array}{c} Z_t \\ Z_{t+1} \\ \vdots \\ Z_{t+r-1} \end{array} \right) =: {\textbf {B}} {\textbf {Z}}_{t,r}. \end{aligned}$$

Since $a_0\ne 0,$ the triangular matrix on the right-hand side is invertible and ${\textbf {D}}$ can be taken as its inverse which then gives ${\textbf {D}}\sum _{j=0}^J {\textbf {A}}_{j} {\textbf {Z}}_{t-j}=(Z_t,\ldots ,Z_{t+r-1})^\top =:{\textbf {Z}}_{t,r}.$ Since by assumption, the innovations $Z_t$ admit a bounded Lebesgue density, this verifies Assumption 1 in this case.

Let $\Vert \cdot \Vert$ be the operator norm and denote by $$\overset{\mathcal {D}[0,1]}{\Longrightarrow }\ $$ the convergence in distribution in the Skorokhod space $\mathcal {D}[0,1]$ with respect to the Skorokhod topology; see Billingsley (1968).

#### Theorem 1

(Short-Range Dependence) Let ${\textbf {X}}_t= \sum _{j= 0 }^\infty {\textbf {A}}_{j} {\textbf {Z}}_{t-j}$ be a multivariate linear process satisfying $\sum _{j=0}^\infty \Vert {\textbf {A}}_{j}\Vert < \infty$ and Assumption 1. Then, for any *r*-dimensional vector ${\textbf {u}}=(u_0, \ldots , u_{r-1})^\top ,$

7 $$\begin{aligned} \frac{1}{\sqrt{n}}\sum _{t=1}^{[n\tau ]} \Big (\mathbbm {1}\big ({\textbf {X}}_t\le {\textbf {u}}\big ) -p({\textbf {u}})\Big )\overset{\mathcal {D}[0,1]}{\Longrightarrow }\ \sigma B(\tau ), \quad \tau \in [0,1], \end{aligned}$$

with variance $$\sigma ^2:= \mathop {\operatorname {Var}}\left( \mathbbm {1}( {\textbf {X}}_1 \le {\textbf {u}}) \right) +2\sum _{j=1}^{\infty }\mathop {\operatorname {Cov}}\left( \mathbbm {1}( {\textbf {X}}_1 \le {\textbf {u}}), \mathbbm {1}( {\textbf {X}}_{1+j} \le {\textbf {u}}) \right) $$.

In particular, for $\tau =1$, we obtain

8 $$\begin{aligned} \sqrt{n}\Big (\widehat{p}_n({\textbf {u}}) - p({\textbf {u}})\Big )\xrightarrow {\mathcal {D}} \mathcal {N}(0,\sigma ^2). \end{aligned}$$

The proof of Theorem 1 is provided in Appendix A.

#### Remark 1

To prove Theorem 1, one needs to modify the second assumption of Theorem 12 (Theorem 2.1 in Furmańczyk 2007) as it is not satisfied for the indicator function $G({\textbf {X}}_t):={\textbf {1}}\big ({\textbf {X}}_t\le {\textbf {u}}\big )$. Lemma 3 shows that Assumption 1 in Theorem 1 ensures that the second assumption of Theorem 12 still holds for indicators.

We now discuss the case where the underlying multivariate linear process exhibits long-range dependence.

#### Theorem 2

(Long-Range Dependence) Let ${\textbf {X}}_t = \sum _{j=0}^\infty {\textbf {A}}_{j} {\textbf {Z}}_{t-j}$ be a multivariate linear process satisfying ${\textbf {A}}_{j} \sim j^{d-1} {\textbf {A}}_{\infty }$ as $j \rightarrow \infty$, where ${\textbf {A}}_{\infty } \in \text {GL}(\mathbb {R}, r)$ and $d \in (0,1/2)$. The innovations $( {\textbf {Z}}_j )_{j \in \mathbb {Z}}$ are i.i.d. with variance $\varvec{\Sigma }$, and are assumed to satisfy the moment condition $\mathbb {E}[\Vert {\textbf {Z}}_1\Vert ^4] < \infty$. Define the cumulative distribution function $p_s(\cdot ) = \mathbb {P}( \sum _{j=0}^s {\textbf {A}}_{j} {\textbf {Z}}_{t-j} \le \cdot )$. If there exists a positive integer $s_0$ such that

9 $$\begin{aligned} \sup _{{\textbf {x}}\in \mathbb {R}^r} \, \max _{s\ge s_0} \left( |p_s({\textbf {x}}) | + \sum _{i=1}^r | \partial _i p_s({\textbf {x}}) | + \sum _{i, j=1}^r | \partial ^2_{i,j} p_s({\textbf {x}}) | \right) <\infty , \end{aligned}$$

then,

10 $$\begin{aligned} &  n^{1/2-d}\Big (\widehat{p}_n({\textbf {u}}) - p({\textbf {u}})\Big )\nonumber \\ &  \quad \xrightarrow {\mathcal {D}} \mathcal {N}\bigg (0,\frac{\Gamma (d)^2 }{\Gamma (2d+2)\cos (\pi d)}( \nabla p({\textbf {u}}))^{\top } {\textbf {A}}_{\infty } \varvec{\Sigma } {\textbf {A}}_{\infty }^{\top } \nabla p({\textbf {u}}) \bigg ). \end{aligned}$$

The proof of Theorem 2 can be found in Appendix B. A key ingredient of the proof is to establish a so-called reduction principle, stating that for any ${\textbf {u}} \in \mathbb {R}^r,$

11 $$\begin{aligned} n^{\frac{1}{2} - d} \left| \frac{1}{n} \sum _{t=1}^n \mathbbm {1}\big ({\textbf {X}}_t \le {\textbf {u}}\big ) - p({\textbf {u}}) + (\nabla p({\textbf {u}}))^\top \bar{{\textbf {X}}}_n \right| \xrightarrow {\mathbb {P}} 0. \end{aligned}$$

Proposition 9 in Appendix B provides a simple and operational criterion to verify (9).

### Ordinal patterns

We apply the central limit theorems derived in the previous section to ordinal patterns. Consider a univariate time series $(\xi _t)_{t\ge 0}$.

#### Definition 1

Let $S_r$ denote the set of permutations of $\{0, \ldots , r\}$, which we write as $(r + 1)$-tuples containing each of the numbers $0, \ldots , r$ exactly once. By the *ordinal pattern* of order $r$, we refer to the permutation

$$\begin{aligned} \Pi (\xi _0, \ldots , \xi _r) = (\pi _0, \ldots , \pi _r) \in S_r, \end{aligned}$$

which satisfies

$$\begin{aligned} \xi _{\pi _0} \ge \cdots \ge \xi _{\pi _r}, \end{aligned}$$

and $\pi _{i-1} > \pi _i$ if $\xi _{\pi _{i-1}} = \xi _{\pi _i}$ for $i = 1, \ldots , r - 1$. We say that the time series $(\xi _t)_{t\ge 0}$ has ordinal pattern $(\pi _0,\ldots ,\pi _r)\in S_r$ at time *t*,  if $\Pi (\xi _t, \ldots , \xi _{t+{r}})=(\pi _0, \ldots , \pi _{r})$.

#### Remark 2

Ties are formally accounted for in Definition 1: if two values are equal, the one with the larger index is ranked lower. In the setting adopted throughout this manuscript, such ties occur with probability zero, as the underlying process is continuously distributed. However, in applications involving discrete-valued time series, ties may occur with positive probability. In such cases, alternative definitions of ordinal patterns have been proposed that extend the classical permutation set $S_r$ to include patterns with repeated ranks; see, e.g., Schnurr and Fischer (2022) and Weiß and Schnurr (2024).

Ordinal patterns look for specific orderings of the time series values over $r+1$ consecutive time instances. The frequency at which they occur can provide some insights about the distributional properties of the time series.

Following Sinn and Keller (2011) and Betken et al. (2021), we can rewrite ordinal patterns as inequalities of the increment process $X_t:=\xi _t-\xi _{t-1}.$ Let the row vector $e_k$ be the *k*-th standard basis vector of $\mathbb {R}^{r+1}$ and recall that ${\textbf {u}}\le 0$ for a vector ${\textbf {u}}$ means that all entries of ${\textbf {u}}$ are $\le 0$.

#### Lemma 2

The time series $(\xi _t)_{t\ge 0}$ has ordinal pattern $\pi =(\pi _0,\ldots ,\pi _r)\in S_r$ at time *t*,  if and only if the stacked increment process ${\textbf {X}}_{t+1}=(X_{t+1},\ldots ,X_{t+r})^\top$ satisfies ${\textbf {V}}_\pi {\textbf {X}}_{t+1}\le 0$ with

12 $$\begin{aligned} {\textbf {V}}_\pi := \underbrace{\left( \begin{array}{cccccc} -1 & 1 & 0 & 0 & \cdots & 0\\ 0 & -1 & 1 & 0 & \cdots & 0\\ \vdots & & \ddots & \ddots & & \vdots \\ 0 & \cdots & 0 & -1 & 1 & 0\\ 0 & \cdots & 0 & 0 & -1 & 1 \\ \end{array}\right) }_{r \times (r+1)} \underbrace{ \left( \begin{array}{c} e_{\pi _0+1} \\ e_{\pi _1+1} \\ \vdots \\ \vdots \\ e_{\pi _{r}+1} \\ \end{array}\right) }_{(r+1) \times (r+1)} \underbrace{ \left( \begin{array}{cccc} 0 & \cdots & \cdots & 0 \\ 1& \ddots & & \vdots \\ \vdots & \ddots & \ddots & \vdots \\ \vdots & & \ddots & 0\\ 1 & \cdots & \cdots & 1 \\ \end{array}\right) }_{(r+1)\times r}. \end{aligned}$$

In other words, $\left\{ \Pi (\xi _t, \xi _{t+1}, \ldots , \xi _{t+r}) =\pi \right\} = \{ {\textbf {V}}_\pi {\textbf {X}}_{t+1}\le {\textbf {0}}\}.$ The matrix ${\textbf {V}}_\pi$ is moreover invertible.

#### Proof

Observe that the matrix vector product of the rightmost matrix in (12) with ${\textbf {X}}_{t+1}$ gives the column vector $(\xi _t-\xi _t,\xi _{t+1}-\xi _t,\ldots ,\xi _{t+r}-\xi _t)^\top .$ Multiplying this vector from the left with the $(r+1)\times (r+1)$ permutation matrix that occurs in the middle of the matrix product that defines ${\textbf {V}}_\pi ,$ reorders the entries and gives $(\xi _{t+\pi _0}-\xi _t,\xi _{t+\pi _1}-\xi _t,\ldots ,\xi _{t+\pi _r}-\xi _t)^\top .$ Thus, ${\textbf {V}}_\pi {\textbf {X}}_{t+1}=(\xi _{\pi _1}-\xi _{\pi _0},\ldots ,\xi _{\pi _r}-\xi _{\pi _{r-1}})^\top .$ Hence, ${\textbf {V}}_\pi {\textbf {X}}_{t+1}\le 0$ if and only if $\xi _{t+\pi _{i+1}}- \xi _{t+\pi _{i}}\le 0,$ for all $i=0, \ldots , r-1.$ To see that ${\textbf {V}}_\pi$ is invertible, one can use again the definition of ${\textbf {V}}_\pi$ as a product of three matrices in (12) and observe that the kernel of the product of the two matrices on the left is $(1,\ldots ,1)^\top$, whereas the image of the rightmost matrix consists of all vectors with the first entry equaling zero. Since the intersection of this kernel and this image is the zero vector, the kernel of ${\textbf {V}}_\pi$ is trivial, and thus, ${\textbf {V}}_\pi$ is invertible and the lemma holds. $\square$

The relative frequency of the ordinal patterns $\pi$ occurring in the time series $\xi _0,\ldots ,\xi _{n+r-1}$ is

13 $$\begin{aligned} \widehat{p}_n(\pi ) = \frac{1}{n}\sum _{t=0}^{n-1} \mathbbm {1} \left( \Pi (\xi _t, \ldots , \xi _{t+r})= \pi \right) =\frac{1}{n}\sum _{t=1}^n \mathbbm {1}\big ({\textbf {V}}_\pi {\textbf {X}}_t\le {\textbf {0}}\big ). \end{aligned}$$

This is an estimator of the probability that the ordinal pattern $\pi$ occurs, which is

14 $$\begin{aligned} p(\pi ):=\mathbb {P}\left( \Pi (\xi _0, \ldots , \xi _{r})= \pi \right) = \mathbb {P}\big ({\textbf {V}}_\pi {\textbf {X}}_1\le {\textbf {0}}\big ). \end{aligned}$$

To analyze the estimator $\hat{p}_n(\pi )$, we assume that the increment process $(X_t)_{t\ge 1}$ is a linear process $X_t=\sum _{j=0}^\infty b_j Z_{t-j}.$ Applying Lemma 1, it then follows that ${\textbf {X}}_t=(X_t,X_{t+1},\ldots ,X_{t+r-1})^\top$ is a multivariate linear process that can be written as ${\textbf {X}}_t=\sum _{j=0}^\infty {\textbf {B}}_j {\textbf {Z}}_{t-j}$ with diagonal coefficient matrix

15 $$\begin{aligned} {\textbf {B}}_j = \left( \begin{array}{ccccc} b_{j-r+1} & 0 & \cdots & 0 \\ 0& b_{j-r+2} & \ddots & \vdots \\ \\ \vdots & \ddots & \ddots & 0 \\ 0 & \cdots & 0 & b_j\\ \end{array}\right) \end{aligned}$$

(setting $b_\ell :=0$ whenever $\ell <0$) and i.i.d. innovations ${\textbf {Z}}_{t-j}= Z_{t-j+r-1}\left( 1, \ldots , 1\right) ^{\top }$ with variance ${\textbf {E}}$, where ${\textbf {E}}$ denotes the $r\times r$-matrix with all entries equal to 1. Moreover, ${\textbf {V}}_\pi {\textbf {X}}_t=\sum _{j=0}^\infty {\textbf {A}}_{j} {\textbf {Z}}_{t-j}$ with ${\textbf {A}}_{j}={\textbf {V}}_\pi {\textbf {B}}_j.$ Thus, also $({\textbf {V}}_\pi {\textbf {X}}_t)_t$ is a multivariate linear process. By applying now the central limit theorems from the previous section to $({\textbf {V}}_\pi {\textbf {X}}_t)_t$, we obtain central limit theorems for the relative frequencies ${\widehat{p}}_n(\pi )$ of ordinal patterns. A consequence of Theorem 1 is the following theorem:

#### Theorem 3

(Short-Range Dependence) Let $(\xi _t)_{t\ge 0}$ be a time series whose increments $X_t=\xi _t-\xi _{t-1}$ form a linear process $X_t=\sum _{j=0}^\infty b_jZ_{t-j}$ with $\sum _{j=0}^\infty |b_j|<\infty ,$ and $b_0\ne 0.$ If $Z_1$ admits a continuous and bounded probability density function, then,

16 $$\begin{aligned} \frac{1}{\sqrt{n}}\sum _{t=1}^{[n\tau ]} \Big (\mathbbm {1} \left( \Pi (\xi _{t-1}, \ldots , \xi _{t+r-1})=\pi \right) - p(\pi )\Big )\overset{\mathcal {D}[0,1]}{\Longrightarrow }\ \sigma _\pi B(\tau ), \quad \tau \in [0,1], \end{aligned}$$

with variance

$$\begin{aligned} \sigma _\pi ^2&= \mathop {\operatorname {Var}}\left( \mathbbm {1} \left( \Pi (\xi _{0}, \ldots , \xi _{r})=\pi \right) \right) \\&\quad +2\sum _{j=1}^\infty \mathop {\operatorname {Cov}}\left( \mathbbm {1} \left( \Pi (\xi _{0}, \ldots , \xi _{r})=\pi \right) , \mathbbm {1} \left( \Pi (\xi _{j}, \ldots , \xi _{j+r})=\pi \right) \right) . \end{aligned}$$

For $\tau =1$, we obtain

17 $$\begin{aligned} \sqrt{n}\Big (\hat{p}_n(\pi ) - p(\pi )\Big )\xrightarrow {\mathcal {D}} \mathcal {N}(0,\sigma _\pi ^2). \end{aligned}$$

#### Proof of Theorem 3

As a consequence of Lemma 1 and relation (13), the proof applies Theorem 1 to $({\textbf {V}}_\pi {\textbf {X}}_t)_{t\ge 1}.$ By assumption, $b_0\ne 0.$ In (6), we have already verified Assumption 1 for the linear process $({\textbf {X}}_t)_t$ with ${\textbf {X}}_t=\left( X_t, \ldots , X_{t+r-1} \right) ^\top .$ Since ${\textbf {V}}_\pi$ is invertible, Assumption 1 also holds for the linear process $({\textbf {V}}_\pi {\textbf {X}}_t)_{t\ge 1}.$ All the assumptions needed for Theorem 1 are satisfied, concluding the proof. $\square$

We now derive the limit distribution of the estimator for ordinal pattern probabilities for a class of processes whose increments exhibit long-range dependence.

#### Theorem 4

(Long-Range Dependence) Let $(\xi _t)_{t\ge 0}$ be a time series whose increments $X_t=\xi _t-\xi _{t-1}$ form a linear process $X_t=\sum _{j=0}^\infty b_jZ_{t-j}$ with $b_j \overset{j\rightarrow \infty }{\sim }\ j^{d-1}$ for $d\in (0,1/2)$. If $Z_1$ admits a density *f* such that $f\in L^{\infty }(\mathbb {R})\cap C^1(\mathbb {R})$ and $f' \in L^\infty (\mathbb {R})$, with finite fourth moment $\mathbb {E}[|Z_1|^4] < \infty$, then

$$\begin{aligned} n^{ \frac{1}{2}-d} \left( \hat{p}_n(\pi ) - p(\pi ) \right) \overset{\mathcal {D}}{\longrightarrow }\ \mathcal {N}(0, \sigma _\pi ^2), \end{aligned}$$

where $\sigma _\pi ^2 = C_d (\nabla \tilde{p}({\textbf {0}}))^{\top } {\textbf {V}}_\pi {\textbf {E}} {\textbf {V}}_\pi ^{\top } \nabla \tilde{p}({\textbf {0}})$, with $C_d = \frac{\Gamma (d)^2 }{\Gamma (2d+2)\cos (\pi d)}$, $\tilde{p}(\cdot ) := \mathbb {P}({\textbf {V}}_\pi {\textbf {X}}_1 \le \cdot )$, and ${\textbf {E}}$ the $r \times r$ matrix with all entries equal to 1.

Since ${\textbf {E}}=\varvec{1}\varvec{1}^\top$ with $\varvec{1}=(1,\ldots ,1)^\top ,$ we can also write $((\nabla \tilde{p}({\textbf {0}}))^{\top } {\textbf {V}}_\pi {\textbf {E}} {\textbf {V}}_\pi ^{\top } \nabla \tilde{p}({\textbf {0}})=(\nabla \tilde{p}({\textbf {0}}))^{\top } {\textbf {V}}_\pi \varvec{1})^2.$

#### Proof of Theorem 4

We apply Theorem 2. Since $b_j\sim j^{d-1}$ and $({\textbf {Z}}_{j})_{j \in \mathbb {Z}}$ forms an i.i.d. sequence with variance ${\textbf {E}}$, Lemma 1 shows that the multivariate linear process ${\textbf {X}}_t = (X_t, \ldots , X_{t+r-1})^\top =\sum _{j=0}^\infty {\textbf {B}}_j {\textbf {Z}}_{t-j}$ satisfies all the assumptions of Theorem 2, and it inherits the decay characteristics in the sense that ${\textbf {B}}_j \sim j^{d-1} {\textbf {I}}_r$ as $j \rightarrow \infty ,$ and ${\textbf {V}}_\pi {\textbf {I}}_r$ is invertible. Moreover, in Proposition 9, we show that in this case (C18) is satisfied, such that Theorem 2 can be applied for $\left( {\textbf {V}}_\pi {\textbf {X}}_t\right) _{t\ge 1}$ and ${\textbf {u}}={\textbf {0}}$, leading to

$$\begin{aligned} n^{1/2-d}( \hat{p}(\pi )-p(\pi ))&= n^{1/2-d}\Big ( \frac{1}{n}\sum _{t=1}^n \mathbbm {1}({\textbf {V}}_\pi {\textbf {X}}_t \le {\textbf {0}}) -\tilde{p}({\textbf {0}})\Big ) \\&\xrightarrow {\mathcal {D}} \mathcal {N}\bigg (0,\frac{\Gamma (d)^2 }{\Gamma (2d+2)\cos (\pi d)}( \nabla \tilde{p}({\textbf {0}}))^{\top } {\textbf {V}}_\pi {\textbf {E}} {\textbf {V}}_\pi ^\top \nabla \tilde{p}({\textbf {0}}) \bigg ). \end{aligned}$$

$$\square $$

We conclude this section by providing two examples. In the first example, the underlying time series $(\xi _t)_{t\ge 0}$ exhibits long-range dependence but the increment process $(X_t)_{t\ge 1}$ is short-range dependent. This allows us then to get the parametric $\sqrt{n}$ convergence rate. The second example shows that Theorem 4 applies to a class of FARIMA processes.

#### Example 1

(Short-range dependent increments) Let $d\in (0,1/2)$ and consider a linear process $\xi _t = \sum _{j=0}^\infty a_j Z_{t-j}$ with $a_j\sim j^{d-1}$ with $a_0\ne 0$ and $Z_1$ admitting a continuous and bounded probability density function. We now show that the increment process $X_t=\xi _t-\xi _{t-1}$ is a short-range dependent linear process. Indeed, it holds that

$$\begin{aligned} X_t = \sum _{j=0}^\infty b_j Z_{t-j} \quad \text {with}\quad b_j = {\left\{ \begin{array}{ll} a_j- a_{j-1}, & j\ge 1 \\ a_0\ne 0, & j=0\\ \end{array}\right. }. \end{aligned}$$

Moreover, $a_j \sim j^{d-1}$ as $j\rightarrow \infty$ and the mean value theorem imply $b_j \sim (d-1)j^{d-2}$ as $j\rightarrow \infty .$ Because of $d\in (0,1/2),$ $d-2 \in (-2,-3/2)$ and $(b_j)_{j \in \mathbb {N}}$ is summable. Since $Z_1$ has continuous and bounded probability density function, Theorem 3 yields for any ordinal pattern $\pi$,

$$\begin{aligned} \sqrt{n}\left( \hat{p}_n(\pi ) - p(\pi ) \right) \xrightarrow {\mathcal {D}} \mathcal {N}\left( 0, \sigma _\pi ^2\right) , \end{aligned}$$

with $\sigma _\pi ^2$ defined as in Theorem 3.

#### Example 2

(Long-range dependent increments) We consider a FARIMA$(p,d,q)$ process with $d < 1/2$, defined by $\phi (B)X_t = \theta (B)(I-B)^{-d}Z_t$, where $B^jX_t = X_{t-j}$. As shown by Pipiras and Taqqu (2017), if $\phi (z)$ and $\theta (z)$ have no common roots, and $\phi (z)$ has no zeros on the unit circle, $(X_t)_{t \ge 1}$ exhibits long-range dependence and can be expressed as:

$$\begin{aligned} X_t = \sum _{j=0}^\infty b_j Z_{t-j}, \quad b_j \sim \frac{\theta (1)}{\phi (1)}\frac{j^{d-1}}{\Gamma (d)}. \end{aligned}$$

Then, the processes $\xi _t=\sum _{j=1}^t X_j$ satisfies the conditions of Theorem 4.

#### Example 3

(Long-range dependent increments) Consider the linear process

$$\begin{aligned} X_t:= \sum _{j=1}^\infty a_j Z_{t-j}, \quad \text {with}\quad a_j = j^{d-1}, \quad 0< d < \tfrac{1}{2}, \end{aligned}$$

with $Z_j \overset{\text {i.i.d.}}{\sim } f$ and $f$ the logistic density

$$\begin{aligned} f(z) = \frac{e^{-z}}{(1 + e^{-z})^2}, \quad z \in \mathbb {R}. \end{aligned}$$

The density $f$ is bounded and admits the bounded derivative $f'(z) = -\frac{e^{-z}(1 - e^{-z})}{(1 + e^{-z})^3}$. Lastly, $f(z) \sim e^{-|z|}$ as $|z| \rightarrow \infty$ implies $\mathbb {E}[Z_t^4] < \infty$.

We conclude this section by referring to the survey by Taqqu et al. (1995) for an overview of existing methods for estimating the long-range dependence parameter $d$. Ordinal pattern-based estimators of $d$ can be found in Betken et al. (2021) under long-range dependence (and Sinn and Keller 2011 for ranges of *d* ensuring short-range dependece). As an alternative to direct estimation of $d$, we also highlight resampling-based approaches. In particular, subsampling methods under long-range dependence are discussed in Betken and Wendler (2018), while Shao and Wang (2013) provide a comprehensive account of self-normalized limit theorems.

### Some related works

Empirical processes of linear models have been widely explored in the literature. Notable contributions include the work of Ho and Hsing (1996), who developed asymptotic expansions for the empirical process of long-range dependent linear processes, and Giraitis and Surgailis (1999), who established functional non-central limit theorems for linear processes with long-range dependence. These results, which rely on the reduction principle, were further extended by Wu (2003).

Limiting theorems for empirical processes in linear time series with short-range dependence have been studied by various authors. Furmańczyk (2007) presented a central limit theorem for $g({\textbf {X}}_j)$, where $({\textbf {X}}_t)_{t\ge 1}$ is a multivariate linear process. Under mild conditions on the subordinated function $g$ and the finite second moment of the innovations ${\textbf {Z}}_1$, Furmańczyk concluded that $\frac{1}{\sqrt{n}}\sum _{j=1}^{\lfloor n\tau \rfloor } g({\textbf {X}}_j)\Longrightarrow (B(\tau ))_{\tau \in [0,1]}$. A similar setting was discussed in Wu (2002), who expanded on the results of Ho and Hsing (1997). In their Theorem 4.1, Ho and Hsing presented a central limit theorem for univariate linear processes with short-range dependence, imposing technical conditions and convergence similar to those in our Theorem 3, specifically the condition $\sum _j |a_j|<\infty$, where $a_j$ are the coefficients of the process. Wu (2002) obtained the same result under less stringent conditions. Additionally, in his Theorem 4, he derived the limiting distribution for empirical processes with the indicator function $g(x_1, \ldots , x_p)=\mathbbm {1}_{\{x_1\le s_1, \ldots , x_p\le s_p \}} - \mathbb {P}( X_1\le s_1, \ldots , X_p\le s_p )$, which directly applies to (13). Wu assumed that the characteristic function $\phi _Z$ of $Z_1$ satisfies $\int |\phi _Z(t)|^r\,dt < \infty$ for some $r \in \mathbb {N}$. He also defined $A_k(\delta ) := \sum _{t=k}^{\infty } |a_t|^\delta$, and required that $\sum _{n=1}^{\infty } \sqrt{A_n(\delta )/n} < \infty$ to derive (17). With the additional assumption $A_n(\delta ) = \mathcal {O}(n^{-q})$ for some $q > 1$, he further derived (16). While Wu’s approach imposes less stringent conditions on the innovations of the process (allowing, for example, discrete innovations), the only assumption we impose on the coefficients is $\sum _j |a_j|<\infty$, which is weaker than Wu’s condition $\sum _{n=1}^{\infty } \sqrt{A_n(\delta )/n} < \infty$. Indeed, Wu’s Lemma 1 shows $\sum _j|a_j|<\sum _{n=1}^{\infty } \sqrt{A_n(\delta )/n}$. For instance, the imposed conditions in this paper are suitable for processes with summable coefficients and innovations with $L^1$ characteristic functions, which, by the well-known inversion formula, would admit continuous and bounded densities, thereby fulfilling our conditions. Conversely, there are examples where Wu’s conditions hold, such as when the process is $m$-dependent and the innovations are sufficiently regular. Another example are uniform densities, which are not continuous but still satisfy Wu’s conditions.

For the case of short-range dependence, Schnurr and Dehling (2017) establish the asymptotic distribution of the ordinal patterns estimator $$\hat{p}_n(\pi )$$ for 1-approximating functionals of the absolutely regular process $$(Z_j)_{j\in \mathbb {N}}$$. They assume that $$(X_t)_{t \ge 1}$$ is a 1-approximating functional of $$(Z_{j})_{j \in \mathbb {N}}$$, with summable mixing coefficients $$(\beta _j)_{j \in \mathbb {N}}$$ (i.e., $$\sum _{j} \beta _j < \infty $$), indicating that $$(X_t)_{t\ge 1}$$ exhibits short-range dependence. Furthermore, if the 1-approximating coefficients $$(k_j)_{j \in \mathbb {N}}$$ of $$(X_t)_{t\ge 1}$$ are such that $$\sum _{j} \sqrt{k_j} < \infty $$, and the distributions of $$X_i - X_1$$ are Lipschitz continuous for $$i \in \{1, \ldots , r\}$$ then, (17) holds. However, their result does not cover the full spectrum of short-range dependent processes. For instance, consider $$X_t= \sum _{j=0} a_j Z_{t-j}$$ with $$a_0=1 $$ and $$a_n = \frac{1}{n^{\alpha }}$$. For $$\alpha >1$$, $$(a_j)_{j \in \mathbb {N}}\in \ell ^1 \subset \ell ^2$$. Suppose the innovations are i.i.d. and $$Z_1\sim f$$ where *f* is continuous and bounded. Under these conditions, the assumptions of Theorem 3 are satisfied for all $$\alpha >1$$; however, $$\sum _{j}\sqrt{k_j}<\infty $$ only holds for $$\alpha > 5/2.$$ To see this, applying their Lemma 1, the 1-approximating sequence $$(k_m)_{m \in \mathbb {N}}$$ for $$X_t$$, is given by $$k_m= \left( \sum _{n=m+1}^\infty a_n^2 \right) ^{1/2}.$$ Therefore,

$$\begin{aligned} k_m^2=\sum _{n=m+1}^\infty a_n^2 = \sum _{n=m+1}^\infty \frac{1}{n^{2\alpha }} \sim \int _{m}^\infty x^{-2\alpha }\, dx = \frac{1}{2\alpha -1} m^{1-2\alpha }. \end{aligned}$$

Thus, the condition $\sum _{m=1}^\infty \sqrt{k_m} < \infty$ is satisfied only for $1-2\alpha < -4$, that is, $\alpha > 5/2.$

Lastly, Beran and Telkmann (2017) demonstrated a reduction principle for multivariate empirical processes under long-range dependence. Their work provided a heuristic proof. Similar to their approach, and based on the martingale decomposition of Ho and Hsing, we reprove a point-wise multivariate reduction principle tailored to the ordinal patterns map. Importantly, our result does not hold uniformly but for each fixed point. This choice is motivated by the fact that, as discussed, the estimator for the ordinal pattern probabilities (13) is the empirical sum process evaluated at ${\textbf {0}}$. This simplification also allows us to eliminate the smoothness assumption on the innovations imposed by Beran and Telkmann (2017).

## Applications of ordinal patterns

Electroencephalography (EEG) is a noninvasive method widely utilized in medical and scientific research to measure and analyze the brain’s electrical activity. EEG records the electrical potentials generated by neural activity through electrodes placed on the scalp. An application of EEG is the identification and characterization of sleep stages. According to the classical methodology of Kales and Rechtschaffen (1968), adult sleep is divided into six stages: wakefulness (W), stage 1 (S1), stage 2 (S2), stage 3 (S3), stage 4 (S4) and rapid eye movement (REM). Each stage corresponds to distinct patterns of brain activity observed in EEG recordings. The classification of sleep stages is based on the segmentation of EEG recordings into non-overlapping intervals, referred to as epochs (typically 30 s); see Kales and Rechtschaffen (1968). For each epoch, the corresponding sleep stage is determined by experts. For a comprehensive overview of techniques used to analyze EEG data, see Zhang et al. (2024) and Gonen and Tcheslavski (2012).

Ordinal patterns have recently been used for sleep stage classifications in EEG data. Sinn et al. (2013) classified sleep stages by computing the distribution of ordinal patterns in different parts of the time series and then detect the locations of breaks by the maximum mean discrepancy statistics of the sequence of ordinal pattern distributions. Another methodology that has been receiving great attention in the field is the analysis of EEG via permutation entropy (PeEn). Introduced by Bandt and Pompe (2002), PeEn is defined as the Shannon entropy of the distribution of ordinal patterns of a fixed length, i.e., as the quantity $\text {PeEn}(p(\pi ^1), \ldots , p(\pi ^{(r+1)!}))= -\sum _{i=1}^{r!} p(\pi ^i) \log ( p(\pi ^i))$, where $\{\pi ^1,\ldots , \pi ^{(r+1)!}\}=\mathcal {S}_r$ is the set of permutations of length $r+1$. The value of the PeEn is computed for each epoch, and considerable differences in value can be observed for different sleep stages. Thus, the complex dynamics of EEG is then measured by looking at the series of permutation entropies across non-overlapping epochs of EEG recordings. A more refined analysis of the permutation entropy is due to Berger et al. (2017). Their data analysis of the PeEn with $r=2$ in one stage of EEG data shows that, for $\hat{p}$ being the ordinal patterns estimator (13),

18 $$\begin{aligned} \mathbb {E}[\hat{p}(0,1,2)]\approx &  \mathbb {E}[\hat{p}(2,1,0)]\quad \text {and}\quad \mathbb {E}[\hat{p}(0,2,1)]\approx \mathbb {E}[\hat{p}(2,1,0)]\nonumber \\\approx &  \mathbb {E}[\hat{p}(1,2,0)]\approx \mathbb {E}[\hat{p}(1,0,2)]. \end{aligned}$$

On a population level, assuming stationary Gaussian observations is sufficient for equality, in the sense that if $(\xi _t)_{t\ge 0}$ is stationary Gaussian then

19 $$\begin{aligned} p(0, 1, 2)= &  p(2,1,0) \quad \text {and}\quad p(0,2,1)=p(2,1,0)=p(1,2,0)\nonumber \\= &  p(1,0,2). \end{aligned}$$

Thus, in order to study the dynamics of PeEn, we can study the set of patterns $\mathcal {T}=\{ (0,2,1),(2,1,0),(1,2,0),(1,0,2)\}.$ An element of this set occurs with probability $q:= p(0,2,1)+p(2,1,0)+p(1,2,0)+p(1,0,2)$. Likewise, the probability to observe the all-raising pattern (0, 1, 2) or the all-falling pattern (2, 1, 0) is $1-q$. It follows that the permutation entropy

$$\begin{aligned} \text {PeEn}=q\log \frac{4}{q} + (1-q) \log \frac{2}{1-q}\; \end{aligned}$$

only depends on *q*. The corresponding estimator of *q* for an epoch $\left( \xi _t, \ldots , \xi _{t+m+1}\right)$ of $m+2$ observations is

20 $$\begin{aligned} \hat{q}_m&:= \frac{1}{m} \sum _{i=0}^{m-1} \sum _{\gamma \in \mathcal {T}} \mathbbm {1} \left( \{\Pi (\xi _{t+i}, \xi _{t+i+1}, \xi _{t+i+2}) = \gamma \}\right) \end{aligned}$$

21 $$\begin{aligned}&=\hat{p}_m(0,2,1)+\hat{p}_m(2,1,0)+\hat{p}_m(1,2,0)+\hat{p}_m(1,0,2). \end{aligned}$$

**Fig. 2:**
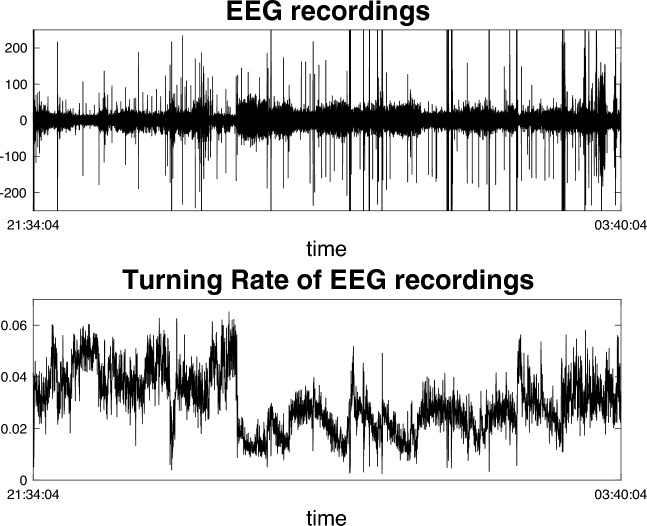
Top: EEG recordings of a healthy individual (4th patient from the CAP Sleep Database Terzano et al. 2002), originally sampled at 512 Hz, resulting in a time series of 19,553,792 data points. Due to the high resolution and length, detailed features are difficult to discern. Bottom: Corresponding turning rate series, where each data point represents an 8-s segment of the EEG recording. The sleep cycles are visible

The probability $q = \sum _{\gamma \in \mathcal {T}} p(\gamma )$ is called *turning rate* and the quantity $\hat{q}_m$ is called *turning rate estimator*; see Bandt (2017, 2020). Based on the turning rate, Bandt empirically identified different stages of sleep in EEG recordings that closely aligned with expert stage annotations. For this, Bandt partitions the EEG data recording into consecutive blocks of a length corresponding to 30 s, i.e., $(\xi _1,\ldots , \xi _{30\,s}), (\xi _{31\,s}, \ldots , \xi _{60\,s}),\ldots$ and computes the turning rate estimate for each block via (21). The corresponding turning rate series is plotted in Fig. 2, where the sleep cycles become immediately visible.

Kedem (1986) showed that the turning rate of a time series is closely related to the centroid of its power spectrum. Most notably, if $\xi _0, \ldots , \xi _{n+1}$ is a zero-mean stationary process, the turning rate is equivalent to the well-established zero-crossing estimator. For $( \xi _t)_{t \ge 0}$ a stationary zero-mean Gaussian time series, using Lemma 1 from Sinn and Keller (2011) and the results of Kedem (1986), the following can be shown

22 $$\begin{aligned} \cos {(\pi \mathbb {E}[\hat{q}_m])} = \rho (1) = \omega ^B, \end{aligned}$$

where $\rho (1)$ is the autocorrelation of $(X_t)_{t\ge 1}$ at lag 1 and $\omega ^B$ is a frequency of the spectrum of $(X_t)_{t \ge 1}$ known as the *spectral centroid* or *barycenter of the spectrum*. This relationship, expressed in equation (22), explains that a change in the autocorrelation $\rho (1)$ corresponds to a change in the frequency that dominates the spectrum. To illustrate this concept, Fig. 2 shows how the turning rate tracks the dominant frequencies over time. For example, when working with signals dominated by specific frequency bands (such as EEG signals characterized by alpha, beta waves, etc.), variations in the turning rate reveal shifts in the most prominent frequency components.

The analysis in Bandt (2017) emphasizes the visual inspection of turning rate plots across multiple channels, demonstrating that these plots exhibit significant overlap with the corresponding doctor-annotated sleep stage diagrams. Complementary to Bandt’s analysis, in the next section we rigorously evaluate the likelihood of transitions between stages with formal statistical confidence. Specifically, we introduce a hypothesis test designed to detect changes in the mean of the turning rate series. In the case of Gaussian distributions, a significant shift in the mean directly corresponds to a change in the parameter $\rho (1)$, signaling a transition to a different sleep stage.

### Change point detection via turning rate analysis

In this section, we address the problem of testing whether a given time series $\xi _0, \ldots , \xi _{n+1}$ exhibits stationarity in its increments $X_1, \ldots , X_{n+1}$ or whether there is a structural break in their distribution at some unknown point $1 \le k^*\le n+1$. The core idea of the test is to analyze and compare the ordinal pattern distributions across different segments of the time series. Under the null hypothesis, the stationarity of the increments ensures the stationarity of their ordinal patterns. Consequently, any observed change in the ordinal pattern distribution must reflect a change in the underlying time series distribution. This insight motivates the turning rate estimator as a test statistic. Unlike methods based on the autocovariance, which depend on the second-order properties of the time series, ordinal patterns rely on relative ordering. This causes the ordinal pattern distribution to be invariant under monotone transformations of the time series. That is, for any monotone function $G$, the transformed series $(G(\xi _t))_{t\ge 0}$ retains the same ordinal pattern structure as $(\xi _t)_{t\ge 0}$. This invariance is particularly useful in applications involving transformations such as logarithmic or power-law scaling. In contrast, monotone transformations generally alter the autocovariance structure, so autocovariance-based methods may need to be adapted accordingly.

If the increments $X_1, \ldots , X_{n+1}$ form a stationary sequence, the estimator (21) can be expressed in terms of ${\textbf {X}}_t=(X_t, X_{t+1})^\top$ as

23 $$\begin{aligned} \hat{q}_n -q = \frac{1}{n}\sum _{t=0}^{n-1} h( {\textbf {X}}_t), \quad h({\textbf {X}}_t):= \sum _{\gamma \in \mathcal {T}} \mathbbm {1}\left( {\textbf {V}}_{\gamma }{\textbf {X}}_t \le {\textbf {0}} \right) -q, \end{aligned}$$

where the matrices ${\textbf {V}}_\gamma$ for $\gamma \in \mathcal {T}$ are

24 $$\begin{aligned}&{\textbf {V}}_{(0,2,1) }= \left( \begin{array}{cc} 0& 1 \\ -1 & -1 \end{array} \right) , \quad {\textbf {V}}_{ (2,0,1) }= \left( \begin{array}{cc} -1& -1 \\ 1& 0 \end{array} \right) , \nonumber \\&{\textbf {V}}_{(1,2,0) }= \left( \begin{array}{cc} -1& 0 \\ 1& 1 \end{array} \right) , \quad {\textbf {V}}_{(1,0,2) }= \left( \begin{array}{cc} 1& 1 \\ 0& -1 \end{array} \right) . \end{aligned}$$

We will formalize the test in terms of ordinal patterns by identifying the change points in the corresponding turning rate series. Given time series data $\xi _0, \ldots , \xi _{n+1}$, the *turning rate series* is defined as the collection of $n_b = \left\lfloor \frac{n+2}{m+2} \right\rfloor$ random variables $\hat{q}_{1,m}, \ldots , \hat{q}_{n_b,m}$, where each $\hat{q}_{j,m}$ represents the turning rate (relation (21)) computed over non-overlapping, consecutive blocks of length $m+2$ extracted from $\xi _0, \ldots , \xi _{n+1}$. Formally, $\hat{q}_{j,m}$ is defined by

25 $$\begin{aligned} \hat{q}_{j,m}&= \frac{1}{m} \sum _{i=0}^{m-1} \sum _{\gamma \in \mathcal {T}} \mathbbm {1} \Big ( \Pi \big (\xi _{(j-1)(m+2)+i}, \xi _{(j-1) (m+2)+i+1}, \xi _{(j-1)(m+2)+i+2}\big ) = \gamma \Big ), \nonumber \\&\quad \text {for}\quad j = 1, \ldots , n_b. \end{aligned}$$

We consider the following test problem:

26 $$\begin{aligned} \mathcal {H}_0:\, \mathbb {E}[\hat{q}_{1,m}]&=\cdots =\mathbb {E}[\hat{q}_{n_b,m}] \quad \text {vs} \nonumber \\ \mathcal {H}_1: \, \mathbb {E}[\hat{q}_{1,m}]&=\cdots =\mathbb {E}[\hat{q}_{\lfloor n_b \tau ^\star \rfloor , m}]\ne \mathbb {E}[\hat{q}_{\lfloor n_b \tau ^\star \rfloor +1,m}]\nonumber \\&=\cdots =\mathbb {E}[\hat{q}_{ n_b,m}]\quad \text {for some}\quad \tau ^\star \in [0,1]. \end{aligned}$$

The test problem (26) is framed akin to a conventional mean change point detection problem. For this purpose, we can employ the CUSUM statistic

27 $$\begin{aligned} \max \limits _{k=1, \ldots , n_b -1}\left| \sum \limits _{j=1}^{k}\hat{q}_{j,m}-\frac{k}{n_b}\sum \limits _{j=1}^{n_b}\hat{q}_{j,m}\right| , \end{aligned}$$

as our test statistic. The asymptotic distribution of (27) is typically obtained through an application of the continuous mapping theorem to

28 $$\begin{aligned} \frac{m}{\sqrt{n}} \sum \limits _{j=1}^{\left\lfloor n_b \tau \right\rfloor }(\hat{q}_{j,m} -q), \quad \text {as} \quad n\rightarrow \infty , \end{aligned}$$

where $\tau \in [0,1]$. The asymptotic distribution of (28) derives from the fact that

$$\begin{aligned} \frac{m}{\sqrt{n}} \sum \limits _{j=1}^{\left\lfloor n_b \tau \right\rfloor }(\hat{q}_{j,m} -q) = \frac{1}{\sqrt{n}}\sum _{t=1}^{\lfloor n \tau \rfloor } h({\textbf {X}}_t)+o_\mathbb {P}(1), \end{aligned}$$

where the convergence of the partial sum on the right-hand side is given in Lemma 10. The remainder on the right-hand side of the above equation adjusts for those summands $h({\textbf {X}}_t)$ that due to aggregation of the data in the computation of the turning rate do not enter the sum on the left-hand side. Since the number of these summands is of order $n/m=o(\sqrt{n})$ and, by assumption, $m/\sqrt{n}\longrightarrow \infty$, the remainder term is (due to standardization by $\sqrt{n}$) asymptotically negligible. For a detailed proof, see Theorem 11 in Appendix D. Furthermore, the condition $m \gg \sqrt{n}$ permits the block size to grow slowly with $n$, e.g., $m = n^{1/2 + \delta }$ for some $\delta > 0$, without affecting the asymptotic distribution. Aggregating observations over such blocks smooths out high resolution. This not only preserves major structural changes for statistical inference but also enhances interpretability and visualization, making finer deviations more perceptible in plots compared to working directly with all $n$ observations. Additionally, blocking reduces the variance of empirical estimators by averaging out local fluctuations.

The asymptotic limit of the CUSUM statistics is the content of the next theorem.

#### Theorem 5

Let $\xi _0, \ldots , \xi _{n+1}$ be a time series whose increments $X_1,\ldots , X_{n+1}$ form a linear process $X_t=\sum _{j=0}^\infty a_j Z_{t-j}$ with $\sum _{j=0}^\infty |a_j|< \infty$ and $Z_1$ admitting a continuous and bounded density and finite second moment $\mathbb {E}[|Z_1|^2]<\infty$. Consider the turning rate generated by blocks of size $m+2,$ and corresponding number of blocks $n_b=\lfloor (n+2)/(m+2)\rfloor$. If $m/\sqrt{n}\rightarrow \infty$, then

$$\begin{aligned} \frac{m}{\sqrt{n}}\max _{k=1, \ldots , n_b-1} \left| \sum _{j=1}^k \hat{q}_{j,m} - \frac{k}{n_b} \sum _{j=1}^{n_b} \hat{q}_{j,m} \right| \xrightarrow {\mathcal {D}} \sigma \sup _{\tau \in [0,1]} \left| B(\tau ) - \tau B(1) \right| ,\,\, \text {as}\,\, n\rightarrow \infty , \end{aligned}$$

with variance

29 $$\begin{aligned} \sigma ^2 =&\mathop {\operatorname {Var}}\left( \sum _{\gamma \in \mathcal {T}} \mathbbm {1}\left( \Pi (\xi _0,\xi _1,\xi _2)=\gamma \right) \right) \nonumber \\ &+2 \sum _{j=1}^\infty \mathop {\operatorname {Cov}}\left( \sum _{\gamma \in \mathcal {T}} \mathbbm {1}\left( \Pi (\xi _0,\xi _1,\xi _2)=\gamma \right) , \sum _{\gamma \in \mathcal {T}} \mathbbm {1}\left( \Pi (\xi _{j},\xi _{j+1},\xi _{j+2})=\gamma \right) \right) . \end{aligned}$$

#### Remark 3

If $X_1, \ldots , X_{n+1}$ forms a Gaussian time series, test (26) becomes equivalent to testing for a change in the autocorrelation parameter $\rho (1)$, as shown in (22). In this context, a significant shift in the mean of the turning rate series directly corresponds to a change in $\rho (1)$. For EEG time series, such a shift is indicative of a transition between different sleep stages.

### Variance estimation

The distribution of the stochastic limit $\sup _{\tau \in [0,1]} |B(\tau ) - \tau B(1)|$ can be approximated through Monte Carlo simulations. However, the long-run variance in (29) is generally unknown and requires estimation. A standard approach to estimating $\sigma ^2$ involves kernel-based methods. However, these methods are sensitive to the choice of the kernel bandwidth, with data-dependent bandwidth selection often leading to non-monotonic statistical power, as shown in Vogelsang (1998) and Crainiceanu and Vogelsang (2007). To address these limitations, we estimate $\sigma ^2$ using the self-normalization technique introduced by Shao and Zhang (2010) and Shao (2010); see also Betken (2016).

Taking the possibility of a structural change at time *k* into consideration, a normalization for the two-sample CUSUM statistic is obtained by combining the values of empirical variances computed with respect to the separate samples $\hat{q}_{1,m}, \ldots , \hat{q}_{k,m}$ and $\hat{q}_{k+1,m}, \ldots , \hat{q}_{n_b,m}$. Accordingly, we define

30 $$\begin{aligned} V^2_{k, n_b}:=\frac{1}{n_b}\sum _{t=1}^k S_t^2(1,k)+\frac{1}{n_b}\sum _{t=k+1}^n S_t^2(k+1,n_b) \end{aligned}$$

with

$$\begin{aligned} S_{t}(j, k):=\sum _{h=j}^t\left( \hat{q}_{h,m}-\bar{q}_{j, k}\right) , \quad \bar{q}_{j, k}:=\frac{1}{k-j+1}\sum _{t=j}^k\hat{q}_{t,m}, \end{aligned}$$

as normalizing sequence and we define the self-normalized CUSUM statistic by

31 $$\begin{aligned} SC_{n_b}:=\max _{1\le k\le n_b-1}\frac{\left| \sum _{j=1}^k\hat{q}_{j,m}-\frac{k}{n}\sum _{j=1}^{n_b}\hat{q}_{j,m}\right| }{V_{k,n_b}^2}. \end{aligned}$$

For testing the hypothesis of a change in the level of the turning rate series on the basis of the self-normalized CUSUM statistic $SC_{n_b}$, we need to set critical values for a corresponding hypothesis test. For this purpose, we establish the asymptotic distribution of the statistic as a corollary of Theorem 3:

#### Corollary 6

Assume that $\xi _0, \ldots , \xi _{n+1}$ satisfies the assumptions of Theorem 5. Consider the turning rate series generated by blocks of size $m+2$ and $n_b$ blocks, denoted by $\hat{q}_{1,m}, \ldots , \hat{q}_{n_b,m}$. If $m/\sqrt{n}\rightarrow \infty$, then as $n\rightarrow \infty$

32 $$\begin{aligned} SC_{n_b} \overset{\mathcal {D}}{\longrightarrow }\ \sup _{\tau \in [0,1]} \frac{|B(\tau )-\tau B(1)|}{ \left[ \int _0^\tau \left( B(s) - \frac{s}{ \tau } B(\tau ) \right) ^2 \, ds+ \int _{\tau }^1 \left( B(s) - B(\tau ) -\frac{s-\tau }{1-\tau } \left( B(1)-B(\tau ) \right) \right) ^2 \, ds\right] ^{1/2}}. \end{aligned}$$

The proof can be found in Appendix D.

## Simulation studies

We complement the theoretical results with simulation studies and an application to real data. We illustrate Corollary 6 using two simulated MA(1) time series: one without a change point and one where the moving average parameter changes, corresponding to a shift at lag 1 of the autocorrelation. Further, we examine the power of the test on simulated AR(1) time series with lengths of 500, 1000, and 2000, introducing breaks at 1/4, 1/3 and 1/2 of the data under various non-normally distributed innovations. Finally, we apply the test to EEG data to detect transitions between two sleep stages.

### Example 4

**Fig. 3:**
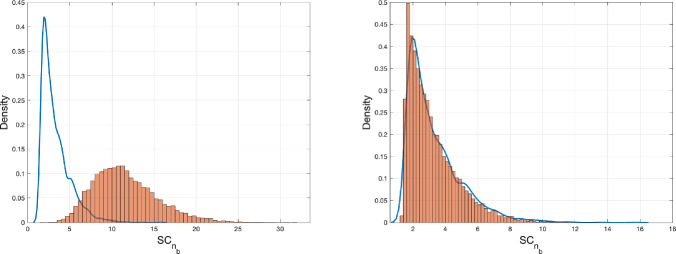
Histogram of $SC_{n_b}$ for $n=5000$ and 1000 simulations of MA(1) with (left) and without (right) change of $\rho (1)$. In the left plot, the autoregressive parameter changes from $\theta =0.4$ to $\theta =0.7$ after 50% of the observations. Under the null hypothesis the estimated 0.95 quantile is 6.335

(Setting A) We simulated two MA(1) time series, defined as $X_t = Z_t + \theta Z_{t-1}$, each consisting of 5000 data points with normally distributed innovations (see Fig. 3). For the first series, the MA parameter $\theta$ is set to $0.4$. In the second time series, $\theta$ changes from 0.4 to 0.7 after 2500 observations. The value $SC_{n_b}$ is computed for both series. Fig. 3 shows the distribution of the test statistics based on 1000 repetitions.

### Example 5

**Fig. 4:**
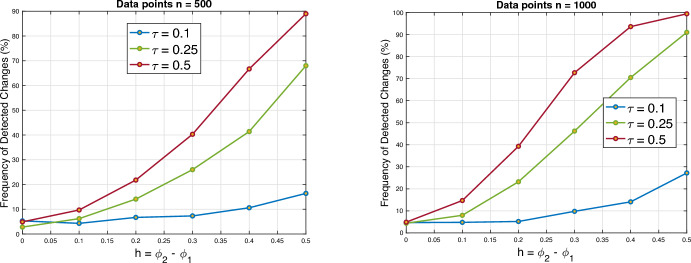
Power of the test for different values of $h=\phi _2-\phi _1$ with $n=500$ and $n=1000$ data points and Laplace distributed innovations. The curves correspond to different values of $\tau$, where changes occur at 1/10 for the blue curve $(\tau =0.1)$, at 1/4 for the green curve $(\tau =0.25)$ and at 1/2 for the red curve $(\tau =0.5)$. $\phi _1$ is set to 0.4. For $h=0$ (no change), the power corresponds to the significance level 5%

(Setting B) We simulate an AR(1) process $(X_t)_{t\ge 1}$ of the form

$$\begin{aligned} X_t = {\left\{ \begin{array}{ll} \phi _1 X_{t-1} + Z_t& t=1, \ldots ,\lfloor (n+1)\tau \rfloor ,\\ \phi _2 X_{t-1} + Z_t& t=\lfloor (n+1) \tau \rfloor \ +1, \ldots , n+2 \end{array}\right. } \end{aligned}$$

and set $\xi _t=\sum _{i=1}^t X_i$. For $h=\phi _2-\phi _1$ and $\phi _1=0.4$, Fig. 4 provides the frequency of detected changes for the values $h\in \{0.1,0.2,0.3,0.4,0.5\}$. Further, for fixed $h=0.4$, we consider time series of lengths 500, 1000 and 2000 data points. We analyze breaks after a fraction $\tau \in \{ 1/10, 1/4, 1/2 \}$ of the data for different innovation distributions. The frequency of detected changes are summarized in Table 1.

**Table 1:** Frequencies of detected changes in an AR(1) time series and for $h=\phi _2-\phi _1=0.4$

$n=500$				$n=1000$				$n=2000$			
Break	$\mathcal {N}(0,1)$	$t_2$	Lap (0, 4)	Break	$\mathcal {N}(0,1)$	$t_2$	Lap (0, 4)	Break	$\mathcal {N}(0,1)$	$t_2$	Lap (0, 4)
50	10.2	11.4	7.8	100	13.5	16.1	13.6	200	29.4	31	30.5
125	40	52.3	46	250	69	75	69.2	500	91.6	95.1	91.2
250	70	74.8	73.2	500	93.2	94.8	94.3	1000	99.8	99.7	99.5

### Example 6

**Fig. 5:**
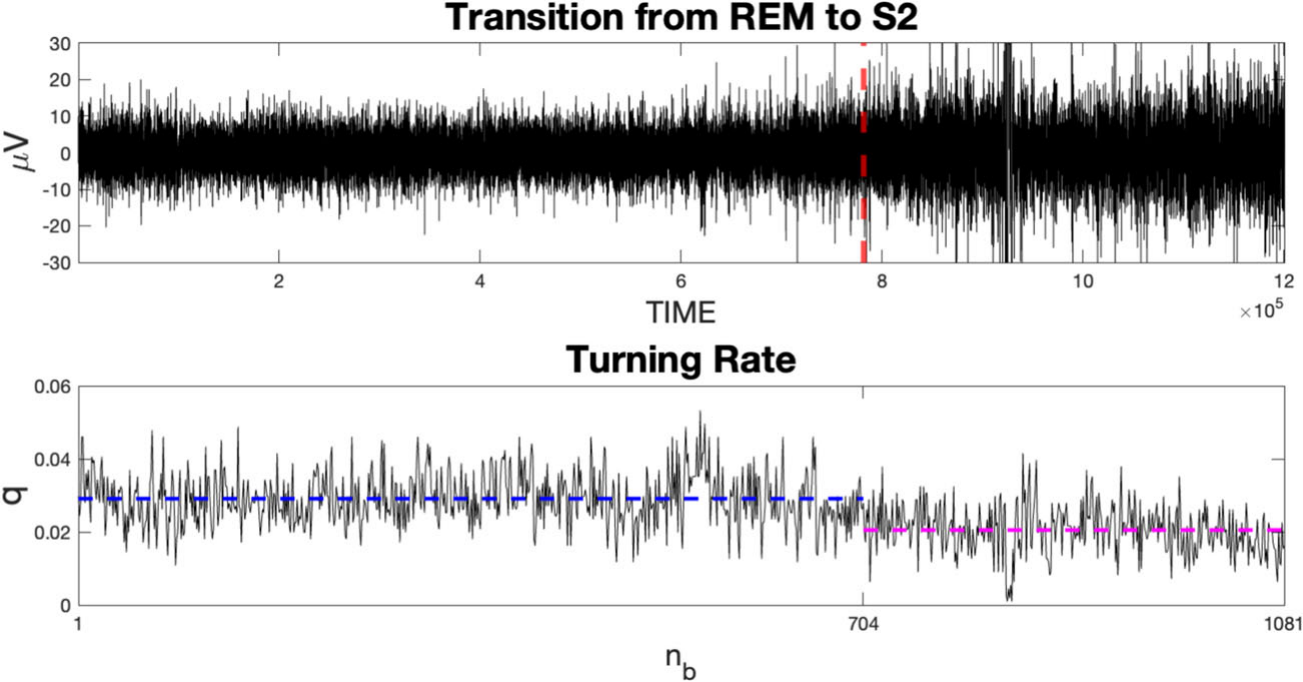
Extract of EEG recordings for the 5-th patient of the dataset (Terzano et al. 2002). The recordings cover approximately 30 min of observations extracted from the C4–P4 channel, in the temporal window going from 01:08:2 (REM) to 01:47:33 (S2). The time series contains $1.2\times 10^6$ data points

(Real Data) The upper plot of Fig. 5 depicts a segment of EEG recordings from a single patient sourced from the dataset by Terzano et al. (2002). The time series comprises data points sampled at 512 Hz over a 39-min interval. In this dataset, each 30-s batch is manually labeled with the corresponding sleep stage by an expert. In the depicted segment, the patient transitions from the REM phase to the S2 phase. Our objective is to employ the proposed method to statistically validate this transition and potentially pinpoint the exact time of occurrence.

The lower part of Fig. 5 shows the change in the corresponding turning rate series (dashed red line in the upper figure). Assuming the EEG to be Gaussian and stationary under the null hypothesis of no change, we reject the null hypothesis at significance level 0.05 and with a *p*-value of $7.29\times 10^{-5}.$ The same test applied to the corresponding two subsamples (from the beginning to the change point and from the change point to the end) fails to reject the null hypothesis with *p*-values 0.986 and 0.138, respectively. The source code for the simulations is available on GitHub at https://github.com/george24GM/Turning-rate-time-series.git

## Discussion and outlook

The main theoretical contribution of this work corresponds to the establishment of central limit theorems for relative frequencies in linear processes; see Sect. 2.1. An intriguing question is whether one can extend these limit theorems to estimators of the form $\tfrac{1}{n}\sum _{t=1}^n \mathbbm {1}({\textbf {X}}_t\in A)$ for more general sets *A*. In machine learning parlance, an ordinal pattern is a feature. While in the presented framework the ordinal pattern is fixed beforehand, machine learning learns the features from data. In view of the application to EEG data, one might design methods in future work that also select a suitable linear combination of the most relevant ordinal patterns from either supervised or unsupervised data. In view of the central limit theorems for relative frequencies presented in Sect. 2.1, one might also want to directly learn features $\mathbbm {1}({\textbf {W}}{\textbf {X}}_t\le {\textbf {v}})$ for a learnable weight matrix ${\textbf {W}}$ and a shift vector ${\textbf {v}}.$ The problem is then closely connected to training of neural networks with Heaviside activation function $\sigma (x)=\mathbbm {1}(x\ge 0)$.

## Notation Appendix

The Euclidean norm in $\mathbb {R}^r$ is denoted by $\Vert \cdot \Vert$. For $i_1, \ldots , i_h \ge 1$, let $\partial ^h_{i_1, \ldots , i_h} f$ denote the partial derivative of $f$ taken $h$ consecutive times with respect to the variables $i_1, \ldots , i_h$. We denote with $\nabla f$ the transposed gradient of *f*, given by $\nabla f ({\textbf {x}}) = \left( \partial _1 f({\textbf {x}}), \ldots , \partial _p f({\textbf {x}}) \right) ,$ and with $\nabla ^2 f$ the corresponding Hessian matrix, expressed as $\nabla ^2 f({\textbf {x}}) = \left( \partial ^2_{ps} f({\textbf {x}}) \right) _{p,s}.$ The symbol $C$ denotes a generic numerical constant that may change upon each appearance.

We define the truncated linear process ${\textbf {X}}_{t,j}$ as ${\textbf {X}}_{t,j}:=\sum _{i=0}^j {\textbf {A}}_{i} {\textbf {Z}}_{t-i}$. Specifically, ${\textbf {X}}_{t,0} = {\textbf {A}}_0{\textbf {Z}}_t$, and ${\textbf {X}}_t = {\textbf {X}}_{t,\infty }$. Further, let ${\textbf {R}}_{t,j}= {\textbf {X}}_{t}-{\textbf {X}}_{t,j}=\sum _{i=j+1}^\infty {\textbf {A}}_{i} {\textbf {Z}}_{t-i}$. For fixed ${\textbf {u}}=(u_0, \ldots , u_{r-1})^\top$,

1 $$\begin{aligned} p_j({\textbf {u}})=\mathbb {P}( {\textbf {X}}_{t,j} \le {\textbf {u}}), \quad p({\textbf {u}}) = \mathbb {P}( {\textbf {X}}_1 \le {\textbf {u}}). \end{aligned}$$

$p_j$ only depends on *j* as $(X_{t,j})_{t\ge 1}$ forms a stationary process. Moreover, we define a function $g:\mathbb {R}^r \rightarrow \mathbb {R}$ as Lipschitz if, for all ${\textbf {x}}, {\textbf {y}}\in \mathbb {R}^r$, it holds that $|g({\textbf {x}}) - g({\textbf {y}})| \le L \Vert {\textbf {x}}- {\textbf {y}}\Vert$, with the smallest constant *L* denoted by $\operatorname {Lip}(g)$. Lastly, the symbol *C* denotes a generic constant and the cumulative distribution function of ${\textbf {Z}}_1$ is denoted by $\mathcal {G}(\cdot ).$

The proofs of Theorems 3 and 4 rely on the following martingale decomposition (consequence of Lemma 6). Let $\mathcal {F}_t=\sigma ({\textbf {Z}}_t, {\textbf {Z}}_{t-1}, \ldots )$ be the sigma field generated by the past values of ${\textbf {Z}}_t$. Then, it holds that

2 $$\begin{aligned} \mathbbm {1}\left( {\textbf {X}}_t \le {\textbf {u}} \right) - p({\textbf {u}}) = \sum _{j=0}^\infty U_{t,j}, \quad U_{t,j}&:= \mathbb {E}\big [\mathbbm {1}({\textbf {X}}_t\le {\textbf {u}}) \big | \mathcal {F}_{t-j}\big ]\nonumber \\&\quad - \mathbb {E}\big [\mathbbm {1}({\textbf {X}}_t\le {\textbf {u}}) \big | \mathcal {F}_{t-j-1}\big ]\; \nonumber \\&= p_{j}({\textbf {u}} -{\textbf {R}}_{t,j}) - p_{j+1}({\textbf {u}} -{\textbf {R}}_{t,j+1}). \end{aligned}$$

## Proofs under short-range dependence

### Lemma 3

Let ${\textbf {X}}_t= \sum _{j= 0 }^\infty {\textbf {A}}_{j} {\textbf {Z}}_{t-j}$ be a multivariate linear process. If Assumption 1 holds for $J\ge 0$, then $\sup _{j\ge J} \operatorname {Lip}(p_j)\le \operatorname {Lip}(p_{J}) <\infty .$

### Proof of Lemma 3

In order to prove the Lipschitzness of $p_j$, we use the following relation: for all $j\ge 0$

A3 $$\begin{aligned} p_{j+1}({\textbf {u}})= &  \mathbb {P} \left( {\textbf {X}}_{t,j+1} \le {\textbf {u}}\right) = \mathbb {P} \left( {\textbf {X}}_{t,j} \le - {\textbf {A}}_{j+1} {\textbf {Z}}_{t-j-1}+{\textbf {u}} \right) \nonumber \\= &  \int p_j ({\textbf {u}} - {\textbf {A}}_{j+1} {\textbf {t}} ) \, d\mathcal {G}({\textbf {t}}). \end{aligned}$$

If there exists $j\ge 0$ for which $\text {Lip}(p_j)< \infty$, then for any ${\textbf {u}},{\textbf {v}} \in \mathbb {R}^r$

$$\begin{aligned} |p_{j+1}({\textbf {u}}) - p_{j+1}({\textbf {v}})|&\le \int \left| p_j({\textbf {u}}- {\textbf {A}}_{j+1} {\textbf {t}} ) - p_j ({\textbf {v}}- {\textbf {A}}_{j+1} {\textbf {t}} ) \right| \, d\mathcal {G}({\textbf {t}})\\ &\le \text {Lip}(p_j) \Vert {\textbf {u}}-{\textbf {v}}\Vert ; \end{aligned}$$

thus, $\text {Lip}(p_{j+1})\le \text {Lip}(p_j).$ To conclude that $\sup _{j\ge J} \operatorname {Lip}(p_j)\le \operatorname {Lip}(p_{J}) <\infty ,$ it is enough to prove that $\text {Lip}(p_{J})< \infty .$ By definition the function $p_{J}$ is the cumulative distribution function of the variable ${\textbf {X}}_{t,J}$. By Assumption 1, $D{\textbf {X}}_{t,J}$ is a vector of random variables with bounded Lebesgue density for an invertible matrix *D*. Such probability density function is denoted by $f_{J}.$ Then, we can write, for ${\textbf {u}}=(u_0,\ldots , u_{r-1})$,

$$\begin{aligned} p_{J}({\textbf {u}})= \int _{-\infty }^{u_0} \cdots \int _{-\infty }^{u_{r-1}} f_{J}(t_0, \ldots , t_{r-1})\, dt_0,\ldots dt_{r-1}. \end{aligned}$$

For any ${\textbf {u}}=(u_0, \ldots , u_{r-1})^\top$ and ${\textbf {v}}=(v_0, \ldots , v_{r-1})^\top$ we have $\{ {\textbf {X}}_{t,J}\le {\textbf {u}}\}/ \{ {\textbf {X}}_{t,J} \le {\textbf {v}} \}\subset \bigcup _{i=0}^{r-1} \{ u_i \wedge v_i \le {\textbf {X}}_{t,J,i} \le u_i \vee v_i \}$ with ${\textbf {X}}_{t,J,i}$ the *i*th component of ${\textbf {X}}_{t,J}$. It follows that

$$\begin{aligned} \mathbb {P}({\textbf {X}}_{t,J}\le {\textbf {u}})- \mathbb {P}({\textbf {X}}_{t,J}\le {\textbf {v}})=p_{J}({\textbf {u}})- p_{J}({\textbf {v}}) \le \sum _{i=0}^{r-1} \int _{ u_i \wedge v_i }^{ u_i \vee v_i } f_{J}^{(i)}(t_i) \, dt_i, \end{aligned}$$

where $f_{J}^{(i)}$ is the marginal density obtained from the $i$-th entry of the vector ${\textbf {X}}_{t,J}$, denoted by ${\textbf {X}}_{t,J}^{(i)}.$ Let $e_i$ be the *i*th vector of the canonical basis of $\mathbb {R}^r$, and note that each entry ${\textbf {X}}_{t,J}^{(i)}$ is obtained as ${\textbf {X}}_{t,J}^{(i)} = e_i ^\top {\textbf {X}}_{t,J}.$ Furthermore, ${\textbf {X}}_{t,J}^{(i)}$ admits a bounded probability density function. In fact, by Assumption 1, there exist an invertible matrix *D* and i.i.d. random variables $Y_0,\ldots , Y_{r-1}$ admitting a bounded density, such that

$$\begin{aligned} {\textbf {X}}_{t,J}^{(i)} = e_i ^\top {\textbf {X}}_{t,J} = e_i^\top {\textbf {D}}^{-1} \left( \begin{array}{c} Y_1\\ \vdots \\ Y_r \end{array}\right) = \sum _{j=0}^{r-1} (e_i^\top \cdot {\textbf {D}}^{-1}_{(j)}) Y_j , \end{aligned}$$

and ${\textbf {D}}^{-1}_{(j)}$ denoting the *j*th column of ${\textbf {D}}^{-1}.$ The right-hand side of the previous expression is the linear combination of independent and identically distributed random variables with bounded probability density. From the convolution formula, the resulting density is also bounded (by a constant denoted as *C*). Therefore, for any $i = 0, \ldots , r-1$, we have $\sup _{x\in \mathbb {R}^r}f_{J}^{(i)}(x) \le C$ for a positive constant $C$, and every integral is upper bounded as follows (shown here for $i = 0$):

$$\begin{aligned} \int _{u_0 \wedge v_0}^{u_0 \vee v_0} \, \underbrace{\left( \, \int _{-\infty }^{\infty }\cdots \int _{-\infty }^{\infty } f_{\textbf {J}}({\textbf {u}}) \, dt_1 \ldots dt_{r-2} \right) }_{f_{J}^{(0)} (u_0)}d t_0 \le C \Vert {\textbf {u}} -{\textbf {v}} \Vert _{\infty }. \end{aligned}$$

$$\square $$

As we study functionals that are indicators and can be bounded by one, the next result shows that the conclusion of Lemma 3.4 of Furmańczyk (2007) still holds although the Lipschitz condition only applies to $p_j$ with $j\ge J.$

### Lemma 4

Let ${\textbf {X}}_t= \sum _{j= 0 }^\infty {\textbf {A}}_{j} {\textbf {Z}}_{t-j}$ be a multivariate linear process satisfying $\sum _{j=0}^\infty \Vert {\textbf {A}}_{j}\Vert < \infty$ and Assumption 1. Then, for $U_{t,j}$ defined in (2)

(i) $$\mathbb {E}[\, U_{t,0}^2\,] \le P({\textbf {X}}_1\le {\textbf {u}}),$$
(ii) $$\mathbb {E}[\, U_{t,j}^2\, ] \le C \Vert {\textbf {A}}_{j+1}\Vert ^2.$$

### Proof

(i) follows from expanding the square, resulting in

$$\begin{aligned}&\mathbb {E}\left[ \, ( \mathbbm {1}({\textbf {X}}_t\le {\textbf {u}}) - \mathbb {E}[\mathbbm {1}({\textbf {X}}_t\le {\textbf {u}})|\mathcal {F}_{t-1} ] )^2 \,\right] \\&\quad = P({\textbf {X}}_t\le {\textbf {u}})-\mathbb {E}\big [\mathbb {E}[\mathbbm {1}({\textbf {X}}_t\le {\textbf {u}})|\mathcal {F}_{t-1} ]^2\big ] \le P({\textbf {X}}_1\le {\textbf {u}}). \end{aligned}$$

To prove (ii), notice that $|U_{t,j}|\le 1=\Vert {\textbf {A}}_{j}\Vert ^{-1}\Vert {\textbf {A}}_{j}\Vert ,$ and thus, for $j\le J-1,$ $\mathbb {E}[ \, |U_{t,j}|^2 \, ]\le C_1 \Vert {\textbf {A}}_{j+1}\Vert ^2$ with $C_1= \max _{j=0,\ldots ,J-1} \Vert {\textbf {A}}_{j}\Vert ^{-2}.$ Since *J* is fixed, $C_1$ is a finite constant as Assumption 1 holds and $\Vert {\textbf {A}}_{j} \Vert \ne 0$ for $j=0,\ldots , J$. To treat the case $j\ge J,$ notice that by (2) $U_{t,j}=p_{j}({\textbf {u}} -{\textbf {R}}_{t,j}) - p_{j+1}({\textbf {u}} -{\textbf {R}}_{t,j+1})$. Given the independence between ${\textbf {A}}_{j+1} {\textbf {Z}}_{t-(j+1)}$ and ${\textbf {R}}_{t,j+1}$, we have $p_{j+1}({\textbf {u}} -{\textbf {R}}_{t,j+1})=\int p_j ({\textbf {u}}-{\textbf {R}}_{t,j+1} - {\textbf {t}}) \, dF_{1,j}({\textbf {t}})$, where $F_{j}$ is the distribution function of ${\textbf {A}}_{j+1} {\textbf {Z}}$. In Lemma 3, we have shown that $p_j$ is Lipschitz for $j\ge J$, with constant $\text {Lip}(p_j)\le \text {Lip}(p_{J})$. Thus, for $j\ge J$, it holds that

$$\begin{aligned} |U_{t,j}|&\le \int |p_{j}({\textbf {u}} -{\textbf {R}}_{t,j}) - p_{j+1}({\textbf {u}} -{\textbf {R}}_{t,j+1} -{\textbf {t}})|\, dF_{1,j}({\textbf {t}})\\&\le \text {Lip}(p_j) \int \Vert {\textbf {R}}_{t,j}-{\textbf {R}}_{t,j+1} - {\textbf {t}}\Vert \, dF_{1,j+1}({\textbf {t}})\\&\le \text {Lip}(p_{J}) \left( \Vert {\textbf {R}}_{t,j}-{\textbf {R}}_{t,j-1}\Vert + \int \Vert {\textbf {t}}\Vert \, dF_{1,j}({\textbf {t}}) \right) \\&\le \text {Lip}(p_{J}) \left( \Vert {\textbf {A}}_{j+1} {\textbf {Z}}_{t-j-1}\Vert + \mathbb {E}\left[ \,\Vert {\textbf {A}}_{j+1} {\textbf {Z}}_{t-j-1}\Vert \, \right] \right) \\&\le \text {Lip}(p_{J}) \Vert {\textbf {A}}_{j+1}\Vert \left( \Vert {\textbf {Z}}_{t-j-1}\Vert + \sqrt{\mathbb {E}\left[ \Vert {\textbf {Z}}_{t-j-1} \Vert ^2\right] }\right) . \end{aligned}$$

Taking expectations, it follows that $\mathbb {E}[\, U_{t,{j}}^2\, ] \le C_2 \Vert {\textbf {A}}_{j+1}\Vert ^2$ for a constant $C_2$ that is independent of $j\ge J.$ Thus, (ii) holds with $C=\max (C_1,C_2).$ $\square$

### Theorem 7

(Theorem 4.2 in Billingsley 1968) Let $B:=\left( B(\tau )\right) _{\tau \in [0,1]}$ be a standard Brownian motion on [0, 1]. Let $W=\left( W(\tau )\right) _{\tau \in [0,1]}$ be a $\mathcal {D}[0,1]$-valued stochastic process. If there exists a process $V_{un}$ such that for all $u\in \mathbb {N}$:

(i) $$V_{u,n} \overset{\mathcal {D}[0,1]}{\Longrightarrow }\ \sigma _u B$$ for a certain $$\sigma _u>0$$,
(ii) There exists finite the limit $\sigma ^2 =\lim _{u\rightarrow \infty } \sigma _u^2 >0,$
(iii) $$ \liminf _{u\rightarrow \infty }\limsup _{n\rightarrow \infty } \mathbb {P}\left( |V_{u,n}-W_n|\ge \varepsilon \right) =0\quad \text {for any }\varepsilon >0.$$

Then, it holds that

$$\begin{aligned} W_n \overset{\mathcal {D}[0,1]}{\Longrightarrow }\sigma B\quad \text {as }n\rightarrow \infty . \end{aligned}$$

### Proof of Theorem 1

We apply Theorem 7 (Theorem 4.2 of Billingsley). The vectorized linear form ${\textbf {X}}_t=(X_t, \ldots , X_{t+r-1})^\top$, represented as a linear process of the form ${\textbf {X}}_t = \sum _{j=0}^\infty {\textbf {A}}_{j} {\textbf {Z}}_{t-j}$, permits the martingale difference decomposition (2). We set

$$\begin{aligned} W_n:&=\frac{1}{\sqrt{n}}\sum _{t=1}^{\lfloor n \tau \rfloor } \sum _{j=0}^\infty U_{t,j}, \quad V_{u,n}:=\frac{1}{\sqrt{n}} \sum _{t=1}^{\lfloor n \tau \rfloor } \sum _{j=0}^{u-1} U_{t,j}, \\ H_{u,n}:&=\frac{1}{\sqrt{n}}\sum _{j=0}^{u-1} \left( \sum _{t=1}^j U_{t,j} - \sum _{t=\lfloor n \tau \rfloor +1}^{\lfloor n \tau \rfloor +j} U_{t,j}\right) , \end{aligned}$$

We will show that $V_{u,n}$ satisfies Theorem 7.

(i) By construction it holds that

A3 $$\begin{aligned} V_{u,n}= \frac{1}{\sqrt{n}} \sum _{t=1}^{\lfloor n \tau \rfloor } \sum _{j=0}^{u-1} U_{t+j,j}+H_{u,n}. \end{aligned}$$

The advantage of formulation (A3) is that for every fixed *u*, $\left( \sum _{j=0}^{u-1} U_{t+j,j}, \mathcal {F}_t \right) _t$ forms a stationary, ergodic and centered martingale difference sequence. In fact, by setting $M_t(u):=\sum _{j=0}^{u-1} U_{t+j,j}$, we have $\mathbb {E}[M_t(u)]<\infty$ and, for all $s\le t-1$

$$\begin{aligned} \mathbb {E}\left[ M_t(u)|\, \mathcal {F}_s \right]&= \mathbb {E}\left[ \sum _{j=0}^{u-1} U_{t+j,j} \Bigg |\, \mathcal {F}_s \right] = \sum _{j=0}^{u-1}\mathbb {E}\left[ U_{t+j,j} |\, \mathcal {F}_{s} \right] \\&= \sum _{j=0}^{u-1}\mathbb {E}\left[ \, \left( \mathbb {E}\left[ \mathbbm {1}({\textbf {X}}_{t+j}\le {\textbf {u}}) \Big | \mathcal {F}_t\right] -\mathbb {E}\left[ \mathbbm {1}({\textbf {X}}_{t+j}\le {\textbf {u}}) \Big | \mathcal {F}_{t-1}\right] \right) \Bigg |\, \mathcal {F}_{s} \right] \\&= \sum _{j=0}^{u-1} \mathbb {E}\left[ \mathbbm {1}({\textbf {X}}_{t+j}\le {\textbf {u}}) \Big | \mathcal {F}_s\right] -\mathbb {E}\left[ \mathbbm {1}({\textbf {X}}_{t+j}\le {\textbf {u}}) \Big | \mathcal {F}_{s}\right] \quad \text {since }s\le t-1\\&=0. \end{aligned}$$

Since $|U_{t,j}|\le 1$, we have $\max _{t,j}\mathbb {E}[ U_{t,j}^2]\le 1$ and

$$\begin{aligned} H_{u,n}=\mathcal {O}_{\mathbb {P}}(n^{-1/2})\quad \text {for every}\quad u \in \mathbb {N}. \end{aligned}$$

In particular, this implies that for every fixed *u*, $H_{u,n}\xrightarrow {\mathbb {P}}0$. Lastly, for $\sigma ^2_u=\mathop {\operatorname {Var}}\left( M_1(u)\right)$, we have $\frac{1}{\sqrt{n}}\sum _{t=1}^{\lfloor n\tau \rfloor } M_t(u) \overset{\mathcal {D}[0,1]}{\Longrightarrow }\sigma _u B(\tau )$, as a consequence of the central limit theorem for martingale differences (Theorem 23.1 in Billingsley 1968). Hence, $V_{u,n} = \frac{1}{\sqrt{n}}\sum _{t=1}^{\lfloor n\tau \rfloor } M_t(u) + H_{u,n} \overset{\mathcal {D}[0,1]}{\Longrightarrow }\sigma _u B(\tau ).$

The proof of (ii): We have to show that there exists a finite $\sigma ^2:=\lim _{u\rightarrow \infty }\sigma ^2_u.$ By definition

$$\begin{aligned} \sigma ^2_u=\mathop {\operatorname {Var}}\left( M_1(u)\right)&= \mathbb {E}\left[ \left( \sum _{j=0}^{u-1} U_{1+j,j} \right) ^2 \right] \le \sum _{i,j=0}^{u-1}\mathbb {E}\left[ |U_{1+j,j} U_{1+i,i }|\right] \\&\le \sum _{i,j=0}^{u-1} \sqrt{ \mathbb {E}[ U_{1+i,i}^2]} \sqrt{ \mathbb {E}[ U_{1+j,j}^2]} \quad \text {Cauchy--Schwarz} \\&= \left( \sum _{j=0}^{u-1} \sqrt{ \mathbb {E}[ U_{1+j,j}^2]} \right) ^2 \le C \left( \sum _{j=0}^{u-1} \Vert {\textbf {A}}_{j+1}\Vert \right) ^2 \quad \text {Lemma }4. \end{aligned}$$

Finally $\sum _{j=0}^{u-1} \Vert {\textbf {A}}_{j+1}\Vert \le \sum _{j=0}^{\infty } \Vert {\textbf {A}}_{j+1}\Vert <\infty .$ This already concludes point (ii). However, we can also compute the limit $\sigma ^2$ exactly. In fact, from Theorem 8, for all indexes $(t,j),(t',j')$, the expectation $\mathbb {E}[ U_{t,j}U_{t',j'}]\ne 0$ if and only if $t-j=t'-j'$, and thus, $\mathbb {E}[ U_{t,j}U_{t',j'}]= \mathbb {E}[ U_{t,j}U_{t+j'-j,j'}]$. Furthermore, $\left( U_{t,j}U_{t+j'-j,j'} \right) _{t\ge 1}$ is a strictly stationary sequence; hence, for all *t* we have $\mathbb {E}[ U_{t,j}U_{t+j'-j,j'}]= \mathbb {E}[ U_{1,j}U_{1+j'-j,j'}].$ Therefore, for the pairs $(1+j,j),(1+i, i)$, it holds that

$$\begin{aligned} \mathbb {E}[ U_{1+j,j}U_{1+i,i}]=\mathbb {E}[U_{1,j}U_{1+i-j,i}], \end{aligned}$$

and

$$\begin{aligned} \sigma ^2_u&=\!\!\!\sum _{i,j=0}^{u-1}\mathbb {E}\left[ U_{1+j,j} U_{1+i,i } \right] = \sum _{i,j=0}^{u-1}\mathbb {E}\left[ U_{1,j}U_{1+i-j,i}\right] \xrightarrow {u \rightarrow \infty }\!\!\!\sum _{i,j=0}^{\infty }\mathbb {E}\left[ U_{1,j}U_{1+i-j,i}\right] \\&=:\sigma ^2. \end{aligned}$$

Moreover, by decomposition (2), $\left( \mathbbm {1}({\textbf {X}}_t\le {\textbf {u}})- p({\textbf {u}}) \right) \left( \mathbbm {1}({\textbf {X}}_{t'}\le {\textbf {u}}) - p({\textbf {u}}) \right) = \sum _{i,j=0}^\infty U_{t,j}U_{t',i},$

$$\begin{aligned} \sum _{i,j=0}^{\infty }\mathbb {E}\left[ U_{1,j}U_{1+i-j,i}\right]&= \sum _{s\in \mathbb {Z}} \sum _{i,j=0}^{\infty }\mathbb {E}\left[ U_{1,j}U_{1+s,i}\right] \\ &= \sum _{s \in \mathbb {Z}} Cov \left( \mathbbm {1}({\textbf {X}}_s\le {\textbf {u}}), \mathbbm {1}({\textbf {X}}_{1+s}\le {\textbf {u}}) \right) . \end{aligned}$$

For condition (iii), we proceed as follows. Let $\varepsilon >0$, and using Chebyshev’s inequality gives

$$\begin{aligned} \mathbb {P}( |V_{u,n}-W_n|\ge \varepsilon ) = \mathbb {P}\left( \frac{1}{\sqrt{n}}\Bigg | \sum _{t=1}^{\lfloor n \tau \rfloor } \sum _{j=u}^\infty U_{t,j}\Bigg | \ge \varepsilon \right) \le \frac{1}{\varepsilon ^2 n} \mathbb {E}\left[ \left( \sum _{t=1}^{\lfloor n \tau \rfloor } \sum _{j=u}^\infty U_{t,j} \right) ^2 \right] . \end{aligned}$$

The term on the right-hand side can be expanded out further to

$$\begin{aligned}&\mathbb {E}\left[ \left( \sum _{t=1}^{\lfloor n \tau \rfloor } \sum _{j=u}^\infty U_{t,j} \right) ^2 \right] \\&\quad = \sum _{t,s=1}^{\lfloor n \tau \rfloor } \sum _{i,j=u}^\infty \mathbb {E}[ U_{t,j}U_{s,i}] = \sum _{t=1}^{\lfloor n \tau \rfloor } \sum _{i,j=u}^\infty \mathbb {E}[ U_{t,j}U_{t-j+i,i}] \quad \text {Lemma }8 \\&\quad \le \sum _{t=1}^{\lfloor n \tau \rfloor } \sum _{i,j=u}^\infty \Big | \mathbb {E}[ U_{t,j}U_{t-j+i,i}] \Big | \le n \max _{1 \le t\le \lfloor n \tau \rfloor } \sum _{i,j=u}^\infty \Big | \mathbb {E}[ U_{t,j}U_{t-j+i,i}] \Big | \\&\quad \le n \max _{1 \le t\le \lfloor n \tau \rfloor } \sum _{i,j=u}^\infty \sqrt{ \mathbb {E}[ |U_{t,j}|^2]} \sqrt{\mathbb {E}[|U_{t-j+i,i}|^2]} \quad \text {Cauchy--Schwarz inequality}\\&\quad \le n \max _{1 \le t\le \lfloor n \tau \rfloor } C \sum _{i,j=u}^\infty \Vert {\textbf {A}}_{j+1}\Vert \Vert {\textbf {A}}_{i+1}\Vert \quad \text {Lemma }4\\&\quad =n C^2 \left( \sum _{j=u+1}^\infty \Vert {\textbf {A}}_{j}\Vert \right) ^2. \end{aligned}$$

Combining the previous inequalities, we get

$$\begin{aligned} \mathbb {P}( |V_{u,n}-W_n|\ge \varepsilon ) \le \frac{1}{\varepsilon ^2 n} \mathbb {E}\left[ \left( \sum _{t=1}^{\lfloor n \tau \rfloor } \sum _{j=u}^\infty U_{t,j} \right) ^2 \right] \le \frac{C^2}{\varepsilon ^2} \left( \sum _{j=u+1}^\infty \Vert {\textbf {A}}_{j}\Vert \right) ^2. \end{aligned}$$

The term $\sum _{j=u+1}^\infty \Vert {\textbf {A}}_{j}\Vert$ is the tail of the series $\sum _{j=0}^\infty \Vert {\textbf {A}}_{j}\Vert$ which converges by assumption to zero (short-range dependence). Therefore, it holds that

$$\begin{aligned} \liminf _{u\rightarrow \infty }\limsup _{n\rightarrow \infty } \mathbb {P}( |V_{u,n}-W_n|\ge \varepsilon ) \le \liminf _{u\rightarrow \infty } C^2 \left( \sum _{j=u+1}^\infty \Vert {\textbf {A}}_{j}\Vert \right) ^2 =0. \end{aligned}$$

After having checked all conditions, we can now apply Theorem 7 and have thus derived the claim of Theorem 1. $\square$

## Proofs for long-range dependence

### Theorem 8

(Chung 2002, Theorem 1) For ${\textbf {d}}=(d_1,\ldots , d_r)^\top \in (0,1/2)^r$, the notation $\text {diag}(j^{{\textbf {d}} - 1})$ denotes the diagonal matrix with entries $j^{d_1 - 1}, \ldots , j^{d_r - 1}$. We consider the multivariate linear process ${\textbf {X}}_t=\sum _{j=0}^\infty {\textbf {A}}_{j} {\textbf {Z}}_{t-j}$ with coefficients ${\textbf {A}}_{j} \sim \text {diag}(j^{{\textbf {d}}-1} ){\textbf {A}}_{\infty }$ and ${\textbf {A}}_{\infty } \in \text {GL}(\mathbb {R}, r)$ and $({\textbf {Z}}_j)_{j \in \mathbb {Z}}$ an i.i.d. sequence and moment condition $\mathbb {E}[\Vert {\textbf {Z}}_1\Vert ^4]<\infty$. Then,

$$\begin{aligned} \text {diag}\left( n^{-\left( {\textbf {d}}+\frac{1}{2}\right) } \right) \sum _{j=1}^{\lfloor n \tau \rfloor } {\textbf {X}}_j \overset{w}{\Longrightarrow } {\textbf {B}}_{\textbf {d}}(\tau ) \quad \text {for}\quad \tau \in [0,1], \quad \text {as}\quad n \rightarrow \infty , \end{aligned}$$

where $\overset{w}{\Longrightarrow }$ denotes the weak convergence, ${\textbf {B}}_{\textbf {d}}(s)$ is a *r*-dimensional fractional Brownian process $(B_{d_1}(s), \ldots , B_{d_r}(s))^\top$ with covariance matrix $[\eta _{u,v}]_{u,v=1, \ldots , r}\circ {\textbf {A}}_{\infty }^\top \varvec{\Sigma } {\textbf {A}}_{\infty }$. Here, the symbol $\circ$ denotes the Hadamard product, i.e., the component-wise product, between the matrices ${\textbf {A}}_{\infty }^\top \varvec{\Sigma } {\textbf {A}}_{\infty }$ and $[\eta _{u,v}]_{u,v=1, \ldots , r}$, whose components are

$$\begin{aligned} \eta _{uv}&= \frac{\Gamma (d_u) \Gamma (d_v)}{\Gamma (d_u+1) \Gamma (d_v+1)} \\&\quad \left( \frac{1}{1+ d_u+d_v} +\int _0^{\infty } \left( (1+t)^{d_u} - (t)^{d_u}\right) \left( (1+t)^{d_v} - (t)^{d_v}\right) \right) \, dt. \end{aligned}$$

### Remark 4

For the following identity holds for $d\in (0,1/2)$ (see Beran et al. 2013, p. 35):

B5 $$\begin{aligned} \frac{1}{2d+1} +\int _0^\infty \left( (1+t)^d - t^d)\right) ^2\, dt = \frac{\Gamma (1+d)^2}{\Gamma (2d+2) \sin \left( \frac{\pi }{2} +\pi d \right) }. \end{aligned}$$

Therefore, for $$d_1=\ldots =d_r=d$$

$$\begin{aligned} \eta _{ij}=\frac{\Gamma (d)^2 \Gamma (1+d)^2}{\Gamma (1+d)^2 \Gamma (2d+2) \sin \left( \frac{\pi }{2} +\pi d \right) }=\frac{\Gamma (d)^2 }{\Gamma (2d+2) \cos \left( \pi d \right) }, \end{aligned}$$

and, for $$\tau =1$$

$$\begin{aligned} n^{\frac{1}{2}-d} \bar{{\textbf {X}}}_n \xrightarrow {\mathcal {D}} \mathcal {N}\left( {\textbf {0}}, V\right) , \quad V:= \frac{\Gamma (d)^2 }{\Gamma (2d+2) \cos \left( \pi d \right) } {\textbf {A}}_{\infty }^\top \varvec{\Sigma } {\textbf {A}}_{\infty }. \end{aligned}$$

### Proof of Theorem 2

The proof follows from an application of the reduction principle in Appendix C. In fact, assumption (C18) of Theorem 10 is satisfied as it is also one of the assumptions of Theorem 2, and ${\textbf {X}}_t= \sum _{j=0}^\infty {\textbf {A}}_{j} {\textbf {Z}}_{t-j},$ and $({\textbf {Z}}_j)_{j \in \mathbb {Z}}$ forms a multivariate stationary white noise in $\mathbb {R}^r$ with variance $\varvec{\Sigma }$ and, $\mathbb {E}[\Vert {\textbf {Z}}_1\Vert ^4]< \infty .$ Therefore, for any fixed ${\textbf {x}}\in \mathbb {R}^r$,

B6 $$\begin{aligned} n^{\frac{1}{2} -d } \Bigg | \frac{1}{n}\sum _{j=1}^n \mathbbm {1}\left( {\textbf {X}}_{j} \le {\textbf {x}} \right) - p({\textbf {x}}) + \nabla p({\textbf {x}}) \bar{{\textbf {X}}}_n \Bigg | \xrightarrow {\mathbb {P}} 0. \end{aligned}$$

Furthermore, Theorem 1 in Chung (2002) (reproduced as Theorem 8 above) guarantees that

$$\begin{aligned}&n^{1/2-d} (\nabla p({\textbf {x}}))^{\top } \bar{{\textbf {X}}}_n \xrightarrow {\mathcal {D}} \mathcal {N}\left( 0, \sigma ^2 \right) \quad \text {where}\quad \sigma ^2:= \frac{\Gamma (d)^2 }{\Gamma (2d+2)\cos {(\pi d)}} \\&\quad \left( \nabla p({\textbf {x}}) \right) ^{\top } {\textbf {A}}_{\infty } \varvec{\Sigma } {\textbf {A}}_{\infty }^{\top } \nabla p({\textbf {x}}). \end{aligned}$$

Thus, by Theorem 4.1 in Billingsley (1968), we obtain

$$\begin{aligned} n^{\frac{1}{2} -d } \Bigg ( \frac{1}{n}\sum _{j=1}^n \mathbbm {1}\left( {\textbf {X}}_ {j}\le {\textbf {x}} \right) - p({\textbf {x}})\Bigg ) \xrightarrow {\mathcal {D}} \mathcal {N}(0,\sigma ^2). \end{aligned}$$

$$\square $$

In the following proposition we give a criteria used to verify Assumption (C18) when we consider the process ${\textbf {X}}_t:=(X_t, \ldots , X_{t+r-1})^\top$ for $X_t=\sum _{j=0}^\infty a_j Z_{t-j}$. More specifically,

### Proposition 9

Consider the process $$X_t=\sum _{j=0}^\infty a_j Z_{t-j}$$ with $$a_0\ne 0$$, $$(Z_j)_{j \in \mathbb {N}}$$ forming an independent and identically distributed sequence with $$Z_1 $$ admitting a continuous and bounded probability density function *f*. For $$r\ge 1$$, we define $${\textbf {X}}_t =(X_t, \ldots , X_{t+r-1})^\top $$. If *f* admits a bounded derivative, then (C18) is satisfied for $${\textbf {X}}_t$$ with $$s_0:=r$$.

### Proof of Proposition 9

It is enough to show that for any ${\textbf {x}}=(x_0, \ldots , x_{r-1})^\top$, there exists a constant $C>0$ independent of ${\textbf {x}}$ such that, for all $j\ge r$

B7 $$\begin{aligned} \left| p_j(x_0, \ldots , x_{r-1})\right|\le &  C,\quad \left| \nabla p_j(x_0, \ldots , x_{r-1})\right| \le C\, \, \text {and}\, \, \left| \nabla ^2 p_j(x_0, \ldots , x_{r-1})\right| \nonumber \\\le &  C. \end{aligned}$$

The idea is to prove (B7) by induction, using the following recursive relation, which holds for all $j\ge 1$

$$\begin{aligned} p_{j}( {\textbf {x}})=\int _{\mathbb {R}} p_{j-1} ({\textbf {x}}- {\textbf {A}}_{j} {\textbf {t}} ) \, d\mathcal {G}({\textbf {t}}). \end{aligned}$$

In fact, if (B7) holds for $j=r$ for some constant *C* (under this assumption we can use the dominated convergence theorem since a constant is integrable with respect to $d\mathcal {G}(\cdot )$), then for all $j\ge r+1$

$$\begin{aligned} \nabla p_{j}( {\textbf {x}}) =&\int _{\mathbb {R}} \nabla p_{j-1}({\textbf {x}}- {\textbf {A}}_{j} {\textbf {t}} )\, d\mathcal {G}({\textbf {t}}) \le C \int _{\mathbb {R}} \, d\mathcal {G}({\textbf {t}}) =C, \end{aligned}$$

and similarly $\nabla ^2 p_j ({\textbf {x}}) \le C.$ So, we only have to prove the inductive base for $j=r$. We fix additional notation:

1. For any generic random variable *U* in $\mathbb {R}^r$, $f_U (u_0, \ldots , u_{r-1}) = f_U({\textbf {u}})$ denotes its probability density function in $\mathbb {R}^r$. For instance, $f_{{\textbf {X}}_{t,r}}(x_0,\ldots , x_{r-1})$ denotes the probability density function of ${\textbf {X}}_{t,r}$ in $\mathbb {R}^{r}$.
2. The coordinates of a generic *m*-dimensional random vector ${\textbf {U}}$ are denoted by ${\textbf {U}}=(U^{(0)}, \ldots , U^{(m-1)})^\top$.
3. For a matrix $A \in \mathbb {R}^{a\times b}$, we denote by $A^{-(j)}_{-(i)}$ the submatrix obtaining by removing the *i*-th row and the *j*th column of *A*. The symbol $A^{-(j_1, \ldots , j_{s_1})}_{-(i_1, \ldots , i_{s_2})}$ indicates the submatrix (or minor) obtained by removing all columns and rows indexed by $(j_1, \ldots , j_{s_1})$ and $(i_1, \ldots , i_{s_2})$. For vectors, we will not make a distinction between a row and column vector, and simply denote by ${\textbf {U}}^{-(i)}$ the vector obtained by removing the *i*th coordinate from ${\textbf {U}}.$
4. We define ${\textbf {X}}_{t,r} = B {\textbf {Z}}_{t,r}$ where ${\textbf {Z}}_{t,r}:= ( Z_t, Z_{t+1}, \ldots , Z_{t+r-1})^{\top }$ and *B* is invertible. The matrix *B* was defined in (6). Since *B* is invertible, by the transformation formula, $f_{{\textbf {X}}_{t,r}}(x_0, \ldots , x_{r-1})= f_{{\textbf {Z}}_{t,r}} (B^{-1} {\textbf {x}}) |\det (B^{-1})|.$

Observe that by exploiting the structure of ${\textbf {Z}}_{t,r}$ we obtain the following: for all ${\textbf {v}}=(v_0, \ldots , v_{r-1})^\top$ and for all $\ell =0,\ldots , r-1,$

$$\begin{aligned} f_{{\textbf {Z}}_{{\textbf {t,r}}}}(v_0, \ldots , v_{r-1})&= f_{Z_{t}} (v_0) \cdots f_{Z_{t+r-1}}(v_{r-1})\\&\le C f_{Z_{t}}(v_0)\cdots f_{Z_{t+(\ell -1)}}(v_{\ell -1})f_{Z_{t+(\ell +1)}}(v_{\ell +1}) \cdots f_{Z_{t+r-1}}( v_{r-1})\\&= C f_{{\textbf {Z}}_{t,r}^{-(\ell )}} \left( {\textbf {v}}^{-(\ell )}\right) . \end{aligned}$$

Similarly, for the derivative,

$$\begin{aligned} \partial _\ell f_{{\textbf {Z}}_{{\textbf {t,r}}}}(v_0, \ldots , v_{r-1})&= f_{Z_{t}} (v_0)\cdots f_{Z_{t+(\ell -1)}}(v_{\ell -1}) f'_{Z_{t+\ell }}(v_\ell ) f_{Z_{t+(\ell +1)}}(v_{\ell +1})\\&\quad \ldots f_{Z_{t+(\ell +r-1)}}(v_{\ell +r-1}) \\&\le C f_{{\textbf {Z}}_{t,r}^{-(\ell )}}({\textbf {v}} ^{-(\ell )}). \end{aligned}$$

Since the previous two inequalities hold for any choice of $v_0, \ldots , v_{r-1}$, if $\mathcal {K}_{(0)},\ldots , \mathcal {K}_{(r-1)}$ are the rows of the matrix $B^{-1}$, for any $\ell =0, \ldots , r-1$ we have

$$\begin{aligned} f_{{\textbf {Z}}_{{\textbf {t,r}}}} ( \mathcal {K}{\textbf {u}})&= f_{{\textbf {Z}}_{{\textbf {t,r}}}} ( \mathcal {K}_{(0)} \cdot {\textbf {u}}, \ldots , \mathcal {K}_{(r-1)} \cdot {\textbf {u}}) \\ &\le C f_{{\textbf {Z}}_{t,r}^{-(\ell )}} ( \mathcal {K}_{(0)} \cdot {\textbf {u}}, \ldots , \mathcal {K}_{(\ell -1)} \cdot {\textbf {u}}\,,\, \mathcal {K}_{(\ell +1)} \cdot {\textbf {u}},\ldots , \mathcal {K}_{(r-1)} {\textbf {u}}) \\ &= C f_{{\textbf {Z}}_{t,r}^{-(\ell )}} ( \mathcal {K}_{-(\ell )} {\textbf {u}}). \end{aligned}$$

Furthermore, by definition of the matrix–vector product, for all $\ell =0, \ldots , r-1$ we have

$$\begin{aligned} \mathcal {K}_{-(\ell )} {\textbf {u}} = \mathcal {K}_{-(\ell )} ^{-(\ell )} {\textbf {u}}^{-(\ell )} + u^{(\ell )} \mathcal {K}^{(\ell )}_{-(\ell )}, \end{aligned}$$

so that,

B8 $$\begin{aligned} f_{{\textbf {Z}}_{{\textbf {t,r}}}} ( \mathcal {K}{\textbf {u}}) \le C f_{{\textbf {Z}}_{t,r}^{-(\ell )}} \left( \mathcal {K}_{-(\ell )} ^{-(\ell )} {\textbf {u}}^{-(\ell )} + u^{(\ell )} \mathcal {K}^{(\ell )}_{-(\ell )} \right) \quad \text {for}\quad \ell =0, \ldots , r-1. \end{aligned}$$

Lastly, note that $\mathcal {K}_{-(\ell )} ^{-(\ell )}$ is invertible for every $\ell =0,\ldots , r-1$. This follows since $\mathcal {K}^{(\ell )}_{\ell }$ s a submatrix of $B^{-1}$ and since *B* is a lower triagular Toeplitz matrix. It can be shown that $B^{-1}$ is of the form

$$\begin{aligned}B^{-1} =\left( \begin{array}{cccc} a_0^{-1}& & & \\ *& a_{0}^{-1} & & \\ \vdots & \ddots & \ddots & \\ *& \cdots & *& a_0^{-1} \end{array}\right) . \end{aligned}$$

Every submatrix obtained by removing rows and columns corresponding to the same indexes, meaning $\mathcal {K}^{-(i_1,\ldots , i_h)}_{-(i_1,\ldots , i_h)}$, is also a lower triangular Toeplitz matrix; thus, it is invertible.

We are ready to bound the derivatives of $p_r$.

1. *Partial derivatives* for all $\ell =0, \ldots , r-1$
  B9 $$\begin{aligned} \partial _\ell p_r(x_0, \ldots , x_{r-1})&= \int _{-\infty }^{x_0}\cdots \int _{-\infty }^{x_{\ell -1}} \int _{-\infty }^{x_{\ell +1}} \cdots \nonumber \\&\quad \int _{-\infty }^{x_{r-1}}\, f_{{\textbf {Z}}_{t,r}}(\mathcal {K}^{-(\ell )} {\textbf {u}}^{-(\ell )} + x_\ell \mathcal {K}^{(\ell )} )\, d{\textbf { u}}^{-(\ell )}, \end{aligned}$$
  B10 $$\begin{aligned}&\le C \int _{\mathbb {R}^{r-1}} f_{{\textbf {Z}}_{t,r}^{-(\ell )}}(\mathcal {K}^{-(\ell )}_{-(\ell )} {\textbf {u}}^{-(\ell )} + x_\ell \mathcal {K}^{(\ell )}_{-(\ell )} ) d{\textbf { u}}^{-(\ell )} \nonumber \\&\le C. \end{aligned}$$
  The last inequality follows from the fact that $f_{{\textbf {Z}}_{t,r}^{-(\ell )}}$ is integrable and $\mathcal {K}^{-(\ell )}_{-(\ell )}$ is invertible; thus, a change of variables yields the result.
2. *Partial second derivatives* for any $\ell =0, \ldots , r-1$ and $s=0, \ldots , r-1$. Firstly, we study the case $s=\ell$, i.e.,
  B11 $$\begin{aligned}&\partial ^2_{\ell ,\ell }p_r(x_0, \ldots ,x_{r-1}) \end{aligned}$$
  B12 $$\begin{aligned}&\quad = \int _{-\infty }^{x_0}\cdots \int _{-\infty }^{x_{\ell -1}} \int _{-\infty }^{x_{\ell +1}} \cdots \nonumber \\&\qquad \int _{-\infty }^{x_{\ell -1}}\, \int _{\mathbb {R}} \left( \sum _{j=0}^{r-1} \mathcal {K}_{j,\ell } \partial _j f_{{\textbf {Z}}_{t,r}}(\mathcal {K}^{-(\ell )} {\textbf {u}}^{-(\ell )} + x_\ell \mathcal {K}^{(\ell )}_{-(\ell )} ) \right) \, \, d{\textbf { u}}^{-(\ell )}; \end{aligned}$$
  thus,
  B13 $$\begin{aligned} \Bigg | \partial ^2_{\ell ,\ell }p_r (x_0, \ldots ,x_{r-1})\Bigg |&\le C \sum _{j=0}^{r-1} \int _{\mathbb {R}^{r-1}} f_{{\textbf {Z}}_{t,r}^{-(j)}}(\mathcal {K}^{-(\ell )} _{-(j)} {\textbf {u}}^{-(\ell )}\nonumber \\&\quad + x_\ell \mathcal {K}^{(\ell )}_{-(j)} ) d{\textbf { u}}^{-(\ell )} \le C. \end{aligned}$$
  If $$s\ne r$$
  B14 $$\begin{aligned}&\partial ^2_{s,\ell }p_r(x_0, \ldots ,x_{r-1}) \end{aligned}$$
  B15 $$\begin{aligned}&\quad = \int _{-\infty }^{x_0}\cdots \int _{-\infty }^{x_{s-1}} \int _{-\infty }^{x_{s+1}} \cdots \int _{-\infty }^{x_{\ell -1}} \end{aligned}$$
  B16 $$\begin{aligned}&\quad \int _{-\infty }^{x_{\ell +1}} \cdots \int _{-\infty }^{x_{\ell -1}}\, f_{{\textbf {Z}}_{t,r}}(\mathcal {K}^{-(\ell ,s)} {\textbf {u}}^{-(\ell )} + x_\ell \mathcal {K}^{(\ell )} + x_s \mathcal {K}^{(s)})\, \,d{\textbf {u}}^{-(\ell ,s)} \end{aligned}$$
  B17 $$\begin{aligned}&\quad \le \int _{\mathbb {R}^{r-2}} f_{{\textbf {Z}}_{t,r}^{-(\ell ,s)}} \left( \mathcal {K}^{-(\ell ,s)}_{-(\ell ,s)} {\textbf {u}}^{-(\ell ,s)} + x_\ell \mathcal {K}^{(r)}_{-(\ell ,s)} + x_s \mathcal {K}^{-(s)}_{-(\ell ,s)} \right) d{\textbf {u}}^{-(\ell ,s)} \nonumber \\&\quad \le C. \end{aligned}$$

$$\square $$

## Reduction principle

In this section, we derive the reduction principle for multivariate linear processes that are long-range dependent. All the auxiliary results are listed in the subsection following the proof of Theorem 10.

### Theorem 10

(Reduction Principle) Consider a multivariate linear process

$$\begin{aligned} {\textbf {X}}_t = \sum _{j=0}^\infty {\textbf {A}}_{j} {\textbf {Z}}_{t-j}, \quad {\textbf {A}}_{j} \overset{j \rightarrow \infty }{\sim }\ j^{d-1} {\textbf {A}}_{\infty } \in \text {GL}(\mathbb {R}, r), \quad d \in \left( 0, \frac{1}{2} \right) , \end{aligned}$$

with i.i.d. innovations $({\textbf {Z}}_j)_{j \in \mathbb {Z}}$ with variance $\varvec{\Sigma }$ and moment condition $\mathbb {E}[\Vert {\textbf {Z}}_1\Vert ^4] < \infty$. If there exists a positive integer $s_0$ and a constant $C > 0$ such that

C18 $$\begin{aligned} \sup _{{\textbf {x}}\in \mathbb {R}^r} \, \max _{s\ge s_0} \left( |p_s({\textbf {x}}) | + \sum _{i=1}^r | \partial _i p_s({\textbf {x}}) | + \sum _{i, j=1}^r | \partial ^2_{i,j} p_s({\textbf {x}}) | \right) <C, \end{aligned}$$

then, for any fixed vector ${\textbf {x}} \in \mathbb {R}^r$,

C19 $$\begin{aligned} n^{\frac{1}{2} - d} \left| \frac{1}{n} \sum _{t=1}^n \mathbbm {1}( {\textbf {X}}_t \le {\textbf {x}} )- p({\textbf {x}}) + \nabla p({\textbf {x}}) \bar{{\textbf {X}}}_n \right| \xrightarrow {\mathbb {P}} 0. \end{aligned}$$

### Proof of Theorem 10

For a fixed ${\textbf {x}} \in \mathbb {R}^{r},$ set

$$\begin{aligned} S_n({\textbf {x}}) := \sum _{t=1}^n\mathbbm {1} \left( \{{\textbf {X}}_t \le {\textbf {x}} \}\right) - np({\textbf {x}}) + n\nabla p({\textbf {x}}) \bar{{\textbf {X}}}_n, \end{aligned}$$

so that (C19) is equivalently expressed as $n^{- (\frac{1}{2}+d) } |S_n({\textbf {x}}) |\xrightarrow {\mathbb {P}}0.$ Hence,

$$\begin{aligned} S_n ({\textbf {x}})= \sum _{t=1}^n \sum _{s=1}^{\infty } \Big ( p_{s-1}({\textbf {x}} -{\textbf {R}}_{t,s-1}) - p_{s}({\textbf {x}} -{\textbf {R}}_{t,s}) \Big ) + \nabla p({\textbf {x}})n\bar{{\textbf {X}}}_n. \end{aligned}$$

The leading term of the main sum is the point-wise difference of *nearly* the same functions $p_s, p_{s-1}$ evaluated at two close points. We split $S_n({\textbf {x}})$ in three sums,

C20 $$\begin{aligned} \begin{aligned} S_n({\textbf {x}})&=\sum _{t=1}^n\sum _{s=1}^{\infty } \Big ( p_{s-1}({\textbf {x}} -{\textbf {R}}_{t,s-1}) - p_{s}({\textbf {x}} -{\textbf {R}}_{t,s}) \Big )+ \nabla p({\textbf {x}})n\bar{{\textbf {X}}}_n \\&=\sum _{t=1}^n \sum _{s=1}^{\infty } \Big ( p_{s-1}({\textbf {x}} -{\textbf {R}}_{t,s-1}) - p_{s}({\textbf {x}} -{\textbf {R}}_{t,s}) \pm \mathbbm {1}_{\{s\ge r\}}\nabla p_{s-1}({\textbf {x}} -{\textbf {R}}_{t,s}) A_s {\textbf {Z}}_{t-s}\Big ) \\&\quad + \nabla p({\textbf {x}}) \sum _{t=1}^n \sum _{s=1}^{\infty } A_s {\textbf {Z}}_{t-s} \\&=\underbrace{\sum _{t=1}^n \sum _{s=1}^{\infty } \Big ( p_{s-1}({\textbf {x}} -{\textbf {R}}_{t,s-1}) - p_{s}({\textbf {x}} -{\textbf {R}}_{t,s}) + \mathbbm {1}_{\{s\ge r\}}\nabla p_{s-1}({\textbf {x}} -{\textbf {R}}_{t,s}) A_s {\textbf {Z}}_{t-s} \Big )}_{=:T_n^{(1)}({\textbf {x}})} \\&\quad -\sum _{t=1}^n \sum _{s=1}^{\infty } \mathbbm {1}_{\{s\ge r\}}\nabla p_{s-1}({\textbf {x}} - {\textbf {R}}_{t,s}) A_s {\textbf {Z}}_{t-s}\\&\quad +\nabla p({\textbf {x}}) \sum _{t=1}^n\left( \sum _{s=0}^{r-1}A_s{\textbf {Z}}_{t-s}+\sum _{s=r}^{\infty } A_s {\textbf {Z}}_{t-s} \right) \\&= T_n^{(1)}({\textbf {x}})+ \underbrace{\sum _{t=1}^n\sum _{s=r}^{\infty } \Big ( \nabla p({\textbf {x}}) -\nabla p_{s-1}({\textbf {x}} - {\textbf {R}}_{t,s})\Big )^{\top } A_s {\textbf {Z}}_{t-s}}_{=:T_n^{(2)}({\textbf {x}})}\\&\quad + \underbrace{\nabla p({\textbf {x}}) \sum _{t=1}^n \sum _{s=0}^{r-1} A_s {\textbf {Z}}_{t-s}}_{=:T_n^{(3)}({\textbf {x}})}. \end{aligned} \end{aligned}$$

To conclude that $n^{-( \frac{1}{2}+d) } |S_n ({\textbf {x}})|\xrightarrow {\mathbb {P}}0$, it is enough to show that $n^{-( \frac{1}{2}+d) } |T_n^{(m)} ({\textbf {x}})|\xrightarrow {\mathbb {P}}0$ for $m=1, 2,3.$ In fact, by Chebyshev’s inequality, for every fixed $\varepsilon >0,$

$$\begin{aligned} \mathbb {P}\left( |T_n^{(m)} ({\textbf {x}})|> \varepsilon n^{d+1/2}\right) \le \frac{\text {Var}(T_n^{(m)} ({\textbf {x}}))}{\varepsilon ^2 n^{2d+1}}\xrightarrow {n\rightarrow \infty }0, \quad \text {for}\quad m=1,2,3. \end{aligned}$$

To complete the proof, we will now show that there exists $\xi >0$ sufficiently small such that

(i) $$ \mathop {\operatorname {Var}}\left( T_{n}^{(1)}({\textbf {x}})\right) = \mathcal {O}( n^{2d})$$
(ii) $$ \mathop {\operatorname {Var}}\left( T_{n}^{(3)}({\textbf {x}})\right) = \mathcal {O}( n^{})$$
(iii) $$\mathop {\operatorname {Var}}\left( T_{n}^{(2)}({\textbf {x}})\right) = {\left\{ \begin{array}{ll} \mathcal {O}(n^{})& \text {for}\quad d\in (0,1/4)\\ \mathcal {O}(n^{4d+\zeta })& \text {for}\quad d\in [1/4,1/2)\\ \end{array}\right. }.$$

*Proof for (i):* Defining

$$\begin{aligned} K_{t,j}({\textbf {x}}) :=&p_{j-1}({\textbf {x}} - {\textbf {R}}_{t,j-1}) - p_{j}({\textbf {x}} - {\textbf {R}}_{t,j}) + \mathbbm {1}_{\{j\ge r\}} \nabla p_{j-1}({\textbf {x}} - {\textbf {R}}_{t,j}) {\textbf {A}}_{j} {\textbf {Z}}_{t-j}, \end{aligned}$$

we have $T_{N}^{(1)}({\textbf {x}}) = \sum _{t=1}^{n} \sum _{j=1}^{\infty } K_{t,j}({\textbf {x}}).$ From Lemma 8, $\mathbb {E}\left[ K_{t,j}({\textbf {x}}) K_{t',j'} ({\textbf {x}})\right] =0$ whenever $j'\ne t'-t+j$. For the product $\mathbb {E}\left[ K_{t,j} ({\textbf {x}}) K_{t',t'-t+j}({\textbf {x}})\right]$, using Lemma 9 (which can be applied because of assumption (C18)), there exist positive constants $C_1,C_2, C_3, C_4$ such that

$$\begin{aligned}&K_{t,j} ({\textbf {x}}) \, K_{t',t'-t+j}({\textbf {x}}) \\&\le C_1 C_3 \, \Vert {\textbf {A}}_{j}\Vert ^2 \, \Vert {\textbf {A}}_{t'-t+j}\Vert ^2 \cdot \Big ( \Vert {\textbf {Z}}_{t-j}\Vert ^2 \, \Vert {\textbf {Z}}_{t'-(t'-t+j)}\Vert ^2 \\&\quad + C_4 \Vert {\textbf {Z}}_{t-j}\Vert ^2 + C_2 \Vert {\textbf {Z}}_{t'-(t'-t+j)}\Vert ^2 + C_2 C_4 \Big ). \end{aligned}$$

Therefore, by taking expectations on both sides and using the fact that ${\textbf {A}}_{j} \sim j^{d-1} {\textbf {A}}_{\infty }$ and that the forth moment of ${\textbf {Z}}_1$ is bounded by assumption, we obtain $\mathbb {E}\left[ K_{t,j} ({\textbf {x}}) K_{t',t'-t+j}({\textbf {x}})\right] \le C j^{2(d-1)} (j+ t'-t)^{2(d-1)}.$ Using the arguments above and because of $d<1$,

$$\begin{aligned} \mathop {\operatorname {Var}}\left( T_{n}^{(1)}({\textbf {x}})\right)&\le \mathbb {E}\left[ \left( \sum _{t=1}^{n} \sum _{j=1}^{\infty } K_{t,j}\right) ^2 \right] = \mathbb {E}\left[ \sum _{t=1}^{n} \sum _{j=1}^{\infty } \sum _{t'=1}^{n} \sum _{j'=1}^{\infty } K_{t,j} K_{t',j'}\right] \\&= \,\sum _{t'=1}^{n} \sum _{j=1}^{\infty } \sum _{t=1}^{\min \{n, (t'+j-1)\}} \mathbb {E}\left[ K_{t,j} K_{t',t'-t+j}\right] \\&\le C \,\sum _{t'=1}^{n} \sum _{t=1}^{n} \sum _{j=1}^{\infty } j^{2(d-1)} (j+ |t'-t|)^{2(d-1)}\; \quad \\&\le C \,\sum _{t'=1}^{n} \sum _{t=1}^{n} (t'-t)^{2(d-1)} \le C n^{2d }, \quad \text {using} \ |t'-t|\le n. \end{aligned}$$

*Proof for (ii):* By definition of $T_n^{(3)}({\textbf {x}})$ and the Cauchy–Schwarz inequality, $\left( T_n^{(3)}({\textbf {x}})\right) ^2= (\nabla p ({\textbf {x}}) \sum _{t=1}^{n}\sum _{i=1}^{r-1} {\textbf {A}}_{i} {\textbf {Z}}_{t-i})^2 \le C \Vert \nabla p({\textbf {x}}) \Vert ^2 \Vert \sum _{t=1}^{n} \sum _{i=1}^{r-1} {\textbf {A}}_{i} {\textbf {Z}}_{t-i} \Vert ^2 \le C \sum _{t,t'=1}^n \sum _{i,j=1}^{r-1} \langle {\textbf {A}}_{i} {\textbf {Z}}_{t-i}, {\textbf {A}}_{j} {\textbf {Z}}_{t'-j}\rangle .$ Moreover, using the independence of the sequence $({\textbf {Z}}_j)_{j \in \mathbb {N}},$ $\mathop {\operatorname {Var}}\left( T_{n}^{(3)}({\textbf {x}})\right) \le \mathbb {E}[T_{n}^{(3)}({\textbf {x}})^2]\le C n.$

*Proof for (iii):* By definition, $T_{n}^{(2)}({\textbf {x}}) =\sum _{t=1}^{n} \sum _{j=r}^{\infty } h_{t,j} {\textbf {A}}_{j} {\textbf {Z}}_{t-j}$ with

$$\begin{aligned} h_{t,j}({\textbf {x}}) := \left( \nabla p({\textbf {x}} - {\textbf {R}}_{t,j}) - \nabla p_{j-1}({\textbf {x}} - {\textbf {R}}_{t,j}) \right) ^\top . \end{aligned}$$

By the convention established at the beginning of this article, gradients are considered row vectors. Therefore, we obtain

$$\begin{aligned} \mathop {\operatorname {Var}}\left( T_{n}^{(2)}({\textbf {x}})\right)&\le \mathbb {E}\Big ( \sum _{t=1}^{n} \sum _{j=r}^{\infty } h_{t,j}({\textbf {x}}) {\textbf {A}}_{j} {\textbf {Z}}_{t-j}\Big )^2 \\&\le \!\!\sum _{t=1}^{n} \sum _{j=r}^{\infty } \sum _{t'=t}^{n} \mathbb {E}\Big ( h_{t,j}({\textbf {x}}) {\textbf {A}}_{j} {\textbf {Z}}_{t-j} h_{t',j'}({\textbf {x}}) {\textbf {A}}_{j'} {\textbf {Z}}_{t'-j'}\Big ),\quad t'-t+j=j', \end{aligned}$$

where we used again the fact that if $t-j \ne t' - j'$, then

$$\begin{aligned} \mathbb {E}\Big (h_{t,j}({\textbf {x}}){\textbf {A}}_{j} {\textbf {Z}}_{t-j}h_{t',j'}({\textbf {x}}){\textbf {A}}_{j'} {\textbf {Z}}_{t'-j'} \Big ) = 0. \end{aligned}$$

In fact, $R_{t,j}= \sum _{i=j+1}^\infty {\textbf {A}}_{i} {\textbf {Z}}_{t-i} = \tilde{f}({\textbf {Z}}_{t-j-1},{\textbf {Z}}_{t-j-2}, \ldots )$ for some function $\tilde{f}$. Thus, $h_{t,j}({\textbf {x}}) {\textbf {A}}_{j} {\textbf {Z}}_{t-j}= f_1( {\textbf {Z}}_{t-j-1},{\textbf {Z}}_{t-j-2}, \ldots ) {\textbf {A}}_{j} {\textbf {Z}}_{t-j}$ and $\sigma ({\textbf {Z}}_{t-j})$ is independent from $\mathcal {F}_{t-j-1}= \sigma ( {\textbf {Z}}_{t-j-1},{\textbf {Z}}_{t-j-2}, \ldots ).$ Then, if $t-j \ne t'-j'$ (we can assume $t'-j'<t-j$)

$$\begin{aligned}&\mathbb {E}[h_{t,j}({\textbf {x}}) {\textbf {A}}_{j} {\textbf {Z}}_{t-j} h_{t',j'}({\textbf {x}}) {\textbf {A}}_{j'} {\textbf {Z}}_{t'-j'} ]\\&\quad = \mathbb {E}[ f_1( {\textbf {Z}}_{t-j-1},{\textbf {Z}}_{t-j-2}, \ldots ) {\textbf {A}}_{j} {\textbf {Z}}_{t-j} f_2( {\textbf {Z}}_{t'-j'-1},{\textbf {Z}}_{t'-j'-2}, \ldots ) {\textbf {A}}_{j'} {\textbf {Z}}_{t'-j'} ]\\&\quad = \mathbb {E}\left[ \mathbb {E}\left[ f_1( {\textbf {Z}}_{t-j-1},{\textbf {Z}}_{t-j-2}, \ldots ) {\textbf {A}}_{j} {\textbf {Z}}_{t-j} f_2( {\textbf {Z}}_{t'-j'-1},{\textbf {Z}}_{t'-j'-2}, \ldots ) {\textbf {A}}_{j'} {\textbf {Z}}_{t'-j'}\, \Big | \, \mathcal {F}_{t'-j'}\right] \right] \\&\quad = \mathbb {E}\left[ f_2( {\textbf {Z}}_{t'-j'-1},{\textbf {Z}}_{t'-j'-2}, \ldots ) {\textbf {A}}_{j'} {\textbf {Z}}_{t'-j'} \mathbb {E}\left[ f_1( {\textbf {Z}}_{t-j-1},{\textbf {Z}}_{t-j-2}, \ldots ) {\textbf {A}}_{j} {\textbf {Z}}_{t-j} \, \Big | \, \mathcal {F}_{t'-j'}\right] \right] \\&\quad =\mathbb {E} \Big [ f_2( {\textbf {Z}}_{t'-j'-1}, {\textbf {Z}}_{t'-j'-2}, \ldots ) \cdot {\textbf {A}}_{j'} {\textbf {Z}}_{t'-j'} {\textbf {A}}_{j} \\&\qquad \cdot \mathbb {E}\left[ {\textbf {Z}}_{t-j} \, \Big | \, \mathcal {F}_{t'-j'}\right] \cdot \mathbb {E}\left[ f_1( {\textbf {Z}}_{t-j-1}, {\textbf {Z}}_{t-j-2}, \ldots ) \, \Big | \, \mathcal {F}_{t'-j'}\right] \Big ]\\&= \mathbb {E}\left[ f_2( {\textbf {Z}}_{t'-j'-1},{\textbf {Z}}_{t'-j'-2}, \ldots ) {\textbf {A}}_{j'} {\textbf {Z}}_{t'-j'} {\textbf {A}}_{j} \mathbb {E}\left[ {\textbf {Z}}_{t'-j'} \right] \right. \\&\quad \left. \mathbb {E}\left[ f_1( {\textbf {Z}}_{t-j-1},{\textbf {Z}}_{t-j-2}, \ldots ) \, \Big | \, \mathcal {F}_{t'-j'}\right] \right] \\&=0. \end{aligned}$$

The same argument that proved (A3) yields

$$\begin{aligned} p({\textbf {x}})&= \mathbb {P}( {\textbf {X}}_{t,j-1}+ {\textbf {R}}_{t,j-1} \le {\textbf {x}} ) = \int \mathbb {P}( {\textbf {X}}_{t,j-1} \\&\le {\textbf {x}} - {\textbf {t}})dF_{ {\textbf {R}}_{t,j-1}}({\textbf {t}}) = \int p_{j-1}({\textbf {x}} - {\textbf {t}})dF_{ {\textbf {R}}_{t,j-1}}({\textbf {t}}). \end{aligned}$$

The partial derivatives going into the above formula are bounded by assumption by a constant, and every constant is integrable with respect to the measure $dF_{{\textbf {R}}_{t,j-1}}$. Hence, by applying the dominated convergence theorem

$$\begin{aligned} h_{t,j}({\textbf {x}})&= \int \Big ( \nabla p_{j-1}({\textbf {x}} - {\textbf {t}}) - \nabla p_{j-1}({\textbf {x}} -{\textbf {R}}_{t,j})\Big ) dF_{ {\textbf {R}}_{t,j-1}}({\textbf {t}}). \end{aligned}$$

Therefore, applying the Cauchy–Schwarz inequality and Jensen’s inequality, we obtain

$$\begin{aligned} \Bigl | h_{t,j}( {\textbf {x}}){\textbf {A}}_{j} {\textbf {Z}}_{t-j} \Bigr |&= \bigl | \langle h_{t,j}( {\textbf {x}}), {\textbf {A}}_{j} {\textbf {Z}}_{t-j} \rangle \bigl | \\&\le C \Vert {\textbf {A}}_{j} {\textbf {Z}}_{t-j} \Vert \int \Vert {\textbf {R}}_{t,j}-{\textbf {t}} \Vert dF_{ {\textbf {R}}_{t,j-1}}({\textbf {t}})\\&\le C \Vert {\textbf {A}}_{j} {\textbf {Z}}_{t-j} \Vert \left( \, \int \Vert {\textbf {R}}_{t,j}\Vert +\Vert {\textbf {t}} \Vert dF_{ {\textbf {R}}_{t,j-1}}({\textbf {t}}) \right) \\&\le C \Vert {\textbf {A}}_{j} {\textbf {Z}}_{t-j} \Vert \big ( \Vert {\textbf {R}}_{t,j}\Vert +\mathbb {E}[ \Vert {\textbf {R}}_{t,j-1} \Vert ]\big ). \end{aligned}$$

Applying these upper bounds for $t-j=t'-j'$ ( this latter condition on the indexes implies that both ${\textbf {R}}_{t,j}$ and ${\textbf {R}}_{t,j}$ are $\mathcal {F}_{t-j}$-measurable, i.e ${\textbf {R}}_{t,j} = \tilde{f}_1({\textbf {Z}}_{t-j-1}, {\textbf {Z}}_{t-j-2}, \ldots )$ and ${\textbf {R}}_{t',j'} = \tilde{f}_2({\textbf {Z}}_{t-j-1}, {\textbf {Z}}_{t-j-2}, \ldots )$ )

$$\begin{aligned}&\mathbb {E}\Big [ \left( h_{t,j}( {\textbf {x}}){\textbf {A}}_{j} {\textbf {Z}}_{t-j} \right) \left( h_{t',j'}( {\textbf {x}}) {\textbf {A}}_{j'} {\textbf {Z}}_{t'-j'}\right) \Big ] \\&\quad \le \mathbb {E}\left[ \Bigl | h_{t,j}( {\textbf {x}}) {\textbf {A}}_{j} {\textbf {Z}}_{t-j} \Bigr | \, \Bigl | h_{t',j'}( {\textbf {x}}) {\textbf {A}}_{j'} {\textbf {Z}}_{t'-j'} \Bigr | \right] \\&\quad \le C \, \Vert {\textbf {A}}_{j} \Vert \cdot \Vert {\textbf {A}}_{j'} \Vert \\&\qquad \mathbb {E}\Big [ \Vert {\textbf {Z}}_{t-j}\Vert ^2 \Vert {\textbf {Z}}_{t'-j'}\Vert ^2 \left( \Vert {\textbf {R}}_{t,j}\Vert +\mathbb {E}[ \Vert {\textbf {R}}_{t,j} \Vert ] \right) \cdot \left( \Vert {\textbf {R}}_{t',j'}\Vert +\mathbb {E}[ \Vert {\textbf {R}}_{t',j'} \Vert ]\right) \Big ]\\&\quad \le C \, \Vert {\textbf {A}}_{j} \Vert \cdot \Vert {\textbf {A}}_{j'} \Vert \\&\qquad \mathbb {E}\Big [ \Vert {\textbf {Z}}_{t-j}\Vert ^4\Big ] \mathbb {E}\Big [ \left( \Vert {\textbf {R}}_{t,j}\Vert +\mathbb {E}[ \Vert {\textbf {R}}_{t,j} \Vert ] \right) \cdot \left( \Vert {\textbf {R}}_{t',j'}\Vert +\mathbb {E}[ \Vert {\textbf {R}}_{t',j'} \Vert ]\right) \Big ] \quad \text {independence} \\&\quad \le C \, \Vert {\textbf {A}}_{j} \Vert \cdot \Vert {\textbf {A}}_{j'} \Vert \, \left( \mathbb {E}\Big [ \Vert {\textbf {R}}_{t,j}\Vert \cdot \Vert {\textbf {R}}_{t',j'}\Vert \Big ] +3\mathbb {E}[ \Vert {\textbf {R}}_{t,j} \Vert ]\cdot \mathbb {E}[ \Vert {\textbf {R}}_{t',j'} \Vert ] \right) \\&\quad \le C \, \Vert {\textbf {A}}_{j} \Vert \cdot \Vert {\textbf {A}}_{j'} \Vert \, 4 \sqrt{\mathbb {E}[ \Vert {\textbf {R}}_{t,j}\Vert ^2]}\sqrt{\mathbb {E}[ \Vert {\textbf {R}}_{t',j'}\Vert ^2]} \quad \text {Cauchy--Schwarz}. \end{aligned}$$

We know that the special structure of the coefficients of ${\textbf {X}}_t$ which ensures long-range dependent behavior, implies by Lemma , there exists $\zeta >0$ sufficiently small such that $\mathbb {E} \Vert {\textbf {R}}_{t,j} \Vert ^2 \le C j^{2d -1+\zeta }$, and therefore,

$$\begin{aligned} \mathbb {E}\Big [ h_{t,j}( {\textbf {x}}) {\textbf {A}}_{j} {\textbf {Z}}_{t-j} , h_{t',j'}( {\textbf {x}}) {\textbf {A}}_{j'} {\textbf {Z}}_{t'-j'} \Big ]&\le C \Vert {\textbf {A}}_{j}\Vert \cdot \Vert {\textbf {A}}_{j'} \Vert \sqrt{\mathbb {E}[ \Vert {\textbf {R}}_{t,j}\Vert ^2]} \sqrt{\mathbb {E}[ \Vert {\textbf {R}}_{t',j'}\Vert ^2]} \\&\le C \Vert {\textbf {A}}_{j} \Vert \cdot \Vert {\textbf {A}}_{j'} \Vert j^{d-1/2+\zeta /2} j'^{ d-1/2+\zeta /2} \\&\le C \Vert {\textbf {A}}_{j} \Vert \cdot \Vert {\textbf {A}}_{j'} \Vert (j j')^{d-1/2+\zeta /2}. \end{aligned}$$

Taking into account that $$j' = (t'-t)+j $$, and that $${\textbf {A}}_{j} \sim j^{d-1} {\textbf {A}}_{\infty }$$, we find

$$\begin{aligned} \mathop {\operatorname {Var}}(T_{n}^{(2)})&\le C \sum _{t=1}^{n} \sum _{t'=t}^{n} \sum _{j=1}^{\infty } \Vert {\textbf {A}}_{j}\Vert \cdot \Vert {\textbf {A}}_{j'}\Vert (j j')^{d-1/2+\zeta /2}\\&\le C \sum _{t=1}^{n} \sum _{t'=t}^{n} \sum _{j=1}^{\infty } (j((t'-t)+j))^{d-1+\zeta /2}(j((t'-t)+j))^{d-1/2+\zeta /2} \\&\le C n \sum _{t=1}^{n} \sum _{j=1}^{\infty } (j((n-t)+j))^{d-1+\zeta /2}(j((n-t)+j))^{d-1/2+\zeta /2} \\&= C n \sum _{t=0}^{n-1} \sum _{j=1}^\infty (j(t+j))^{d-1+\zeta /2}(j(t+j))^{d-1/2+\zeta /2} \\&\le C n \sum _{t=1}^n\sum _{j=1}^\infty (j(t+j))^{2d -3/2+\zeta }. \end{aligned}$$

For $d\in (0,1/4),$ we can choose $\zeta$ sufficiently small such that $\gamma := 3/2-2d-\zeta > 1$ and by Lemma 7,

$$\begin{aligned} n \sum _{t=1}^n \sum _{j=1}^\infty (j(t+j))^{2d -3/2+\zeta }\lesssim n\sum _{t=1}^n t^{-\gamma }< n\sum _{t=1}^\infty t^{-\gamma }< \mathcal {O}(n). \end{aligned}$$

For $d\in [1/4, 1/2),$ we can choose $\zeta$ sufficiently small such that $\gamma := 3/2-2d-\zeta \in (1/2,1)$ and by Lemma 7,

$$\begin{aligned} n \sum _{t=1}^n \sum _{j=1}^\infty (j(t+j))^{2d -3/2+\zeta }&\le n\sum _{t=1}^n t^{-2\gamma +1} \sim n\int _1^n t^{-2\gamma +1 }dt \\&= n \mathcal {O}(n^{2-2\gamma }) = \mathcal {O}(n^{4d+2\zeta }). \end{aligned}$$

To simplify the expression, we can set $\zeta '\leftarrow 2\zeta$, and rename $\zeta '$ as $\zeta$. $\square$

### Auxiliary results for the reduction principle

#### Lemma 5

Let ${\textbf {X}}_t=\sum _{j=0}^\infty {\textbf {A}}_{j} {\textbf {Z}}_{t-j}$ with ${\textbf {A}}_{j} \overset{j \rightarrow \infty }{\sim }\ j^{d-1}L(j)$ with $d\in (0,1/2)$, *L* is a continuous slowly varying matrix, and $({\textbf {Z}}_j)_{j \in \mathbb {Z}}\overset{i.i.d.}{\sim }\ \mathcal {W}\mathcal {N}({\textbf {0}},\varvec{\Sigma })$. For all $0<\zeta <|2d-1|$, the following upper bound for ${\textbf {R}}_{t,j}$ holds:

C21 $$\begin{aligned} \mathbb {E} \left[ \Vert {\textbf {R}}_{t,j} \Vert ^2 \right] \le C\, j^{2d -1+\zeta }, \end{aligned}$$

where $C>0$ is a constant.

#### Proof

In the following, we will denote with *C* a generic constant. Observe that $({\textbf {Z}}_j)_{j \in \mathbb {Z}}$ are orthogonal in $L^2(\Omega , \mathbb {P})$ with respect to the scalar product $\langle X,Y \rangle :=\mathbb {E}[XY]$ (note that $\mathbb {E}[{\textbf {Z}}_i]={\textbf {0}}$ for all *i*) because ${\textbf {Z}}_i$ are independent, and therefore, $\mathbb {E} \Vert {\textbf {R}}_{t,j} \Vert ^2 \le \sum _{i > j} \mathbb {E} \Vert {\textbf {A}}_{i} {\textbf {Z}}_{t-i} \Vert ^2$. Moreover, by the assumptions on the coefficients ${\textbf {A}}_{j}$ and boundedness of the variance of $\Vert {\textbf {Z}}_j\Vert$, we have

$$\begin{aligned} \mathbb {E} \Vert {\textbf {R}}_{t,j} \Vert ^2 \le \sum _{i> j} \mathbb {E} \Vert {\textbf {A}}_{i} {\textbf {Z}}_{t-i} \Vert ^2 \le C \sum _{i > j} i^{-2(1-d)} \Vert L(i)\Vert ^2. \end{aligned}$$

We would like to affirm that there exists a constant $C>0$, such that $\Vert L(i)\Vert ^2\le C i^{\zeta }$ for all $i\ge j$. Note two things:

1. $L(\cdot )$ is a matrix whose entries are slowly varying functions. By the principle of equivalence of norm on finite vector spaces, there exists $C>0$, such that $\Vert \cdot \Vert \le C \Vert \cdot \Vert _2$. Further, $\Vert \cdot \Vert _2 \le \Vert \cdot \Vert _F^2$, where $\Vert \cdot \Vert _F^2$ is the Frobenius norm, i.e $\Vert {\textbf {A}} \Vert _F^2 = \left( \sum _{hs}A_{hs}^2\right) ^{1/2}$. Thus,
  $$\begin{aligned} \Vert L(\cdot )\Vert ^2 \le C\sum _{h=1}^r\sum _{s=1}^r |L_{hs}(\cdot )|^2 \overset{\text {def}}{=}\mathcal {L}(\cdot ).\end{aligned}$$
2. $\mathcal {L}(\cdot )$ is slowly varying at infinity, since the square of a slowly varying function is a slowly varying function, and the sum of slowly varying functions is still slowly varying. Lastly, $\mathcal {L}$ is continuous, as composition of its continuous entries $L_{hs}$.

In conclusion, $\mathcal {L}(\cdot )$ is slowly varying real function. As a consequence of Karamata’s representation theorem (For instance Pipiras and Taqqu 2017, relation 2.2.3), if $\mathcal {L}(x)$ is a slowly varying function at infinity,

$$\begin{aligned} \lim _{x\rightarrow \infty } \mathcal {L}(x)x^{-\zeta } =0\quad \text {for all}\quad \zeta >0, \end{aligned}$$

or equivalently, for all $\varepsilon >0$ there exists $x_0 \in \mathbb {R}$ such that

$$\begin{aligned} \mathcal {L}(x) \le \varepsilon x^{\zeta }\quad \text {for}\quad x>x_0. \end{aligned}$$

It must be noticed that the we cannot yet conclude $$\Vert L(i)\Vert ^2\le C i^{\zeta }$$ for all $$i\ge j$$, but only for $$i\ge x_0$$. If $$x_0 \le j$$, we are done. So, we can assume $$j<x_0 $$. Note that the function $$\mathcal {L}(x) x^{-\zeta }$$ is continuous and in $$[0,x_0]\supset [j, x_0]$$, which is compact in $$\mathbb {R}$$. Therefore, there exists $$C_1$$ such that $$\mathcal {L}(x) x^{-\zeta }\le C_1$$, for all $$x\in [0,x_0].$$

Now we possess all the elements to conclude. Consider a fixed $\varepsilon >0$ and corresponding $x_0.$ Then,

$$\begin{aligned} \mathbb {E} \Vert {\textbf {R}}_{t,s} \Vert ^2&\le C \sum _{i> j} i^{-2(1-d)} \Vert L(i)\Vert ^2 \le C \sum _{i> j} i^{-2(1-d)} \mathcal {L}(i) \\&=\sum _{i>j}^{[x_0]} i^{-2(1-d)} \mathcal {L}(i) + \sum _{i>[x_0]} i^{-2(1-d)} \mathcal {L}(i) \\&\le \sum _{i>j}^{[x_0]} i^{-2(1-d)} i^{\zeta } i^{-\zeta } \mathcal {L}(i) + C_2\sum _{i>[x_0]} i^{-2(1-d) +\zeta }, \quad \text {for any}\quad C_2\ge \varepsilon \\&\le C_1 \sum _{i>j}^{[x_0]} i^{-2(1-d)+\zeta } + C_2\sum _{i>[x_0]} i^{-2(1-d) +\zeta } \\&\le \max \{C_1, C_2\} \sum _{i>j} i^{-2(1-d)+\zeta } = C \sum _{i>j} i^{-2(1-d)+\zeta }. \\ \end{aligned}$$

Note that the last *C* does not depend on *j*. Finally, the last sum is asymptotically equivalent to

$$\begin{aligned} \sum _{i > j} i^{-2(1-d)+\zeta } \sim \int _j^\infty x^{-2(1-d) +\zeta }dx =\frac{1}{2d-1+\zeta } x^{2d-1+\zeta }\Big |^\infty _j = C j ^{2d-1+\zeta }. \end{aligned}$$

In the last step, we used the fact that we chose $\zeta \in (0,|2d-1|)$; thus, $2d-1+\zeta < 0$. Finally,

$$\begin{aligned} \mathbb {E} \Vert {\textbf {R}}_{t,s} \Vert ^2 \le \sum _{i> j} \mathbb {E} \Vert {\textbf {A}}_{i} {\textbf {Z}}_{t-i} \Vert ^2 \le C^2 \sum _{i > j} i^{-2(1-d)+\zeta } \le C^2 j^{2d-1+\zeta }. \end{aligned}$$

$$\square $$

#### Lemma 6

Let $\mathcal {F}_{t-j}=\sigma \left( {\textbf {Z}}_{t-j}, {\textbf {Z}}_{t-j-1}, \ldots \right)$ so that $\mathcal {F}_{t-(j+1)} \subset \mathcal {F}_{t-j} \subset \cdots \subset \mathcal {F}_{t-1}\subset \mathcal {F}_{t}.$ Consider ${\textbf {X}}_t=\sum _{j=0}^\infty {\textbf {A}}_{j} {\textbf {Z}}_{t-j}$, where ${\textbf {A}}_{j} \in \mathbb {R}^{r\times r}$. Then, for all ${\textbf {x}}\in \mathbb {R}^r$ the following hold

(i) $$\mathbbm {1}\left( \{{\textbf {X}}_t\le {\textbf {x}}\}\right) = \mathbb {P}( {\textbf {X}}_t \le {\textbf {x}}| \mathcal {F}_{t}),$$
(ii) $$\mathbb {P}( {\textbf {X}}_t \le {\textbf {x}}) \overset{a.s}{=}\lim _{j\rightarrow \infty }\mathbb {P}\left( {\textbf {X}}_t \le {\textbf {x}}| \, \mathcal {F}_{t-j}\right) ,$$
(iii) $$\mathbb {P}\left( {\textbf {X}}_t \le {\textbf {x}} | \, \mathcal {F}_{t-j}\right) =p_j( {\textbf {x}} - {\textbf {R}}_{t,j}).$$

#### Proof

Let $K_{-j}=\mathbb {E}[ \mathbbm {1}\left( \{{\textbf {X}}_t\le {\textbf {x}}\}\right) |\mathcal {F}_{t-j}]=\mathbb {P}( {\textbf {X}}_t \le {\textbf {x}}| \mathcal {F}_{t-j})$ for all $j \in \mathbb {N}$.

(i) ${\textbf {X}}_t$ is $\mathcal {F}_{t}$ measurable, and the previous theorem yields
  $$\begin{aligned} \mathbb {P}( {\textbf {X}}_t \le {\textbf {x}}| \mathcal {F}_{t}) = \mathbb {E}\left[ \mathbbm {1}\left( \{{\textbf {X}}_t\le {\textbf {x}}\}\right) |\mathcal {F}_{t} \right] =\mathbbm {1} \left( \{{\textbf {X}}_t\le {\textbf {x}}\}\right) =K_0.\end{aligned}$$
(ii) It is clear that $K_{-j}$ is $\mathcal {F}_{t-j}$- measurable and integrable. Also, for $j\ge i$ integer,
  $$\begin{aligned} \mathbb {E}[K_{-i}|\mathcal {F}_{t-j}]&= \mathbb {E}[\mathbb {E}[ \mathbbm {1}\left( \{{\textbf {X}}_t\le {\textbf {x}}\}\right) |\mathcal {F}_{t-i}]|\mathcal {F}_{t-j}]\\&\underbrace{=}_{\mathcal {F}_{t-j}\subset \mathcal {F}_{t-i}} \mathbb {E}[ \mathbbm {1}\left( \{{\textbf {X}}_t\le {\textbf {x}}\}\right) |\mathcal {F}_{t-j}]=K_{-j}. \end{aligned}$$
  Therefore, $(K_{-j})_{j\in \mathbb {N}}$ is a backwards martingale with respect to $(\mathcal {F}_{t-j})_{j\in \mathbb {N}}$. Note that we applied the tower property to the conditional expectation. Using the backwards martingale convergence theorem (for instance Jacod and Protter 2012, p. 233, Theorem 27.4), $K_{-j}:= \mathbb {P}( {\textbf {X}}_t \le {\textbf {x}}| \mathcal {F}_{t-j})$ converges almost surely and in probability to a limit $K_{-\infty }$ that is $\mathcal {F}_{t}= \bigcap _{i=0}^\infty \mathcal {F}_{t-i}$ measurable. By 1), the limit is $\mathbb {P}( {\textbf {X}}_t \le {\textbf {x}}| \mathcal {F}_{t})=\mathbb {P}( {\textbf {X}}_t \le {\textbf {x}})$.
(iii) Note that $\mathbb {P}( {\textbf {X}}_t \le {\textbf {x}}|\mathcal {F}_{t}) = \mathbb {P}( {\textbf {X}}_{t,j} \le {\textbf {x}}-{\textbf {R}}_{t,j} | \mathcal {F}_{t})=p_j({\textbf {x}}-{\textbf {R}}_{t,j} ).$

$$\square $$

#### Lemma 7

(Lemma 6.5 in Ho and Hsing 1996) Given a constant $\gamma >1/2$, $l\ge 1$, there exists $C <\infty$ such that, for all $l \ge 1$, we have

$$\begin{aligned} \sum _{j=1}^{\infty } (j(l+j))^{-\gamma } \le {\left\{ \begin{array}{ll} C l^{-2\gamma +1},& \gamma \in (1/2, 1) \\ C \frac{\log l}{l},& \gamma = 1\\ C l^{-\gamma }, & \gamma > 1. \end{array}\right. } \end{aligned}$$

#### Lemma 8

(Lemma 6.4 in Ho and Hsing 1996) For all ${\textbf {x}}$, ${\textbf {x'}} \in \mathbb {R}^{r}$, and $t,t',j,j'\ge 1$ such that $t'-j'\ne t-j$,

(i) $$\begin{aligned}&\mathbb {E}[U_{t,j}({\textbf {x}})U_{t',j'}({\textbf {x}})]\\&=\mathop {\operatorname {Cov}}\left( p_{j-1}( {\textbf {x}} - {\textbf {R}}_{t,j-1}) - p_{j}( {\textbf {x}} - {\textbf {R}}_{t, j} ) ,p_{j'-1}( {\textbf {x}} - {\textbf {R}}_{t', j'-1})\right. \\&\quad \left. - p_{j'}( {\textbf {x}} - {\textbf {R}}_{t', j'} ) \, \right) =0. \end{aligned}$$
(ii) $$\mathop {\operatorname {Cov}}\left( p_{j-1}( {\textbf {x}} - {\textbf {R}}_{t, j-1}) - p_{j}( {\textbf {x}} -{\textbf {R}}_{t, j} ), \nabla p_{j'-1}( {\textbf {x'}} - {\textbf {R}}_{t', j'-1}) {\textbf {Z}}_{t'-j'}\right) =0.$$

#### Proof

(i) Let $\mathcal {F}_j= \sigma ( Z_i \, | \, i<j)$. Notice that from the third point of Lemma 6,

$$\begin{aligned} \mathbb {E}[\mathbbm {1} \left( \{{\textbf {X}}_t \le {\textbf {x}}\}\right) | \mathcal {F}_{t-j}] = \mathbb {P}( {\textbf {X}}_t \le {\textbf {x}} | \mathcal {F}_{t-j}) = p_{j}( {\textbf {x}} - {\textbf {R}}_{t,j}). \end{aligned}$$

Next, we rename $U_t({\textbf {x}})=\mathbbm {1}\left( \{{\textbf {X}}_t \le {\textbf {x}}\}\right) - p({\textbf {x}})$ and let $U_{t,j}({\textbf {x}})$ be the quantity

$$\begin{aligned} U_{t,j}({\textbf {x}})&= p_{j-1}( {\textbf {x}} - {\textbf {R}}_{t,j-1}) -p({\textbf {x}}) + p({\textbf {x}}) - p_{j}( {\textbf {x}} - {\textbf {R}}_{t,j} )\\&=\mathbb {E}[U_t({\textbf {x}})| \mathcal {F}_{t-j+1}] - \mathbb {E}[U_t({\textbf {x}})| \mathcal {F}_{t-j}]. \end{aligned}$$

Our goal is to prove that $\mathbb {E}[U_{t,j}({\textbf {x}})U_{t',j'}({\textbf {x}}) ]=0$. Since by assumption $t'-j'\ne t-j$, without loss of generality we can assume $t'-j'< t-j$. Then, $\mathcal {F}_{t'-j'}\subset \mathcal {F}_{t-j}$. Moreover, $\mathbb {E}[U_t({\textbf {x}})| \mathcal {F}_{t-j}]$ is $\mathcal {F}_{t-j}$ measurable.

$$\begin{aligned}&\mathbb {E}[U_{t,j}({\textbf {x}})U_{t',j'}({\textbf {x}})] \\&\quad = \mathbb {E}\Big [U_{t',j'} ({\textbf {x}})\Big (\mathbb {E}[U_t({\textbf {x}})| \mathcal {F}_{t-j+1}] - \mathbb {E}[U_t({\textbf {x}})| \mathcal {F}_{t-j}]\Big ) \Big ]\quad \text {def of }U_{t,j}\\&\quad = \mathbb {E}\left[ \mathbb {E}\Big [U_{t',j'}({\textbf {x}})\Big (\mathbb {E}[U_t({\textbf {x}})| \mathcal {F}_{t-j+1}] - \mathbb {E}[U_t({\textbf {x}})| \mathcal {F}_{t-j}]\Big ) \Big | \mathcal {F}_{t'-j'}\right] \Big ] \quad \text {Tower property}\\&\quad = \mathbb {E}\left[ U_{t',j'}({\textbf {x}})\mathbb {E}\Big [ \Big (\mathbb {E}[U_t({\textbf {x}})| \mathcal {F}_{t-j+1}] - \mathbb {E}[U_t({\textbf {x}})| \mathcal {F}_{t-j}]\Big ) \Big | \mathcal {F}_{t'-j'}\right] \Big ] \\&\qquad U_{t'-j'} \text { is }\mathcal {F}_{t'-j'}\text {-measurable}\\&\quad = \mathbb {E}\left[ U_{t',j'}({\textbf {x}})\Big (\mathbb {E}\Big [ \mathbb {E}[U_t({\textbf {x}})| \mathcal {F}_{t-j+1}]\Big | \mathcal {F}_{t'-j'}\right] - \mathbb {E}\left[ \mathbb {E}[U_t({\textbf {x}})| \mathcal {F}_{t-j}] \Big | \mathcal {F}_{t'-j'}\right] \Big )\Big ] \\&\quad =\mathbb {E}\Big [U_{t',j'}({\textbf {x}})\Big (\mathbb {E}[U_t({\textbf {x}})| \mathcal {F}_{t'-j'}] - \mathbb {E}[U_t({\textbf {x}})| \mathcal {F}_{t'-j'}]\Big ) \Big ]=0,\\&\qquad \mathcal {F}_{t'-j'}\subset \mathcal {F}_{t-j}\subset \mathcal {F}_{t-j+1}.\\ \end{aligned}$$

(ii) can be proved similarly since $\nabla p_{j'-1}( {\textbf {x'}} - {{\textbf {R}}}_{t', j'-1}) {\textbf {Z}}_{t'-j'}$ is $\mathcal {F}_{t'-j'}$ - measurable. $\square$

#### Lemma 9

Suppose the assumptions of Theorem 10 are satisfied with integer *r*. Let $K_{t,j}$ be defined as $K_{t,j}({\textbf {x}}) := p_{j-1}({\textbf {x}} - {\textbf {R}}_{t,j-1}) - p_{j}({\textbf {x}} - {\textbf {R}}_{t,j}) + \mathbbm {1}_{\{j\ge r\}} \nabla p_{j-1}({\textbf {x}} - {\textbf {R}}_{t,j}) {\textbf {A}}_{j} {\textbf {Z}}_{t-j}.$ Then, there exist two positive a constants $C_1$ and $C_2$ such that, for any $j \ge r$ and any $t \in \mathbb {N}$,

$$\begin{aligned} | K_{t,j}({\textbf {x}}) | \le C_1\Vert {\textbf {A}}_{j} \Vert ^2 \left( \Vert {\textbf {Z}}_{t-j} \Vert ^2 +C_2 \right) . \end{aligned}$$

#### Proof of Lemma 9

Suppose that relationship (C22) below holds for all $j \ge r$, where $\delta _j({\textbf {t}}) = \lambda ^*{\textbf {A}}_{j} ({\textbf {Z}}_{t-j} - {\textbf {t}})$, with $\lambda ^*\in (0,1)$, and $\eta _j = \mu ^*{\textbf {A}}_{j} {\textbf {Z}}_{t-j}$, with $\mu ^*\in (0,1)$:

C22 $$\begin{aligned}&K_{t,j}({\textbf {x}})= \left\langle \nabla ^2 p_{j-1}( {\textbf {x}} - {\textbf {R}}_{t,j-1} + \eta _{t,j} ) {\textbf {A}}_{j} {\textbf {Z}}_{t-j},{\textbf {A}}_{j} {\textbf {Z}}_{t-j} \right\rangle \end{aligned}$$

C23 $$\begin{aligned}&- \frac{1}{2} \int \left\langle \nabla ^2 p_{j-1} \Big ( {\textbf {x}} -{\textbf {R}}_{t,j-1} + \delta _{t,j}({\textbf {t}}) \Big ) {\textbf {A}}_{j}( {\textbf {Z}}_{t-j} - {\textbf {t}} ), {\textbf {A}}_{j}( {\textbf {Z}}_{t-j} - {\textbf {t}} ) \right\rangle \, d\mathcal {G}({\textbf {t}}). \end{aligned}$$

Then, it follows

$$\begin{aligned} | K_{t,j}({\textbf {x}}) |&\le \Bigl \Vert \nabla ^2 p_{j-1}({\textbf {x}}- {\textbf {R}}_{t,j-1} + \eta _j ) \Bigr \Vert \cdot \Bigl \Vert {\textbf {A}}_{j} {\textbf {Z}}_{t-j} \Bigr \Vert ^2 \\&\quad + \int \Bigl \Vert \nabla ^2 p_{j-1}({\textbf {x}} - {\textbf {R}}_{t,j-1} + \delta _{j}({\textbf {t}})) \Bigr \Vert \cdot \Bigl \Vert {\textbf {A}}_{j}( {\textbf {Z}}_{t-j} - {\textbf {t}} ) \Bigr \Vert ^2 \, d\mathcal {G}({\textbf {t}})\nonumber \\&\le C_1' \Vert {\textbf {A}}_{j}\Vert ^2 \Vert {\textbf {Z}}_{t-j}\Vert ^2 + C_2'\Vert {\textbf {A}}_{j}\Vert ^2 \left( \Vert {\textbf {Z}}_{t-j}\Vert ^2 +\int \Vert {\textbf {t}}\Vert ^2 \, d\mathcal {G}({\textbf {t}}) \right) \\&\le C_1\Vert {\textbf {A}}_{j} \Vert ^2 \left( \Vert {\textbf {Z}}_{t-j} \Vert ^2 +C_2 \right) . \end{aligned}$$

We used fact that the partial derivatives of $p_j$ are uniformly bounded for every *j* by assumption (C18) and the assumed finite second moment of ${\textbf {Z}}_1$. It remains to prove (C22). By (A3), we obtain

C24 $$\begin{aligned} p_s({\textbf {x}})= &  \mathbb {P}({\textbf {X}}_{t,s} \le {\textbf {x}} )\nonumber \\= &  \mathbb {P}({\textbf {X}}_{t,s-1} + A_s {\textbf {Z}}_{t-s} \le {\textbf {x}} ) =\int p_{s-1} ({\textbf {x}}- A_s{\textbf {t}} ) \, d\mathcal {G}({\textbf {t}}). \end{aligned}$$

Via Equation (C24), we can write

$$\begin{aligned}&p_{j-1}({\textbf {x}} -{\textbf {R}}_{t,j-1}) - p_{j}({\textbf {x}} -{\textbf {R}}_{t,j}) \\&\quad = \int \Big ( p_{j-1}({\textbf {x}} -{\textbf {R}}_{t,j-1}) - p_{j-1} ({\textbf {x}} -{\textbf {R}}_{t,j}- {\textbf {A}}_{j} {\textbf {t}} ) \Big ) \, d\mathcal {G}({\textbf {t}}) \\&\quad = \int \Big ( p_{j-1}({\textbf {x}} -{\textbf {R}}_{t,j-1}) - p_{j-1} ({\textbf {x}} -{\textbf {R}}_{t,j}-{\textbf {A}}_{j} {\textbf {Z}}_{t-j}+{\textbf {A}}_{j} {\textbf {Z}}_{t-j} - {\textbf {A}}_{j} {\textbf {t}} ) \Big ) \, d\mathcal {G}({\textbf {t}}) \\&\quad = \int \Big ( p_{j-1}({\textbf {x}} -{\textbf {R}}_{t,j-1}) - p_{j-1} ({\textbf {x}} -{\textbf {R}}_{t,j-1}+{\textbf {A}}_{j}({\textbf {Z}}_{t-j}- {\textbf {t}}) ) \Big ) \, d\mathcal {G}({\textbf {t}}). \\ \end{aligned}$$

We consider the real-valued function

$$\begin{aligned} g(\lambda ) : = \int \Big ( p_{j-1}( {\textbf {x}} -{\textbf {R}}_{t,j-1}) - p_{j-1} ( {\textbf {x}} -{\textbf {R}}_{t,j-1} + \lambda {\textbf {A}}_{j}( {\textbf {Z}}_{t-j} - {\textbf {t}} )) \Big ) \, d\mathcal {G}({\textbf {t}}). \end{aligned}$$

Next, we compute the derivatives of *g*. By assumption, the function ${\textbf {t}}\mapsto p_{j-1} ({\textbf {x}} -{\textbf {R}}_{t,j}- {\textbf {A}}_{j} {\textbf {t}} )$ and its first derivatives are integrable with respect to the measure $\mathcal {G}({\textbf {t}})$ by assumption for all $j\ge r$. Therefore, we can use the dominated convergence theorem, and by taking derivatives with respect to $\lambda$, we obtain

$$\begin{aligned} g'(\lambda )&= - \int \nabla p_{j-1} \Big ( {\textbf {x}} -{\textbf {R}}_{t,j-1}+ \lambda {\textbf {A}}_{j}( {\textbf {Z}}_{t-j} - {\textbf {t}} )\Big ) {\textbf {A}}_{j}( {\textbf {Z}}_{t-j} - {\textbf {t}} ) \, d\mathcal {G}({\textbf {t}}), \\ g''(\lambda )&= - \frac{1}{2} \int \Bigg \langle \nabla ^2 p_{j-1} \Big ( {\textbf {x}} -{\textbf {R}}_{t,j-1}+ \lambda {\textbf {A}}_{j}( {\textbf {Z}}_{t-j} - {\textbf {t}} )\Big ) \\&\quad {\textbf {A}}_{j}( {\textbf {Z}}_{t-j} - {\textbf {t}} ) ,{\textbf {A}}_{j}( {\textbf {Z}}_{t-j} - {\textbf {t}} ) \Bigg \rangle \, d\mathcal {G}({\textbf {t}}). \end{aligned}$$

Moreover, since $g \in C^2([0,1])$, the mean value theorem yields, $g(1) = g(0) + g'(0) + \frac{1}{2} g''(\lambda ^*)$ for some value $\lambda ^*\in (0,1)$ yields

$$\begin{aligned}&p_{j-1}( {\textbf {x}} -{\textbf {R}}_{t,j-1}) - p_{j}( {\textbf {x}} -{\textbf {R}}_{t,j}) \\&\quad = 0 - \int \nabla p_{j-1} \Big ( {\textbf {x}} -{\textbf {R}}_{t,j-1} \Big ) {\textbf {A}}_{j}( {\textbf {Z}}_{t-j} - {\textbf {t}} ) \, d\mathcal {G}({\textbf {t}}) \\&\qquad - \frac{1}{2} \int \Bigg \langle \nabla ^2 p_{j-1} \Big ( {\textbf {x}} -{\textbf {R}}_{t,j-1}+ \lambda ^*{\textbf {A}}_{j}( {\textbf {Z}}_{t-j} - {\textbf {t}} )\Big ) {\textbf {A}}_{j}( {\textbf {Z}}_{t-j} - {\textbf {t}} ),\\&\qquad {\textbf {A}}_{j}( {\textbf {Z}}_{t-j} - {\textbf {t}} ) \Bigg \rangle \, d\mathcal {G}({\textbf {t}}) \\&\quad = - \nabla p_{j-1} \Big ( {\textbf {x}} -{\textbf {R}}_{t,j-1} \Big ) {\textbf {A}}_{j} {\textbf {Z}}_{t-j} \\&\qquad - \frac{1}{2} \int \Bigg \langle \nabla ^2 p_{j-1} \Big ( {\textbf {x}} -{\textbf {R}}_{t,j-1}+ \lambda ^*{\textbf {A}}_{j}( {\textbf {Z}}_{t-j} - {\textbf {t}} )\Big ) \\&\qquad {\textbf {A}}_{j}( {\textbf {Z}}_{t-j} - {\textbf {t}} ),{\textbf {A}}_{j}( {\textbf {Z}}_{t-j} - {\textbf {t}} ) \Bigg \rangle \, d\mathcal {G}({\textbf {t}}), \end{aligned}$$

using $\mathbb {E}[{\textbf {Z}}_{t-j}]= 0$ for the second equality. However, the first-order term of the approximation is $- \nabla p_{j-1} \Big ( {\textbf {x}} -{\textbf {R}}_{t,j-1} \Big ) {\textbf {A}}_{j} {\textbf {Z}}_{t-j}$, whereas we want to obtain $- \nabla p_{j-1} \Big ( {\textbf {x}} -{\textbf {R}}_{t,j} \Big ) {\textbf {A}}_{j} {\textbf {Z}}_{t-j}$. This latter term shall appear in another Taylor decomposition. In other words, we consider

$$\begin{aligned} \phi (\mu ):= \nabla p_{j-1}( {\textbf {x}} -{\textbf {R}}_{t,j-1}) - \nabla p_{j-1}( {\textbf {x}} -{\textbf {R}}_{t,j-1} + \mu {\textbf {A}}_{j} {\textbf {Z}}_{t-j} ). \end{aligned}$$

There exists $\mu ^*$ such that $\phi (1) = \phi (0) + \phi '(\mu ^*)$

$$\begin{aligned}&\Big [ \nabla p_{j-1}( {\textbf {x}} -{\textbf {R}}_{t,j-1}) - \nabla p_{j-1} ( {\textbf {x}} -{\textbf {R}}_{t,j})\Big ]{\textbf {A}}_{j} {\textbf {Z}}_{t-j} \\&\quad =- \left\langle \nabla ^2 p_{j-1}( {\textbf {x}} - {\textbf {R}}_{t,j-1} + \mu ^*{\textbf {A}}_{j} {\textbf {Z}}_{t-j} ) {\textbf {A}}_{j} {\textbf {Z}}_{t-j} , {\textbf {A}}_{j} {\textbf {Z}}_{t-j} \right\rangle . \end{aligned}$$

Re-arranging this last expression yields

$$\begin{aligned}&- \nabla p_{j-1}( {\textbf {x}} -{\textbf {R}}_{t,j-1}){\textbf {A}}_{j} {\textbf {Z}}_{t-j} =\\&- \nabla p_{j-1}( {\textbf {x}} -{\textbf {R}}_{t,j}){\textbf {A}}_{j} {\textbf {Z}}_{t-j}\\&+\left\langle \nabla ^2 p_{j-1}( {\textbf {x}} - {\textbf {R}}_{t,j-1}\!+\!\mu ^*{\textbf {A}}_{j} {\textbf {Z}}_{t-j} ) {\textbf {A}}_{j} {\textbf {Z}}_{t-j} , {\textbf {A}}_{j} {\textbf {Z}}_{t-j} \right\rangle . \\ \end{aligned}$$

Now we can simplify the notation by writing

$$\begin{aligned} {\left\{ \begin{array}{ll} \eta _{t,j} = \mu ^*{\textbf {A}}_{j} {\textbf {Z}}_{t-j}, \\ \delta _{t, j}({\textbf {t}}) = \lambda ^*{\textbf {A}}_{j}( {\textbf {Z}}_{t-j} - {\textbf {t}} ); \end{array}\right. } \end{aligned}$$

thus,

$$\begin{aligned} K_{t,j}( {\textbf {x}})&= p_{j-1}( {\textbf {x}} -{\textbf {R}}_{t,j-1}) - p_{j}( {\textbf {x}} -{\textbf {R}}_{t,j}) + \nabla p_{j-1} \Big ( {\textbf {x}} -{\textbf {R}}_{t,j} \Big ) {\textbf {A}}_{j} {\textbf {Z}}_{t-j}\\&= \nabla ^2 p_{j-1}( {\textbf {x}} - {\textbf {R}}_{t,j-1} + \eta _{t,j} ) ({\textbf {A}}_{j} {\textbf {Z}}_{t-j})^2 \\&\quad - \frac{1}{2} \int \Bigg \langle \nabla ^2 p_{j-1} \Big ( {\textbf {x}} -{\textbf {R}}_{t,j-1} + \delta _{t,j}({\textbf {t}}) \Big ) \\&\quad {\textbf {A}}_{j}( {\textbf {Z}}_{t-j} - {\textbf {t}} ) , {\textbf {A}}_{j}( {\textbf {Z}}_{t-j} - {\textbf {t}} ) \Bigg \rangle \, d\mathcal {G}({\textbf {t}}). \end{aligned}$$

$$\square $$

## Proofs for the turning rate

In order to prove Theorem 5 we will need the two following auxiliary results.

### Lemma 10

Let $\xi _0, \ldots , \xi _{n+1}$ be time series data whose increments $X_1,\ldots , X_{n+1}$ satisfy the linear representation $X_t=\sum _{j=0}^\infty a_j Z_{t-j}$ with $\sum _{j=0}^\infty |a_j|< \infty$ and $(Z_j)_{j \in \mathbb {Z}}$ forming an i.i.d sequence with $Z_1$ admitting a continuous and bounded density and finite second moment $\mathbb {E}[|Z_1|^2]<\infty$. Let $\mathcal {T}\subset \mathcal {S}_r$. Then, as $n\rightarrow \infty$

$$\begin{aligned} \frac{1}{\sqrt{n}}\sum _{t=1}^{\lfloor n \tau \rfloor } \sum _{\gamma \in \mathcal {T}} \left( \mathbbm {1}(\Pi (\xi _{t}, \dots , \xi _{t+r})=\gamma )-p(\gamma ) \right) \overset{\mathcal {D}[0,1]}{\Longrightarrow }\ \, \sigma B(\tau ), \text {\ for } \ \tau \in [0,1], \end{aligned}$$

with variance

$$\begin{aligned} \sigma ^2&= \mathop {\operatorname {Var}}\left( \sum _{\gamma \in \mathcal {T}} \mathbbm {1}\left( {\textbf {V}}_\gamma {\textbf {X}}_1 \le {\textbf {0}} \right) \right) \\&+2 \sum _{j=1}^\infty \mathop {\operatorname {Cov}}\left( \sum _{\gamma \in \mathcal {T}} \mathbbm {1}\left( {\textbf {V}}_\gamma {\textbf {X}}_1 \le {\textbf {0}} \right) , \sum _{\gamma \in \mathcal {T}} \mathbbm {1}\left( {\textbf {V}}_\gamma {\textbf {X}}_{1+j} \le {\textbf {0}} \right) \right) . \end{aligned}$$

where the matrices ${\textbf {V}}_\gamma$ are defined in (12).

### Proof

The proof extends the proof of Theorem 3.

The martingale decomposition (2) still holds with

D25 $$\begin{aligned} h({\textbf {X}}_t):&= \sum _{\gamma \in \mathcal {T}} \left( \mathbbm {1}(\Pi (\xi _{t}, \dots , \xi _{t+r})=\gamma )-p(\gamma ) \right) \end{aligned}$$

D26 $$\begin{aligned}&=\sum _{\gamma \in \mathcal {T}} \left( \mathbbm {1}\left( {\textbf {V}}_\gamma {\textbf {X}}_t \le {\textbf {0}} \right) - p(\gamma ) \right) = \sum _{j=0}^\infty \sum _{\gamma \in \mathcal {T}} \tilde{U}_{t,j, \gamma } = \sum _{j=0}^\infty \tilde{U}_{t,j}, \end{aligned}$$

where

D27 $$\begin{aligned} \tilde{U}_{t,j, \gamma }&:= \mathbb {E}\big [\mathbbm {1}( {\textbf {V}}_\gamma {\textbf {X}}_t\le {\textbf {0}}) \big | \mathcal {F}_{t-j}\big ] - \mathbb {E}\big [\mathbbm {1}({\textbf {V}}_\gamma {\textbf {X}}_t\le {\textbf {0}}) \big | \mathcal {F}_{t-j-1}\big ]\nonumber \\&=\tilde{p}_{j}(- {\textbf {R}}_{t,j}) - \tilde{p}_{j+1}( -{\textbf {R}}_{t,j+1}) \end{aligned}$$

and we set $\tilde{p}_j ({\textbf {x}}):= p_j({\textbf {V}}_\gamma {\textbf {x}}).$ From the Lipschitz continuity of $p_j$ (Lemma 3), it follows that $\tilde{p}_j$ is Lipschitz as well, with Lipschitz constant $\text {Lip}(\tilde{p}_j)\le \text {Lip}(p_j)\Vert {\textbf {V}}_\gamma \Vert \;.$ Therefore, the conclusion of Lemma 4 also holds for $\tilde{U}_{t,j}$, that is, for $j\ge J$

$$\begin{aligned} |\tilde{U}_{t,j}|&\le \sum _{\gamma \in \mathcal {T}} |\tilde{U}_{t,j, \gamma } | \le \sum _{\gamma \in \mathcal {T}} \text {Lip}(p_j)\Vert {\textbf {V}}_\gamma \Vert \, \Vert {\textbf {A}}_{j+1}\Vert \\&\quad \left( \Vert {\textbf {Z}}_{t-j-1}\Vert + \sqrt{\mathbb {E}[ \Vert {\textbf {Z}}_{t-j-1}\Vert ^2]} \right) \quad \text {see Lemma~4}\\&\le C \left( \sum _{\gamma \in \mathcal {T}} \Vert {\textbf {V}}_\gamma \Vert \right) \text {Lip}(p_J) \Vert {\textbf {A}}_{j+1}\Vert \, \left( \Vert {\textbf {Z}}_{t-j-1}\Vert + \sqrt{\mathbb {E}[ \Vert {\textbf {Z}}_{t-j-1}\Vert ^2]} \right) \;. \end{aligned}$$

Lastly, Lemma 8 applies to $\tilde{U}_{t,j}$, that is, $\mathbb {E}[ \tilde{U}_{t,j} U_{t',j'}]=0$ if $t-j \ne t'-j'.$ In fact,

$$\begin{aligned} \tilde{U}_{t,j}=\mathbb {E}[ h({\textbf {X}}_t)\,|\, \mathcal {F}_{t-j}] - \mathbb {E}[ h({\textbf {X}}_t)\,|\, \mathcal {F}_{t-j-1}], \end{aligned}$$

and the conclusion of Lemma 8 follows with $h({\textbf {X}}_t)$ replacing $U_t({\textbf {x}}).$ Finally, since the same conclusions of Lemmas 8 and 4 hold for $\tilde{U}_{t,j}$, we can redo exactly the same steps of the proof of Theorem 1 (i.e., the application of Theorem 4.2 of Billingsley) to

$$\begin{aligned} \tilde{W}_n:&=\frac{1}{\sqrt{n}}\sum _{t=1}^{\lfloor n \tau \rfloor } \sum _{j=0}^\infty \tilde{U}_{t,j}, \quad \tilde{V}_{u,n}:=\frac{1}{\sqrt{n}} \sum _{t=1}^{\lfloor n \tau \rfloor } \sum _{j=0}^{u-1} \tilde{U}_{t,j}, \\ \tilde{H}_{u,n}:&=\frac{1}{\sqrt{n}}\sum _{j=0}^{u-1} \left( \sum _{t=1}^j \tilde{U}_{t,j} - \sum _{t=\lfloor n \tau \rfloor +1}^{\lfloor n \tau \rfloor +j} \tilde{U}_{t,j}\right) \;. \end{aligned}$$

$$\square $$

### Remark 5

For $\tau =1$ and $\mathcal {T}=\{ (0,2,1),(2,0,1),(1,2,0),(0,2,1)\}$, we retrieve

$$\begin{aligned} \sqrt{n}\left( q_n -q \right) \xrightarrow {\mathcal {D}} \mathcal {N}(0,\sigma ^2). \end{aligned}$$

### Theorem 11

$\xi _0, \ldots , \xi _{n+1}$ be a time series. Consider the turning rate series generated by blocks of size $m+2$ and $n_b = \lfloor n/m \rfloor$ blocks, denoted by $\hat{q}_{1,m}, \ldots , \hat{q}_{n_b,m}$. Suppose $\frac{m}{\sqrt{n}}\longrightarrow \infty \;.$

(i) If $X_t = \sum _{j=0}^\infty a_j Z_{t-j}$ satisfies the assumptions on the increments of Theorem 3, then
  D28 $$\begin{aligned} \frac{m}{\sqrt{n}}\sum \limits _{j=1}^{\lfloor n_b \tau \rfloor } (\hat{q}_{j,m} -q)&\overset{\mathcal {D}[0,1]}{\Longrightarrow }\ &\sigma B(\tau )\quad \text {for}\quad \tau \in [0,1] \quad \text { as } n_b\rightarrow \infty \nonumber \\ &  (\text {or }n\rightarrow \infty ). \end{aligned}$$
  where $\sigma ^2$ is given in Lemma 10
(ii) If $X_t = \sum _{j=0}^\infty a_j Z_{t-j}$ satisfies the assumptions on the increments of Theorem 4, then
  D29 $$\begin{aligned} \frac{m}{n^{1/2+d}}\sum \limits _{j=1}^{n_b} (\hat{q}_{j,m} -q) \overset{\mathcal {D}}{\longrightarrow }\ \mathcal {N}(0,\sigma ^2)\quad \text { as } n_b\rightarrow \infty \quad (\text {or }n\rightarrow \infty ). \end{aligned}$$
  where $\sigma ^2= \sum _{\gamma _1, \gamma _2 \in \mathcal {T}} (\nabla p_{\gamma _2}({\textbf {0}}))^\top V_{\gamma _1}V V_{\gamma _2}^\top \nabla p_{\gamma _1}({\textbf {0}}),$ with $V= \frac{\Gamma (d)^2 }{\Gamma (2d+2) \cos \left( \pi d \right) } {\textbf {E}}$ and ${\textbf {V}}_\gamma$ the matrices defined in (24).

### Proof of Theorem 11

We set $$ h({\textbf {X}}_t):= \sum _{\gamma \in \mathcal {T}} \mathbbm {1}\left( {\textbf {V}}_{\gamma }{\textbf {X}}_t \le {\textbf {0}} \right) -q\;.$$

(i) Using (25),
  $$\begin{aligned} m(\hat{q}_{j,m} -q)&=\!\!\sum _{i=0}^{m-1} \sum _{\gamma \in \mathcal {T}} \mathbbm {1}\left( \Pi (\xi _{(j-1)(m+2)+i} , \xi _{(j-1)(m+2) +i+1}, \xi _{(j-1)(m+2)+i+2} )\!=\!\gamma \right) \\&\quad -mq \\&= \sum _{i=1}^{m} h\left( X_{(j-1)(m+2)+i},X_{(j-1)(m+2)+i+1}\right) \;. \end{aligned}$$
  Therefore,
  $$\begin{aligned} m\sum \limits _{j=1}^{\lfloor n_b \tau \rfloor } (\hat{q}_{j,m}-q)= \sum \limits _{j=1}^{\lfloor n_b\tau \rfloor }\sum _{i=1}^{m} h\left( X_{(j-1)(m+2)+i},X_{(j-1)(m+2)+i+1}\right) \;. \end{aligned}$$
  Further, we can unroll the previous expression: $\sum _{j=1}^{\lfloor n_b\tau \rfloor }\left( \hat{q}_{j,m}-q \right)$ is the sum of consecutive terms of the form $h(X_k, X_{k+1})$, but the summands $h(X_{jm+2j-1}, X_{jm+2j})$ and $h( X_{jm+2j},X_{jm+2j+1})$ are missing for $j=1,\ldots , \lfloor n_b \tau \rfloor -1\;.$ Define $I_n = \{ j(m +2)-1, j(m+2)\, | \, j=1,\ldots , \lfloor n_bt \rfloor -1 \}$. Given the infinite number of data points for $(X_t)_{t\ge 1}$, we complete the sum by letting $j=1, \ldots ,\lfloor n_b\tau \rfloor$.
  $$\begin{aligned} m \sum \limits _{j=1}^{\lfloor n_b \tau \rfloor } \left( \hat{q}_{j,m}-q \right) =&\sum _{ k \notin I_n}h( X_{k}, X_{k+1}) \quad \text {add and subtract the missing terms}\\ =&\sum _{k=1}^{\lfloor n_b \tau \rfloor (m+2) } h({\textbf {X}}_k) - \sum _{j=1}^{\lfloor n_b \tau \rfloor } \left( h({\textbf {X}}_{j(m+2)-1}) + h({\textbf {X}}_{j(m+2)}) \right) , \end{aligned}$$
  where ${\textbf {X}}_k=(X_k, X_{k+1})^{\top }$. Dividing by $\frac{1}{\sqrt{n_b(m+2)}}$
  $$\begin{aligned}&\frac{m}{\sqrt{n_b(m+2)}}\sum \limits _{j=1}^{\lfloor n \tau \rfloor } \left( \hat{q}_{j,m} -q\right) \\&= \frac{1}{\sqrt{n_b(m+2)} } \sum _{k=1}^{\lfloor (n_b (m+2)) \tau \rfloor } h({\textbf {X}}_k) \\&\quad - \frac{1}{\sqrt{n_b(m+2)}}\sum _{j=1}^{\lfloor n_b \tau \rfloor } \left( h({\textbf {X}}_{j(m+2)-1}) + h({\textbf {X}}_{j(m+2)}) \right) \\&= \frac{1}{\sqrt{n_b(m+2)} } \sum _{k=1}^{\lfloor n \tau \rfloor } h({\textbf {X}}_k) - \frac{1}{\sqrt{n_b(m+2)}}\sum _{j=1}^{\lfloor n_b \tau \rfloor } \left( h({\textbf {X}}_{j(m+2)-1}) + h({\textbf {X}}_{j(m+2)}) \right) .\\ \end{aligned}$$
  Lemma 10 yields
  $$\begin{aligned} \frac{1}{\sqrt{n } } \sum _{j=1}^{\lfloor n\, \tau \rfloor } h({\textbf {X}}_j) \overset{\mathcal {D}[0,1]}{\Longrightarrow }\ \sigma B(\tau ) \text { for }\tau \in [0,1] \;. \end{aligned}$$
  To conclude, we prove that
  $$\begin{aligned} \frac{1}{\sqrt{n_b(m+2)}}\sum _{j=1}^{\lfloor n_b \tau \rfloor } h({\textbf {X}}_{j(m+2)-1} ) + \frac{1}{\sqrt{n_b(m+2)}}\sum _{j=1}^{\lfloor n_b \tau \rfloor } h({\textbf {X}}_{j(m+2)}) =o_{\mathbb {P}}(1)\;. \end{aligned}$$
  It suffices to prove the claim for one of the two addends, as the argument is identical for both terms. For any $\varepsilon >0$,
  $$\begin{aligned}&\mathbb {P}\left( \sup _{ \tau \in [0,1]} \left| \frac{1}{\sqrt{n_b(m+2)}}\sum _{j=1}^{\lfloor n_b \tau \rfloor } h({\textbf {X}}_{j(m+2)})\right|> \varepsilon \right) \\&\quad = \mathbb {P}\left( \sup _{k=1, \ldots , n_b} \frac{1}{\sqrt{n_b(m+2)}}\left| \sum _{j=1}^{k} h({\textbf {X}}_{j(m+2)})\right|> \varepsilon \right) \\&\quad \le \mathbb {P}\left( \bigcup _{k=1}^ {n_b} \left\{ \frac{1}{\sqrt{n_b(m+2)}}\left| \sum _{j=1}^{k} h({\textbf {X}}_{j(m+2)})\right|> \varepsilon \right\} \right) \nonumber \\&\quad \le \sum _{k=1}^{n_b} \mathbb {P}\left( \frac{1}{\sqrt{n_b(m+2)}} \left| \sum _{j=1}^{k} h({\textbf {X}}_{j(m+2)})\right| > \varepsilon \right) \\&\quad \le \sum _{k=1}^{n_b} \frac{1}{n_b (m+2) \varepsilon ^2} \mathop {\operatorname {Var}}\left( \sum _{j=1}^{k} h({\textbf {X}}_{j(m+2)})\right) \;. \end{aligned}$$
  To upper bound the variance, recall that $h({\textbf {X}}_j)$ is a stationary zero-mean process, being the composition of a stationary process with a measurable function *h*. Using the definition of variance
  $$\begin{aligned} \mathop {\operatorname {Var}}\left( \sum _{i=1}^k h({\textbf {X}}_{i(m+2)}) \right)&= \sum \limits _{i=1}^k\sum \limits _{j=1}^k\mathop {\operatorname {Cov}}(h({\textbf {X}}_{i(m+2)}), h({\textbf {X}}_{j(m+2)})) \\&= k \,\gamma (0) + 2 \sum _{j=1}^{k-1} (k-j)\gamma (j(m+2)), \end{aligned}$$
  where $\gamma (k)=\mathop {\operatorname {Cov}}(h({\textbf {X}}_i),h( {\textbf {X}}_{i+k}))$. It follows that
  $$\begin{aligned} \frac{1}{k} \mathop {\operatorname {Var}}\left( \sum _{i=1}^k h({\textbf {X}}_{i(m+2)}) \right)&\le |\gamma (0)|+ 2\sum _{j=1}^{k-1}\left( 1-\frac{j}{k} \right) |\gamma (j(m+2))|\\&\le |\gamma (0)|+ 4\sum _{j=1}^{\infty }|\gamma (j)|< C\;.\\ \end{aligned}$$
  The last inequality follows from Lemma 3.5 in Furmańczyk (2007). Thus, it follows that $\mathop {\operatorname {Var}}\left( \sum _{i=1}^k h({\textbf {X}}_{i(m+2)}) \right) \le C k.$ Putting all pieces together,
  $$\begin{aligned}&\mathbb {P}\left( \sup _{\tau \in [0,1]} \left| \frac{1}{\sqrt{n_b(m+2)}}\sum _{j=1}^{\lfloor n_b \tau \rfloor -1} h({\textbf {X}}_{j(m+2)})\right| > \varepsilon \right) \\&\quad \le \sum _{k=1}^{n_b-1} \frac{1}{n_b (m+2) \varepsilon ^2} \mathop {\operatorname {Var}}\left( \sum _{j=1}^{k} h({\textbf {X}}_{j(m+2)})\right) \\&\quad \le \frac{C}{n_b (m+2) \varepsilon ^2} \sum _{k=1}^{n_b-1} k = \mathcal {O}\left( \frac{n_b^2}{n_b(m+2)} \right) \\&\quad =\mathcal {O}\left( \frac{1}{(m/\sqrt{n})^2}\right) \xrightarrow {n\rightarrow \infty } 0\;. \end{aligned}$$
(ii) Following the same argument of part (i), let us go back to the expression (set $\tau =1$)
  $$\begin{aligned} m \sum \limits _{j=1}^{n_b} \left( \hat{q}_{j,m} -q\right) = \sum _{k=1}^{n_b(m+2) } h({\textbf {X}}_k) - \sum _{j=1}^{n_b } \left( h({\textbf {X}}_{j(m+2)-1}) + h({\textbf {X}}_{j(m+2)}) \right) \;. \end{aligned}$$
  Multiplying both sides by $(n_b(m+2))^{-(\frac{1}{2}+d)}$
  $$\begin{aligned}&m(n_b(m+2))^{-\left( \frac{1}{2}+d\right) } \sum \limits _{j=1}^{n_b} \left( \hat{q}_{j,m} -q\right) = (n_b(m+2))^{-\left( \frac{1}{2}+d\right) } \sum _{k=1}^{n_b(m+2) } h({\textbf {X}}_k) \\&\quad - (n_b(m+2))^{-\left( \frac{1}{2}+d\right) } \sum _{j=1}^{n_b } \left( h({\textbf {X}}_{j(m+2)-1}) + h({\textbf {X}}_{j(m+2)}) \right) \;. \end{aligned}$$
  We firstly show that the second term converges in probability to 0. For this, note that
  $$\begin{aligned}&\mathbb {P}\left( \left| \sum _{j=1}^{n_b} h({\textbf {X}}_{j(m+2)})\right| > \left( n_b(m+2)\right) ^{(\frac{1}{2}+d)} \varepsilon \right) \\&\quad \le \varepsilon ^{-2} (n_b(m+2))^{-1-2d} \mathop {\operatorname {Var}}\left( \sum _{j=1}^{n_b } h({\textbf {X}}_{j(m+2)}) \right) \\&\quad \le C \varepsilon ^{-2} (n_b(m+2))^{-1-2d} n_b^2 \\&\quad = \mathcal {O}\left( \frac{n_b^{2}}{{(n_b(m+2))}^{1+2d}}\right) = \mathcal {O}\left( \frac{1}{\left( \frac{m}{\sqrt{n}}\right) ^2n^{2d}}\right) \xrightarrow {n\rightarrow \infty }0\;. \end{aligned}$$
  Next, we prove convergence in distribution for the first term $n^{-(\frac{1}{2}+d)} \sum _{j=1}^{n} h({\textbf {X}}_j)$ as a consequence of Theorem 10. For $\gamma \in \mathcal {T}$, let $p_\gamma ({\textbf {x}})=\mathbb {P}( {\textbf {V}}_\gamma {\textbf {X}}_1\le {\textbf {x}})$. Then,
  $$\begin{aligned}&n^{-(\frac{1}{2}+d)} \left| \sum _{j=1}^n h({\textbf {X}}_j)-q+ \sum _{ \gamma \in \mathcal {T}}\left( (\nabla p_\gamma ({\textbf {0}}) {\textbf {V}}_\gamma \right) n\bar{{\textbf {X}}}_n \right| \\&\quad =n^{-(\frac{1}{2}+d)} \left| \sum _{j=1}^n \sum _{ \gamma \in \mathcal {T}} \left( \mathbbm {1}\left( {\textbf {V}}_\gamma {\textbf {X}}_j\le {\textbf {0}}\right) -p_\gamma (0)\right) + \sum _{ \gamma \in \mathcal {T}}\left( \nabla p_\gamma ({\textbf {0}}) {\textbf {V}}_\gamma \right) \sum _{j=1}^{n} {\textbf {X}}_j \right| \\&\quad \le \sum _{ \gamma \in \mathcal {T}} n^{-(\frac{1}{2}+d)} \left| \sum _{j=1}^n \mathbbm {1}\left( {\textbf {V}}_\gamma {\textbf {X}}_j\le {\textbf {0}}\right) -p_\gamma (0) + \nabla p_\gamma ({\textbf {0}}){\textbf {V}}_\gamma \sum _{j=1}^{n} {\textbf {X}}_j \right| \xrightarrow {\mathbb {P}}0. \end{aligned}$$
  The last relation is due to the reduction principle (C19). Thus, an application of Theorem 1 in Chung (2002) yields
  D30 $$\begin{aligned} n^{-(\frac{1}{2}+d)} \sum _{j=1}^n h({\textbf {X}}_j) \xrightarrow {\mathcal {D}} \mathcal {N}(0, \sigma ^2), \end{aligned}$$
  where $\sigma ^2= \sum _{\gamma _1, \gamma _2 \in \mathcal {T}} (\nabla p_{\gamma _2}({\textbf {0}}))^\top V_{\gamma _1}V V_{\gamma _2}^\top \nabla p_{\gamma _1}({\textbf {0}}),$ with $V=\frac{\Gamma (d)^2}{\Gamma (2d+2)\cos (\pi d)} {\textbf {E}}$, and ${\textbf {E}}$ is the matrix with all entries equal 1.

$$\square $$

### Proof of Corollary 6

In order to derive the asymptotic distribution of $SC_{n_b}$, we need to find to first derive the limit of $\hat{V}_{k,n_b}$. The strategy is to write $\hat{V}_{k,n_b}$ as a functional of the process $W_{n_b}(s) := \frac{m}{\sqrt{n}} \sum _{j=1}^{\lfloor n_bs\rfloor } (\hat{q}_{j,m}-q)$, for which $W_{n_b } \overset{\mathcal {D}[0,1]}{\Longrightarrow }\ \sigma B(\tau )$; see Theorem 11. The variance estimator (30) is the sum of two terms, namely $V^2_{k, n_b}:=\frac{1}{n_b}\sum _{t=1}^k S_t^2(1,k)+\frac{1}{n_b}\sum _{t=k+1}^{n_b} S_t^2(k+1,n_b)$.

1. For $$i=1, \ldots , k$$,
  $$\begin{aligned} S_i(1,k)=\sum _{j=1}^i ( \hat{q}_{j,m} -\bar{q}_{1,k})= \sum _{j=1}^{i} \hat{q}_{j,m} -\frac{i}{k} \sum _{j=1}^k \hat{q}_{j,m}\;.\end{aligned}$$
  Denote with $\tau \in [0,1]$ the unique value satisfying $k= \lfloor n_b \tau \rfloor$. Then,
  $$\begin{aligned} \frac{1}{n_b}\sum _{i=1}^k S^2_i(1,k)&= \frac{1}{n_b}\sum _{i=1}^{\lfloor n_b \tau \rfloor } \left( \sum _{j=1}^{i} \hat{q}_{j,m} -\frac{i}{k} \sum _{j=1}^k \hat{q}_{j,m}\right) ^2\\ &= \int _0^\tau \left( \sum _{j=1}^{\lfloor n_b s\rfloor } \hat{q}_{j,m} -\frac{\lfloor n_b s \rfloor }{\lfloor n_b \tau \rfloor } \sum _{j=1}^{\lfloor n_b \tau \rfloor } \hat{q}_{j,m}\right) ^2 \, ds\;. \end{aligned}$$
  Multiplying both sides by $\left( \frac{m}{\sqrt{n}}\right) ^2$ yields
  $$\begin{aligned} \left( \frac{m}{\sqrt{n}}\right) ^2 \left( \frac{1}{n_b}\sum _{i=1}^k S^2_i(1,k)\right) = \int _0^\tau \left( W_{n_b}(s) - \frac{\lfloor n_b s \rfloor }{\lfloor n_b \tau \rfloor } W_n(\tau ) \right) ^2 \, ds, \end{aligned}$$
2. For $$i=k+1,\ldots , n_b$$
  $$\begin{aligned} S_i(k+1,n_b)=&\sum _{j=k+1}^i ( \hat{q}_{j,m} -\bar{q}_{k+1,n_b})= \sum _{j=k+1}^{i} \hat{q}_{j,m} -\frac{i-k}{n_b-k} \sum _{j=k+1}^{n_b} \hat{q}_{j,m} \;. \end{aligned}$$
  Then,
  $$\begin{aligned}&\left( \frac{m}{\sqrt{n}}\right) ^2 \left( \frac{1}{n_b} \sum _{i=k+1}^n S_i^2(k+1,n_b) \right) \\&\quad = \left( \frac{m}{\sqrt{n}}\right) ^2 \int _{\tau }^1\left( \sum _{j=k+1}^{\lfloor n_bs \rfloor } \hat{q}_{j,m} -\frac{\lfloor n_bs \rfloor -\lfloor n_b\tau \rfloor }{n_b-\lfloor n_b\tau \rfloor } \sum _{j=k+1}^{n_b} \hat{q}_{j,m} \right) ^2\, ds\\&\quad =\int _{\tau }^1 \left( W_{n_b}(s) - W_{n_b}(\tau ) -\frac{\lfloor n_bs \rfloor -\lfloor n_b\tau \rfloor }{n_b-\lfloor n_b\tau \rfloor } \left( W_{n_b}(1)-W_{n_b}(\tau ) \right) \right) ^2 \, ds\;.\\ \end{aligned}$$

Since $\frac{\lfloor n_bs \rfloor }{n_b} = s + o(1)$, we can replace all $\lfloor n_bs \rfloor$ by *s*. Finally, Theorem 5 and the continuous mapping theorem yield the desired result. $\square$

## Auxiliary results

### Theorem 12

(Theorems 2.1 and 2.2 in Furmańczyk 2007) Let ${\textbf {X}}_t$ the multivariate linear process

$$\begin{aligned} {\textbf {X}}_t = \sum _{j=0}^\infty {\textbf {A}}_{j} {\textbf {Z}}_{t-j} \end{aligned}$$

where $({\textbf {Z}}_{j})_{j \in \mathbb {N}}$ is an i.i.d. sequence with variance $\varvec{\Sigma }$ and $({\textbf {A}}_{j})_{j\in \mathbb {N}}$ is a sequence of non-random matrices in $\mathbb {R}^p$. Further, $\mathbb {E}[\Vert {\textbf {Z}}_1\Vert ^2]<\infty$, where $\Vert \cdot \Vert$ represents the standard Euclidean norm. Consider the following

1. The sequence $({\textbf {A}}_{j})_{j \in \mathbb {N}}$ satisfies the SRD condition, $\sum _{j=0}^\infty \Vert {\textbf {A}}_{j}\Vert <\infty$ for $\Vert \cdot \Vert$ induced by a vector norm $\Vert \cdot \Vert$.
2. For every $s \in \mathbb {N}$, consider the function $G_s({\textbf {x}})=\mathbb {E}\left[ g\left( \sum _{r=0}^{s-1}A_r {\textbf {Z}}_{k-r} +{\textbf {x}}\right) \right]$, where $g:\mathbb {R}^p \rightarrow \mathbb {R}$ is a measurable function such that $\mathbb {E}[ g({\textbf {X}}_1)^2] < \infty$.
  - **(Lip)** $G_s$ is uniformly Lipschitz over *s*, i.e
    $$\begin{aligned} \mid G_s(x)-G_s(y)\mid \le \text {Lip}(G_s)\Vert x-y\Vert \quad \forall x,y \in \mathbb {R}^p \end{aligned}$$
    with $\sup _{s\ge 1}\text {Lip}(G_s) < C$, where *C* is a constant independent from *s*.
  - (**Lip**$^*$) $G_s$ is definitely Lipschitz over *s*, i.e., there exists $s_0$ such that for all $s>s_0$
    $$\begin{aligned} \mid G_s(x)-G_s(y)\mid \le \text {Lip}(G_s)\Vert x-y\Vert \quad \forall x,y \in \mathbb {R}^p \end{aligned}$$
    with $\sup _{s\ge s_0}\text {Lip}(G_s) < C$, where *C* is a constant indepent from *s*.
3. $$ \sum _{i=j}^\infty \Vert {\textbf {A}}_{i}\Vert ^2 = \mathcal {O}(j^{-t})$$ for some $$t>2$$.

If (1, 2. **Lip** ) or (1, 2. **Lip** $^*$, 3) are satisfied, then

$$\begin{aligned} n^{-1/2}\sum _{j=1}^{[nt]} g({\textbf {X}}_j) \overset{\mathcal {D}[0,1]}{\Longrightarrow }\ \sigma B(t) \quad t\in [0,1], \end{aligned}$$

where $$\sigma ^2 = \mathbb {E}[g({\textbf {X}}_1)^2]+2\sum _{j=1}^\infty \mathbb {E}[g({\textbf {X}}_1)g({\textbf {X}}_{1+j})]\;.$$
